# *X. couchianus* and *X. hellerii* genome models provide genomic variation insight among *Xiphophorus* species

**DOI:** 10.1186/s12864-015-2361-z

**Published:** 2016-01-07

**Authors:** Yingjia Shen, Domitille Chalopin, Tzintzuni Garcia, Mikki Boswell, William Boswell, Sergey A. Shiryev, Richa Agarwala, Jean-Nicolas Volff, John H. Postlethwait, Manfred Schartl, Patrick Minx, Wesley C. Warren, Ronald B. Walter

**Affiliations:** The Xiphophorus Genetic Stock Center, Department of Chemistry and Biochemistry, Texas State University, 419 Centennial Hall, 601 University Drive, San Marcos, TX 78666 USA; Key Laboratory of Coastal and Wetland Ecosystems, Ministry of Education, A316 Environment and Ecology Bldg., Xiamen, Fujian 361102 China; Institut de Génomique Fonctionnelle de Lyon, Unité Mixte de Recherche 5242, Centre National de la Recherche Scientifique, Université de Lyon I, Ecole Normale Supérieure de Lyon, Lyon, France; The National Center for Biotechnology Information, National Library of Medicine, Bethesda, MD 20894 USA; Institute of Neuroscience, University of Oregon, 1425 E. 13th Avenue, Eugene, OR 97403 USA; Universität Würzburg, Physiologische Chemie I, Biozentrum, Am Hubland, and Comprehensive Cancer Center Mainfranken, University Clinic Würzburg, D-97074 Würzburg, Germany; Genome Sequencing Center, Washington University School of Medicine, 4444 Forest Park Blvd., St Louis, MO 63108 USA

**Keywords:** *Xiphophorus*, *X. couchianus*, *X. hellerii*, Genome assembly, Annotation, Single nucleotide change, Genome comparison, NGS

## Abstract

**Background:**

*Xiphophorus* fishes are represented by 26 live-bearing species of tropical fish that express many attributes (e.g., viviparity, genetic and phenotypic variation, ecological adaptation, varied sexual developmental mechanisms, ability to produce fertile interspecies hybrids) that have made attractive research models for over 85 years. Use of various interspecies hybrids to investigate the genetics underlying spontaneous and induced tumorigenesis has resulted in the development and maintenance of pedigreed *Xiphophorus* lines specifically bred for research. The recent availability of the *X. maculatus* reference genome assembly now provides unprecedented opportunities for novel and exciting comparative research studies among *Xiphophorus* species.

**Results:**

We present sequencing, assembly and annotation of two new genomes representing *Xiphophorus couchianus* and *Xiphophorus hellerii*. The final *X. couchianus* and *X. hellerii* assemblies have total sizes of 708 Mb and 734 Mb and correspond to 98 % and 102 % of the *X. maculatus* Jp 163 A genome size, respectively. The rates of single nucleotide change range from 1 per 52 bp to 1 per 69 bp among the three genomes and the impact of putatively damaging variants are presented. In addition, a survey of transposable elements allowed us to deduce an ancestral TE landscape, uncovered potential active TEs and document a recent burst of TEs during evolution of this genus.

**Conclusions:**

Two new *Xiphophorus* genomes and their corresponding transcriptomes were efficiently assembled, the former using a novel guided assembly approach. Three assembled genome sequences within this single vertebrate order of new world live-bearing fishes will accelerate our understanding of relationship between environmental adaptation and genome evolution. In addition, these genome resources provide capability to determine allele specific gene regulation among interspecies hybrids produced by crossing any of the three species that are known to produce progeny predisposed to tumor development.

**Electronic supplementary material:**

The online version of this article (doi:10.1186/s12864-015-2361-z) contains supplementary material, which is available to authorized users.

## Background

**Fig. 1:**
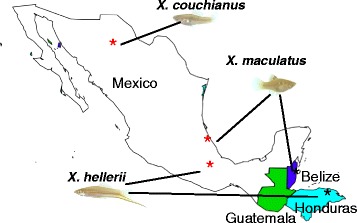
Fish used in this study. Approximate geographical distributions of three *Xiphophorus* species. The swordtail, *X. hellerii* is a male fish showing an extended caudal fin ray, while *X. maculatus* and *X. couchianus* are platyfish and do not exhibit this caudal fin extension. The stars are the locations where fishes were collected and the red stars are the location of sequenced fish originally derived

The genus *Xiphophorus* is comprised of 26 species of live-bearing platyfish and swordtails (Fig. 1). *Xiphophorus* species are found in a very broad geographical range, exceeding 2,200 km, from northern Mexico and south to Honduras. This extensive range following the Sierra Madres uplift harbors many different environments at a wide range of altitudes (e.g., sea level to 1,200 m, Fig. 1) [1]. Schartl et al. (2013) recently reported the whole-genome sequencing and assembly of a platyfish, *Xiphophorus maculatus* Jp 163 A, thus detailing the first genome architecture from a live-bearing Poeciliid fish [2]. Since publication of the platyfish genome assembly, genome resources for other Poeciliid fishes, such as *Poecilia reticulata* (guppy) [3], *Poecilia formosa* (amazon molly) and *Poecilia latipinna* (sailfin molly) are publically available or will soon be released. In addition to the whole genome, transcriptomes of *X. maculatus* have been sequenced, *de novo* assembled from RNAseq data [4, 5] and annotated using homologous coding sequences from related species (Ensembl genebuild pipeline). The availability of the *X. maculatus* reference genome and transcriptome assemblies have greatly accelerated the identification of differences that are coincidental with speciation, the evolution of genetic incompatibility, and the genetics underlying pigment pattern expression, and sex determination in *Xiphophorus* [1, 5–10].

*Xiphophorus* fishes have been used as an experimental vertebrate biomedical research model for nearly 90 years. *Xiphophorus* interspecies hybrids have been a long-standing experimental model for both spontaneous and UV or carcinogen induced melanoma [6, 7]. The first *Xiphophorus* interspecies backcross leading to spontaneous development of melanoma among interspecies backcross hybrids was described in 1927 [8]. Since this time, many other interspecies crosses have been described that produce animals displaying genetic predisposition to various types of induced tumors (i.e., require treatment of backcross hybrids to develop melanoma), and these are still actively utilized experimental models for assessment of genetic interactions leading to tumor development [6, 7].

Due to this scientific history, and an ever increasing use of *Xiphophorus* in contemporary experimental biology, the *Xiphophorus* Genetic Stock Center (XGSC) was first established in the 1930’s and has remained in continuous operation as one of the oldest live animal resource centers worldwide. Twenty-four *Xiphophorus* species and 55 pedigreed lines are maintained in the XGSC and fish lines that have been sequenced for this study are available for research upon request [1, 9].

The *X. maculatus* Jp 163 A utilized for genome sequencing was a female derived from the 104^th^ generation of sibling inbreeding within the XGSC. The *X. maculatus* Jp 163 A genome assembly comprises 20,640 scaffolds with an N50 of 1.3 Mb and the final assembled sequence length is 730 Mb [2]. More recently, a extremely dense Rad-tag map (16,114 markers) scored from *X. maculatus* Jp 163 A (x) *X. hellerii* backcross has been produced and this meiotic map aligned with the genome assembly [10]. Consolidation of the genome assembly and Rad-tag maps provides one of the most detailed and highly resolved gene maps for any vertebrate experimental model system. However, a single map remains problematic when one wishes to assess the contribution of each parental allele to complex traits that appear within interspecies backcross hybrids, such as the genes underlying induced melanoma.

Availability of new *Xiphophorus* genomic resources, coupled with the capability of producing fertile interspecies hybrids and ample polymorphic content among the varied *Xiphophorus* species, can fully unleash the potential of *Xiphophorus* as an experimental model for understanding the molecular basis of morphological and physiological differences, and the inheritance of complex traits. Herein, we report sequencing and genome assembly of *X. hellerii*, also known as a “green swordtail”, and *X. couchianus* commonly called the “Monterrey playfish”*.* These two species, in conjunction with *X. maculatus*, serve as parents in four distinct spontaneous and induced melanoma models, as well as a cross leading to increased incidence of induced retinoblastoma, neurofibrosarcoma, and Schwannoma [6, 11]. The two genome assemblies detailed herein, with the previously assembled *X. maculatus* genome, represent a system for assessing allele specific gene regulation and detailing gene-gene interactions within a varied array of *Xiphophorus* interspecies hybrids.

## Results and discussion

### Genome sequencing of *X. couchianus* and *X. hellerii*

We assembled the genomes of two *Xiphophorus* species, *X. couchianus and X. hellerii*, in four iterative steps we classify as follows: (a) assisted, (b) *de novo*, (c) merging and finally (d) chromosome formatting. Our new *Xiphophorus* assemblies show contiguity metrics equivalent to the *X. maculatus* reference genome (Table 1). In terms of total assembled bases, the *X. couchianus* and *X. hellerii* genomes have 98 % and 102 % of bases assembled in the *X. maculatus* genome, respectively. Our assembly approach used the *X. maculatus* genome to guide the scaffolding of contigs, but it also included contigs merged from the *de novo* assembly that could not be aligned as sequences to the *X. maculatus* genome. The size of the *X. hellerii* genome is larger than the *X. maculatus* genome that we suggest is mostly the result of gap filling during assisted assembly and the addition of *de novo* assembled contigs. The original *X. maculatus* reference was not gap filled with short sequences. Similarly, the higher sequence coverage of the *X. couchianus* resulted in fewer shorter contigs (less than 200 bp) and longer N50 length. However, simplicity in the genome architecture may also account for these size differences in both cases. Although the *X. maculatus* genome and two newly sequenced genomes were sequenced and assembled from different technologies (10X coverage of 454 vs. Illumina Hiseq , read lengths average ~400 bp vs. 100 bp) [2], GC content and other measures of contiguity are very similar across all three. Scaffolds of *X. couchianus and X. hellerii* were then assembled into chromosomes based on the recently published *X. maculatus* Rad-tag chromosome map [10]. At the chromosome-level of genomes, the contiguity statistics for the three genomes are very similar. Overall, the statistics of newly assembled genomes of *X. couchianus* and *X. hellerii*, are comparable to the statistics of the reference *X. maculatus* genome.

**Table 1:** Assembly statistics of genomes of three *Xiphophorus* species

Level		*X. maculatus*	*X. couchianus*	*X. hellerii*
Contig level	Number	67,070	34,765	70,798
	N50 length (Mb)	0.02	0.06	0.03
	Shortest contig	500	200	200
	GC content (%)	34.7	35.4	34.6
	Total size (Mb)	652	648	657
Scaffold level	Number	20,640	12,015	23,897
	N50 length (Mb)	1.3	1.8	1.6
	Total size (Mb)	730	711	741
Chromosome level	Number	24	24	24
	N50 length (Mb)	29.4	29.3	29.4
	Total size (Mb)	724	708	734

New advances in sequencing technologies have greatly reduced the cost of genome sequencing but more importantly the algorithms designed to derive assemblies from short sequences has significantly improved. Here we show that within a genus high quality assemblies can be cost effectively derived from about half the traditional Illumina coverage (~100x) for *de novo* assembly. Thus, it is now possible to sequence and assemble all 23 remaining extant *Xiphophorus* species with significant cost savings. To provide the two new *Xiphophorus* genomes, we used an approach that combined *de novo* and reference-guided assemblies. Here we show two independent genome assemblies were built with all sequence data, using the SOAPdenovo2 assembler and an assisted assembly from roughly 52X total input sequence coverage in whole-genome shotgun reads, a combination of 30X fragments, and 17X 3 kb, and 5X 8 kb matepairs for *X. hellerii*; and 51X total sequence coverage in whole-genome shotgun reads, a combination of 29X fragments, 14X 3 kb, and 8X 8 kb matepairs for *X. couchianus.* It is important to follow our outlined iterative steps to ensure new within genus references are not a mere syntenic reflection of the genome reference used for assisted assembly. Therefore, we contend the proliferation of additional genome references within genus can be in most cases at least as high quality as the original reference that serves as a starting point.

### Annotation of *X. hellerii* and *X. couchianus* genomes

After a genome is assembled, the next major challenge is to annotate the genome for gene content. The standard process followed is to rely on publicly available pipelines such as Ensembl [12] or NCBI (http://www.ncbi.nlm.nih.gov/refseq/) or to annotate the genome with a tool such as MAKER [13]. The state of the art is to build gene models with prior similarity evidence coupled with RNAseq data for a comprehensive set of gene predictions. Although we advocate this approach when feasible, we used an alternative approach that utilized information of gene structure from the *X. maculatus* reference genome and lifted over all possible gene models to the new genome references. Using the Ensembl gene annotation of *X. maculatus* and the RATT annotation transferring tool [14], we produced 20,300 *X. couchianus* annotated transcripts with an N50 of 3,609 bp, an average length of 2,575 bp, and a total size of 51 Mb (Table 2). The resulting inferred transcriptome of *X. couchianus* covers 99 % of the gene number and 97 % of the nucleotides of the *X. maculatus* transcriptome. Using the same method we produced 20,325 *X. hellerii* transcripts with a final N50 of 3,635 bp, average length of 2,581 bp, and a total size of 52 Mb (Table 2). The inferred *X. hellerii* transcriptome also covers 99 % of gene number and 99 % of nucleotides of the reference *X. maculatus* transcriptome.

**Table 2:** Statistics of transcriptomes of three *Xiphophorus* species

	*X. maculatus*	*X. couchianus*	*X. hellerii*
# of gene models	20,498	20,300	20,325
N50 length (bp)	3,615	3,609	3,625
Average length (bp)	2,679	2,575	2,581
Total size (Mb)	52.9	52.3	52.5

There are several reasons why the RATT tool fails to transfer some gene models to new genomes. For example, there are 174 genes annotated in the *X. maculatus* genome that were not transferred to *X. hellerii.* Attempts to manually align these gene models failed for 15 of them, three of these gene models are located in contig breakpoints, 13 of them mapped to multiple locations and the remainder can be aligned but failed one of the quality control steps during RATT transfer. Gene models aligned to new genomes but not transferred by RATT may potentially be rescued through manual curation.

The opportunity to obtain a genome reference and corresponding gene set is most desired by biologists. Previously, genome annotation required expensive computational effort, yet with the RATT genome annotation approach, the computational demands of annotating a genome are greatly reduced. In our study it requires about 10 Gb of memory and four days of manual curation steps compared with weeks of gene annotation pipeline based approaches. However, significantly shorter computational times are forthcoming that promise to speed up methods such as MAKER [13]. For the reference-based approach, there is no additional sequencing cost once the genome is sequenced and assembled, but we emphasize it does require a well-developed reference genome from a closely related species.

### Sequence variations among *Xiphophorus* genomes

**Fig. 2:**
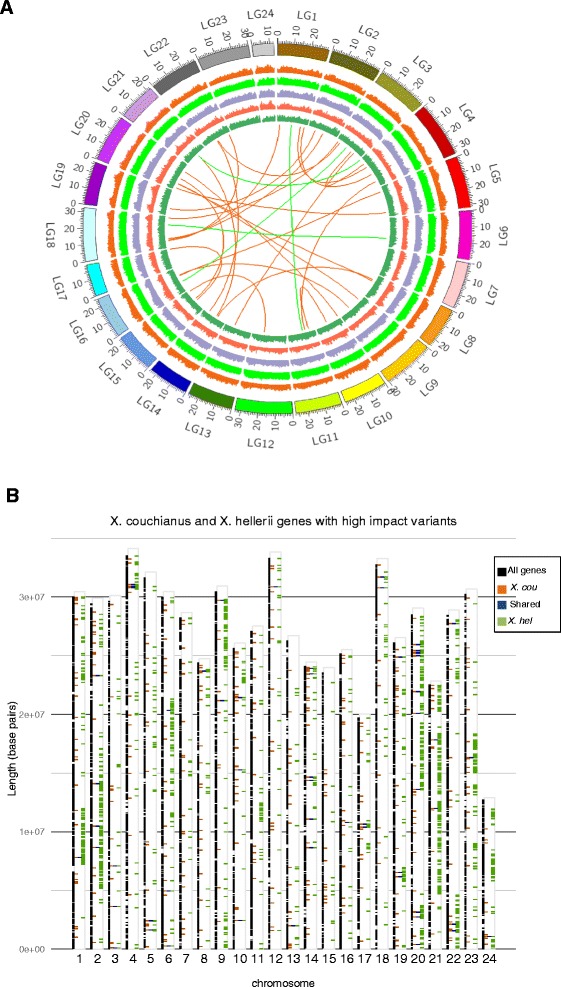
Distribution of polymorphisms in *Xiphophorus* genome among 24 chromosomes. **a** The histogram rings in the Circos plot represent the number of SNCs in 100 kb bins normalized by 3000. Tracks from outside circles to inner circles are polymorphisms between *X. maculatus* and *X. couchianus* (orange), between *X. maculatus* and *X. hellerii* (light green), only in *X. maculatus* (purple), only in *X. couchianus* (red) and only in *X. hellerii* (dark green). The connecting links in the inner circle show the inter-chromosomal rearrangements between *X. maculatus* and *X. couchianus* (orange links) and *X. maculatus* and *X. hellerii* (green links). **b** Distribution of genes with high impact polymorphisms in the genome. The black, orange, blue and green bars represent the location of all protein coding genes in *X. maculatus* genome, genes with high impact variants (see Materials and Methods) in *X. couchianus* relative to *X. maculatus*, shared genes with high high impact variants between in *X. couchianus* and *X. hellerii* relative to *X. maculatus*, genes with high impact variants in *X. hellerii* relative to *X. maculatus*

In order to determine variants among three *Xiphophorus* genomes, we aligned reads of *X. couchianus* and *X. hellerii* to the homologous sequences of *X. maculatus* reference chromosomes. For *X. couchianus*, 8,315,847 SNCs and 1,147,037 insertions and deleletions (InDels) were identified between the *X. couchianus* and *X. maculatus* genomes, corresponding to an overall polymorphic rate of about 1 base change for every 69 bases genome wide. Between *X. hellerii* and *X. maculatus*, the total number of polymorphisms identified were 10,909,727 SNCs, and 1,465,344 InDels with an overall polymorphism rate of about 1 base in every 52 bases. The frequency differences of these differences may be due to the methods utilized to maintain the *X. couchianus* (sibling line breeding) and *X. hellerii* (reciprocal breeding between two lines to maintain green and organge sword colors). We then examined the whole genome distribution of polymorphisms along the chromosomes (Fig. 2a). The polymorphisms are more abundant and evenly distributed between *X. hellerii* and *X. maculatus* (histogram in light green) than those between *X. couchianus* and *X. maculatus* (histogram in orange). Species-specific polymorphisms were also identified (Fig. 2a, three innermost histograms). The genome of *X. couchianus* (Fig. 2a, red ring) has the fewest species-specific polymorphisms compared to the other two species, reflective of the sibling line breeding origins of this sequenced individual, but the distribution of species-specific polymorphisms along chromosomes are similar in the two platyfish and as expected rates of polymorphisms are higher near the ends of chromosomes. The species-specific polymorphisms in *X. hellerii* are more evenly distributed.

In a previous study based on *de novo* assembled transcriptomes, we estimated the frequency of SNCs between *X. maculatus* and *X. couchianus* to be about 1 base in every 700 bp [4], yet an observed 1 base in 69 bp polymorphism frequency seen in this study is considerably higher. Not surprisingly, base variation is more conserved in protein coding sequences and our sensitivity is elevated as a result of deeper sequence coverage of the entire genome in contrast to the previous method that only considered polymorphisms in the transcribed sequences [4]. It will be necessary to further resequence *X. couchianus* populations to refine our preliminary estimates of genome variation.

### Structural variation among *Xiphophorus* genomes

In addition to SNCs, we also identified inter-chromosomal rearrangements among species. To call an inter-chromosomal rearrangement event, at least a 20 kb sequence from a single *de novo* assembled contig must be aligned to two different chromosomes. In total, 24 inter-chromosomal rearrangement events are found between *X. couchianus* and *X. maculatus* and 4 events are found between *X. hellerii* and *X. maculatus* (Additional file 1: Table S1 and Additional file 2: Table S2)*.* There are six times more genomic rearrangement events between *X. maculatus* and *X. couchianus* (24 vs. 4) than between *X. maculatus* and *X. hellerii.* This result does not agree with phylogenetic studies indicating *X. maculatus* and *X. couchianus* are less evolutionarily divergent. We note the *X. couchianus* contigs are on average longer than contigs of *X. hellerii* and thus more likely to detect chromosome breakpoints. With alternative computational methods for detecting large-scale variants based on paired-end reads such as Breakdancer [15] and LUMPY [12] and the resequencing of population individuals for each species, it should be possible to resolve the presence of large-scale rearrangements relative to the reference in future studies.

### Single base variation predicted to impact protein function

After identifying polymorphism locations, potential effects of them were predicted based on their relative positions to the annotated gene models and whether amino acid sequences would be expected to be altered (Table 3). Between *X. couchianus* and *X. maculatus*, most of the polymorphisms (99.02 %) are not located in the coding regions of transcripts, with intergenic, introns and UTRs exhibiting the highest percentages of polymorphisms respectively. Only a very small percentage (0.92 %) of high impact polymorphisms (e.g., stop lost, start lost and stop gained, etc.) within a species are expected to significantly alter the translated proteins. Among these variants, non-synonymous coding changes are most common. Interestingly, changes in splice junction sites are also very common, suggesting alternative splicing differences may be common between species. In addition to alternative splicing, alternative transcription start and stop sites are also commonly observed between species. How these many variants modulate protein function warrants further studies.

**Table 3:** Number and percentage of polymorphisms’ effects in *X. couchianus* and *X. hellerii* compared with the *X. maculatus* reference genome

Type	*X. couchianus*		*X. hellerii*	
	Number	Percentage	Number	Percentage
Downstream	1,542,508	12.06	1,942,329	11.71
Codon InDel	2467	0.02	2,863	0.02
Exon	211	0.00	230	0.00
Intergenic	5,007,423	39.16	6,594,737	39.79
Intron	4,103,306	32.09	5,358,720	32.33
Non synonymous coding	84,752	0.66	101,178	0.61
Splice site	35,823	0.29	44,523	0.28
Loss of start codon	116	0.00	133	0.00
Gain of stop codon	805	0.01	945	0.01
Loss of stop codon	314	0.00	445	0.00
Synonymous coding	140,717	1.10	174,526	1.05
Upstream	1,564,218	12.23	1,937,840	11.91
3'-UTR	243,474	1.90	304,241	1.84
5'-UTR	51,921	0.41	64,017	0.39
Total	12,778,055^a^	100.00	16,526,727^a^	100.00

^a^The number of effect is higher than the number of polymorphisms because one polymorphism could cause multiple effects in neighboring genes

The overall landscape of effects of polymorphisms in *X. hellerii* is very similar to *X. couchianus* (Table 3). The overall rate of variants between *X. hellerii* and *X. maculatus* is higher than between *X. couchianus* and *X. maculatus*, in accord with previous studies that suggest *X. hellerii* is more distantly related to *X. maculatus* than to *X. couchianus* [13, 16].

To test for the distributional randomness of putatively high impact gene variants in the genome, we plotted the coordinates of affected genes (Fig. 2b). Of the 452 genes in *X. couchianus* (orange bars) and 1,505 genes (green bars) in *X. hellerii* that have high impact variants relative to *X. maculatus*, we found the position of genes to be randomly distributed and are correlated with the density of localized gene models (black bars, Fig. 2b). Among these genes, 55 of them (blue bars, see Additional file 3: Table S3 for a complete list) are shared between species, suggesting fixation in the genus and are of increased scientific interest. To better understand these conserved 55 genes with high impact variants in both *X. couchianus* and *X. hellerii*, we performed GO categorical and KEGG pathway enrichment tests. Among these genes, 15 of them are annotated as uncharacterized proteins and thus prevent further biological inference. For the remaining 40 genes, GO and KEGG pathway enrichment analyses show genes associated with categories that involve regulation of homeostasis (*RYR2*, *CORIN*, *ADCYAP1R1*, *ITPR1*, *WNK2*) and response to leucine (*PIK3C3* and *UBR1*) to be significantly enriched (FDR < 0.01, Additional file 4: Figure S1 and Additional file 5: Table S4). These results may suggest evolution of the *X. maculatus* species dietary traits or preferences, or some environmental or physical parameter, that placed selective pressure on *X. maculatus* to alter its protein composition.

### Conserved synteny among three *Xiphophorus* genomes

**Fig. 3:**
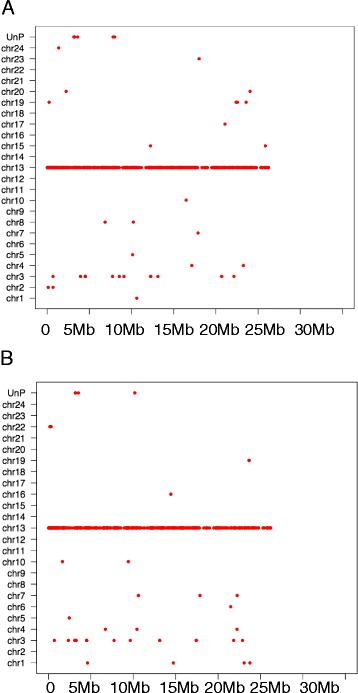
Conserved syntenies between *Xiphophorus* species. **a** The *X. couchianus* orthologs of chromosome 13 tend to lie on *X. maculatus* chromosome 13 (Y-axis) and show conserved syntenic relationship in two species. Some orthologs are mapped to other chromosomes of *X. maculatus* mostly notable chromosome 3, suggesting paralogous chromosomes arising from genome duplication. **b** The *X. hellerii* orthologs of chromosome 13 behave similarly as *X. couchianus* orthologs

To determine if the assembled *X. couchianus o*r *X. hellerii* genomes exhibit conserved synteny when compared to the *X. maculatus* genome, orthologous genes were plotted in the chromosomes of query species (*X. couchianus o*r *X. hellerii*) versus the *X. maculatus* chromosomes. The dot plots generated from this analysis suggest a one-to-one relationship for all 24 chromosomes in all three *Xiphophorus* species (Additional file 6: Figure S2 and Additional file 7: Figure S3). Although all chromosomes show strong synteny in the three species, evidence of chromosome duplication is observed in many chromosomes. For example, genes from chromosome 13 of *X. couchianus* (Fig. 3a) and *X. hellerii* (Fig. 3b) have orthologues located in chromosome 13 of *X. maculatus*; however, there are many instances where orthologues are also found in chromosome 3 of the *X. maculatus* genome. One-to-one paralogous relationship between two *X. maculatus* chromosome (Xma3/Xma13) was previously observed as result of the teleost genome duplication (TGD) [9]. The commonality of paralogy among chromosomes are also found in other teleost fishes [17–20].

### Analyses of transposable elements in *Xiphophorus* genomes

The genome of the platyfish, *X. maculatus*, was the first to provide an overview of the diversity and content of transposable elements in Poeciliid genomes [2]. Most of the TE superfamilies were identified in the different classes and subclasses (LTR, LINE, DNA) and the most active families identified from transcriptome BLAST analyses were hAT transposons and RTE (especially Rex3) LINE retrotransposons. The sequencing of two other *Xiphophorus* species provides the ability to perform comparative genomics of TEs in closely related species. We took this advantage to complete the TE library by including an automatic TE detection and to compare the diversity, content and age of TEs in the three genomes.

The newly established library contains manually annotated TE sequences and RepeatScout sequences from the previous project, combined with a RepeatModeler analyses. It includes 1,019 sequences (TEs and other types of repeats) and masks about 21 % of the southern platyfish genome (Table 4). As result some previously missing superfamilies and families were found such as Copia. However, these newly identified superfamilies make up a very small percentage of the genome (Copia covers 0.005 %) and are probably remnants of very ancient insertions. The most abundant families are Tc-Mariner and hAT DNA transposons that cover about 10 % of the genome, followed far behind by Rex-Babar and RTE retro transposons. As found in our previous study [2], LTR retro transposons compose a very small portion of the genome.

**Table 4:** Statistics of transposable elements in *Xiphophorus* genomes. Left panels: Genomes without filtration. Right panels: Genomes after removing small (less than 80 bp) and divergent (less than 80 % identify) TE elements

Class/family	Coverage(%, no filtration)			Coverage(% filtered)		
Species	*X. couchianus*	*X. maculatus*	*X. hellerii*	*X. couchianus*	*X. maculatus*	*X. hellerii*
DNA Transposons	12.348	12.267	12.013	6.212	6.023	6.022
DNA/Academ	0.019	0.206	0.021	0.016	0.016	0.017
DNA/Buster	0	0.005	0	0	0	0
DNA/CMC-Chapaev-3	0.017	0.017	0.017	0.016	0.017	0.015
DNA/CMC-Enspm	0.02	0.018	0.021	0.014	0.013	0.014
DNA/Ginger2	0	0.001	0	0	0	0
DNA/Helitron	0.256	0.252	0.256	0.157	0.153	0.162
DNA/IS4EU	0.053	0.052	0.05	0.045	0.046	0.046
DNA/Kolobok	0	0	0	0	0	0
DNA/P	0.041	0.04	0.04	0.029	0.028	0.03
DNA/PIF-Harbinger	0.634	0.619	0.614	0.568	0.557	0.555
DNA/PiggyBac	0.25	0.249	0.245	0.233	0.232	0.231
DNA/Polinton	0.024	0.025	0.029	0.014	0.015	0.017
DNA/Tc-Mariner	6.631	6.495	6.494	1.778	1.71	1.721
DNA/hAT	3.368	3.29	3.242	2.515	2.44	2.427
DNA/MITE	0.033	0.032	0.03	0.023	0.021	0.02
Unclassified	1.002	0.966	0.954	0.804	0.775	0.767
LINE Retrotransposons	2.576	2.417	2.411	1.678	1.572	1.536
LINE/I-Nimb	0.057	0.055	0.055	0.025	0.024	0.023
LINE/Jockev	0.058	0.058	0.058	0.031	0.05	0.031
LINE/L1	0.125	0.106	0.124	0.064	0.06	0.061
LINE/L2	0.942	0.905	0.899	0.659	0.623	0.624
LINE/R2	0.001	0.003	0.001	0.001	0.002	0.001
LINE/R4	0.016	0.013	0.012	0.007	0.003	0.002
LINE/RTE	0.563	0.537	0.536	0.343	0.326	0.321
LINE/Rex-Babar	0.756	0.687	0.671	0.526	0.464	0.453
PLE/Penelope	0.004	0.004	0.004	0.001	0	0
Unclassified	0.054	0.049	0.051	0.021	0.02	0.02
LTR Retrotransponsons	0.632	0.592	0.635	0.316	0.253	0.333
LTR/BEL-Pao	0.036	0.035	0.033	0.006	0.008	0.007
LTR/Copia	0.005	0.005	0.005	0.002	0.002	0.002
LTR/DIRS1-Ngaro	0.129	0.105	0.12	0.053	0.047	0.046
LTR/ERV	0.113	0.118	0.14	0.08	0.083	0.109
LTR/ERV1	0.01	0.01	0.01	0.008	0.008	0.009
LTR/Gypsy	0.234	0.219	0.228	0.09	0.077	0.086
Unclassified	0.105	0.1	0.099	0.077	0.028	0.074
SINE Retrotransposons	0.611	0.524	0.567	0.395	0.315	0.347
SINE	0.188	0.144	0.181	0.135	0.093	0.117
SINE/Hpa	0.004	0.005	0.005	0	0.001	0
SINE/MIR	0.114	0.112	0.112	0.059	0.058	0.061
SINE/V	0.238	0.196	0.203	0.16	0.121	0.128
SINE/tRNA	0.067	0.067	0.066	0.041	0.042	0.041
Unknown	5.657	5.58	5.502	4.012	3.947	3.903
Total	21.824	21.38	21.128	12.613	12.11	12.141

The *X. couchianus* and *X. hellerii* genomes were analyzed using the same library. Incomplete sequences in *X. maculatus* were manually verified or completed before analyses. By comparison, the three *Xiphophorus* genomes seem to be very close in terms of diversity and content of TEs (Table 4) containing 21.38 % (*X. maculatus*), 21.13 % (*X. hellerii*) and 21.8 % (*X. couchianus*) of TEs, respectively.

For the three genomes, TE sequences smaller than 80 nucleotides and sharing less than 80 % identity with reference sequences from the library were discarded. After filtering, TEs comprised about 12 % of the genomes.

**Fig. 4:**
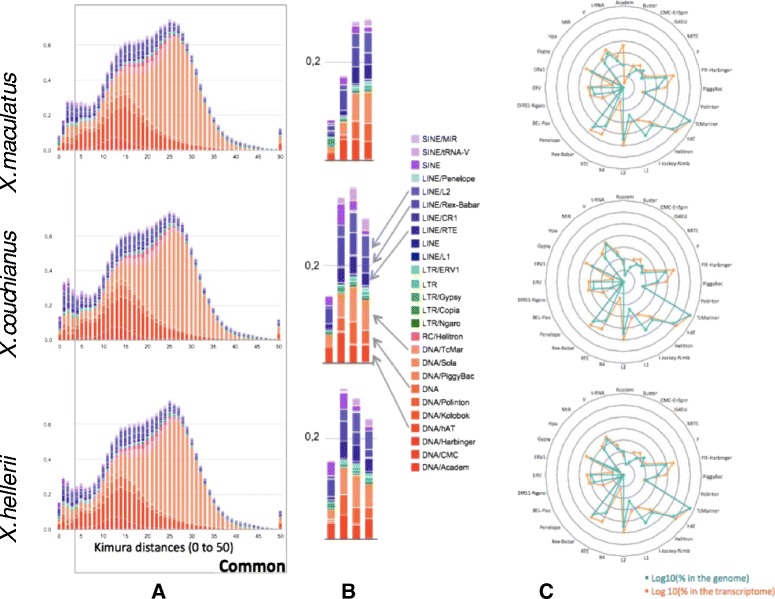
History of transposable element superfamilies in the three *Xiphophorus* genomes and potential respective current activity. **a** Kimura distances of the different superfamily copies (recent copies on the left, ancient copies on the right). **b** Focus on first Kimura distances highlighting recent copies specific to each species. **c** Spider graph representing the TE superfamilies content in the genome (Log10 [% of the genome], orange) and in the transcriptome (Log10 [% of the transcriptome], blue)

To better investigate the potential activity of the different families and the potential age of the sequences, we calculated Kimura distances of the inserted copies, with the hypothesis that mutations altering TE-inserted copies are neutral (Fig. 4a–c). In Fig. 4, recently inserted copies are located on the left side of the graph (weak Kimura values) while older copies are on the right side. The landscape of TE-copy distribution along Kimura distances is very similar or identical in the three species, especially from K-value 4 to 50. This common pattern may represent the ancestral TE landscape of the *Xiphophorus* genus while a recent K-value may represent species-specific TE activity. A focus on these recent values (Fig. 4b) highlights the main differences especially in the two first values. Indeed, a strong decrease can be observed for *X. maculatus* compared to others. By this analysis, we also evaluate which superfamilies are still active.

We also searched for TEs in the inferred transcriptomes. We found that 5 to 6 % of the transcriptomes are derived of TEs. This result is quite similar to the 4.8 % previously found for *X. maculatus* [2]. The most represented families are Tc-Mariner and hAT, as observed in the genome, followed by Jockey, LINE2, Rex-Babar and Helitron. Some superfamilies are not found in the transcriptomes, such as Copia retrotransposons.

Finally, we represented the quantity (Log[content %]) of each superfamily in both the genome and transcriptome, in a spider graph to observe the relationship between genome copy number and TE quantity in transcriptomes. In the case of basal transcription, we expect proportionality between the number of copies in the genome and the quantity of copies in the transcriptome. A family with a high copy number in the genome should be highly represented in the transcriptome. In this way, we highlight superfamilies that could be over-represented in transcriptomes compared to their respective quantity in genomes.

At first glance, genome and transcriptome spider graphs look very similar. For the three species, the most abundant superfamilies in the genomes are Tc-Mariner, hAT, L2, Rex-Babar, PIF-Harbinger and RTE. In transcriptomes, Tc-Mariner, hAT, I-Nimb-Jockey, L2, Rex-Babar and Helitron are the most represented superfamilies. Our spider graphs show that Tc-Mariner, hAT, L2 and Rex-Babar are indeed highly repeated in genomes and represented in transcriptomes. Many copies of these families are probably still active since they are located in recent bursts (Fig. 4b). We can point out interesting cases, as PIF-Harbinger, PiggyBac, L1, RTE or BEL-Pao that are more represented in transcriptomes. This is also the case for Academ transposons in the southern platyfish. Those could be real expression and not basal transcription. However, this requires more rigorous testing. Inversely, for Jockey and MITE, we observe an under-representation in the transcriptomes.

## Conclusions

In the work presented a variety of genomic and transcriptomic resources and methods were employed to sequence, assemble and compare genomes of two new *Xiphophorus* species, *X. couchnianus* and *X. hellerii*, with that of *X. maculatus* Jp 163A.

The traditional strength of the *Xiphophorus* experimental model involves the non-biased assessment of genetic inheritance patterns associated with complex phenotypes within intact animals. The high genetic variability among *Xiphophorus* species and capability of producing fertile interspecies hybrids allows inheritance of any observable trait to be followed into individual backcross hybrid progeny.

Improvement of genomic capabilities for the *Xiphophorus* genetic system, as undertaken herein, promises to produce new fundamental knowledge regarding shifts in the genetic regulation within interspecies hybrids that produce altered gene expression patterns in complex traits. The genome sequences and assemblies for the species utilized herein (*X. maculatus, X. couchianus,* and *X .hellerii*) will allow researchers the capability to mechanistically dissect traits that appear among progeny from interspecies crosses between any pair of these three species. For example, interspecies crosses between pairs of these three species are known to produce several distinct experimental models for induction and progression of melanoma [5, 6]. The ability to obtain both the genome and transcriptome sequences of both parental species involved in an interspecies cross will allow unequivocal assessment of the expression of every allele, from either parent, within individual F_1_ or backcross hybrid progeny.

The large-scale identification of polymorphisms in genomes provides researchers with resources to further investigate and characterize Poeciliid genomes and to provide more precise analyses of genetic diversity and speciation. Such information is crucial to identification of key regulators of important complex biological traits, such as the etiology of pigment pattern compartmentalization and adaptation to divergent environmental conditions and stressors. Previous studies in *Xiphophorus* have associated several traits to defined DNA segments in the genome. The tumor suppressor of interspecies hybrid melanoma, termed *Diff*, or *R* [21], the P locus, controlling age and size at sexual maturation [22], and the various mechanisms employed by different *Xiphophorus* species for sexual differentiation serve as a few examples of well defined complex traits that can be better understood with structural characterization of the genomic regions from new species. Historically, the lack of good genetic markers has prevented fine mapping the structural regions harboring loci associated with these interesting biological events. The newly sequenced and assembled genomes and ample polymorphisms identified present opportunity to define the size of the effective genomic regions and to highlight gene candidates. Altogether, the benefits of having three high quality genomes may represent a key to finding answers of many long-standing biological questions in *Xiphophorus*.

## Methods

### Fishes utilized

All fishes utilized were supplied by the *Xiphophorus* Genetic Stock Center, Texas State University, San Marcos, TX (http://www.*Xiphophorus*.txstate.edu). The *X. maculatus* Jp 163 A [pedigree Jp 163 A104(A)] was in its 104^th^ generation of sibling inbreeding, while the *X. couchianus* [pedigree Xc77(B)] was in its 77^th^ generation of inbreeding. The *X. hellerii* (Sarabia) [pedigree 11317] stock is maintained by reciprocal cross breeding between two distinct *X. hellerii* strains differing by sword color (orange or green sword). In all cases, a single female was utilized for DNA isolation as described in [2]. *X. maculatus*, a Southern platyfish, was originally collected in 1939 from the Rio Jamapa in Veracruz, MX. Representatives of this species have also been found in several places throughout Mexico ranging southward to Guatemala (Fig. 1). The Northern platyfish, *X. couchianus*, was collected in 1961 near Nuevo Leon, MX, and due to urban expansion is very likely extinct in the wild. The swordtail, *X. hellerii*, was originally collected in 1963 from Rio Sarabia, Oaxaca, MX. This species exhibits a very large range from central Mexico southward to Honduras (http://www.*Xiphophorus*.txstate.edu/stockcenter/stockcentermanual.html).

All animal studies were approved by the Texas State University Institutional Animal Care and Use Review Board (IACUC protocol # 201498170). All fish used in this study were from aquaria housed stock and were kept and sampled in accordance with the applicable national legislation regulations governing animal experimentation

### Genome sequencing and assembly

Genomic DNAs of *X. couchianus* and *X. hellerii* were sequenced on an Illumina Hiseq2000 platform using libraries with tiered insert sizes from 300 bp to 8 kb. After standard quality filtering steps, over 700 and 360 million 100 bp paired-end reads were obtained for *X. couchianus* and *X. hellerii,* respectively. Genome assembly occurred in three phases, first *de novo* assembly of all sequences using SOAPdenovo [23] (Additional files 8 and 9), assisted assembly using phased alignment to the *X. maculatus* reference and finally a merge of the two independent assemblies. The assembly methods utilized are similar to those used in [23]. This later merge process ensures unaligned sequences are incorporated as *de novo* assembled contigs or scaffolds, following strict alignment criteria [23]. Prior to assembly submission each assembly is gap filled and cleaned of vector and contaminating contigs.

*De novo* assembled contigs and Illumina reads were aligned to the *X. maculatus* reference genome with a novel multi-phase aligner (SRprism; ftp://ftp.ncbi.nlm.nih.gov/pub/agarwala/srprism), and then using a heuristic governed space, search attempts were made to fill scaffold gap space. SRprism reported that all alignments were of equally good quality. Filtering was performed by first identifying the histogram for per library insert size observed in alignments, deciding which range to use (usually the tightest or 99th percentile), and then by retaining paired reads that had the correct orientation and an insert size in the desired range. Next, the filtered reads were mapped to build consensus contigs, by locating consecutive contigs that were bridged by mate pairs having 30 mers each side of the gap. Then *de novo* assembly in gaps was performed between bridged contigs, and 30 mers from reads were used to build an index for *de novo* assembly. Only filtered reads and reads mapped to contig ends went into the *de novo* assembly index. Predefined maximum gap size and the number of iterations were used to limit resources spent on any particular gap. A final step was to find structural differences between built scaffolds and the reference using paired reads with mates on different scaffolds and then to perform *de novo* gap filling between reordered scaffolds. Overall, the scaffold level genomes of *X. couchianus* and *X. hellerii* consisted of 45,442 and 71,868 scaffolds with total size of 715 Mb and 746 Mb, respectively.

To allocate assembled scaffolds to chromosomes, the existing *X. maculatus* genome with 24 cytogenetically identified chromosomes [10] was used as the reference to order and orient scaffolds for *X. couchianus* and *X. hellerii* using Nucmer3.0 [24], with the parameters of minimum cluster match length of 400 bp and max gap size of 500 bp. After Nucmer alignment, *de novo* assembled scaffolds of *X. couchianus* and *X. hellerii* were placed using a custom Perl script based on nucleotide alignment position of *X. couchianus* and *X. hellerii* scaffolds relative to the *X. maculatus* chromosomes. Scaffolds or contigs that could not be placed onto chromosomes were collected into a file called “unplaced”.

### Sequences and accession codes

All sequence data have been deposited in the NCBI database under the accession numbers listed below:

**Table:** 

BioProject	BioSample	Accession	Organism
PRJNA290781	SAMN03922721	LNCC00000000	*Xiphophorus couchianus*
PRJNA290782	SAMN03968850	LNCB00000000	*Xiphophorus hellerii* (Sarabia)

Genome assembly annotations for both genomes and AGP files for ordered scaffolds are available at the *Xiphophorus* Genetic Stock Center webpage (http://www.xiphophorus.txstate.edu/).

### Transcriptome annotation

For the *X. maculatus* transcriptome, cDNA sequences were downloaded from Ensembl (Build 71). Manually annotated genes (569) were compared with the Ensembl transcriptome, and sequences missing from Ensembl were added to the enhanced version of the *X. maculatus* transcriptome. To build the transcriptomes of *X. couchianus* and *X. hellerii*, scaffold version genomes of these two query species were aligned to the *X. maculatus* genome using Nucmer3.0 [24] with parameters implemented by Rapid Annotation Transfer Tool (RATT) [14] for transferring annotations between species.

### Genome annotation

Using RATT [14] synteny between the reference and the query, InDels were established and identified between species to avoid frame shifts between two species. Gene models from *X. maculatus* were then transferred and corrected onto *X. couchianus* and *X. hellerii* genomes by RATT. The total of 20,482 gene models annotated in *X. maculatus*, resulted in transfer of 20,300 and 20,325 gene models over to *X. couchianus* and *X. hellerii*, respectively. Custom Perl scripts were used to make RATT executable on multiple threads and convert the RATT output to the latest EMBL format implementation.

### Genome synteny

To analyze conserved syntenies between species, we constructed dot plots based on orthologs identified by RATT lift over results and reciprocal best-BLAST alignment of transcriptomes. Positive orthologs and paralogs were plotted on the chromosomes based on the coordinates of the same species and the chromosome index of the other species.

### Identification and annotation of variants among *Xiphophorus* species

To identify genome wide variants, sequences of each species were trimmed using Flexbar [25] and were aligned to the reference assembly from which they were derived using BWA-mem [26]. Varscan 2.3 [27] was used to detect Single Nucleotide Changes(SNC) and InDels from alignment results with minimum coverage of three reads and a p-value cutoff of 0.1.

For all variants the potential altered protein functions were predicted using SnpEff 3.3 h [28]. The high impact variants are defined as causing one of the following events: chromosome (over 1 % of the chromosome), exon deleted, frame shift, rare amino acid, splice site acceptor, splice site donor, stop lost, start lost and stop gained [28].

### Pathway enrichment analyses

Genes with high impact variants identified among three *Xiphophorus* species were further tested for significant association with known canonical pathways. We define variants that change coding sequences among species as critical variants. HGNC gene symbols annotated from Ensembl or top BLAST hit (NBCI non-redundant protein database, e-value cutoff E-10) for genes that were not annotated in Ensembl were used for functional analyses. The WEB-based GEne SeT AnaLysis Toolkit (WebGestalt) database was used for functional characterization and classification of gene symbols harboring high impact variants [29]. Enriched functional groups and pathways were identified by the Benjamini & Hochberg method for Multiple Test Adjustment [30].

### Analyses of transposable elements

Transposable elements were investigated in the three genomes and transcriptomes using the previously established library [2]. This library was further enhanced by automatic annotation using RepeatScout [31] and RepeatModeler (http://www.repeatmasker.org/RepeatModeler) employing default parameters. All detected sequence redundancies were discarded. Genome assemblies and transcriptomes were masked using RepeatMasker 3.3.0 [A.F.A. Smit, R. Hubley & P. Green, unpublished data] with default parameters, and RepeatMasker outfiles (“.out”) were parsed, using a custom perl script, to establish repeat coverage and copy numbers. The number and coverage of repeat sequences smaller than 80 nucleotides and with less than 80 % of identity with the reference sequence were also established to determine the quantity of small sequences in *Xiphophorus* genomes. Kimura distances between genome sequences were calculated to evaluate the age (divergence) of TE copies. This analysis assumes that most TE copies would be silenced by the host genome after insertions and would accumulate neutral mutations. The proportions of transversions (corresponding to purine-purine or pyrimidine-pyrimidine mutations, noted “q”) and transitions (purine-pyrimidine mutations, noted “p”) were calculated based on the alignment between genome copies and sequences that match in the library. Rates of transversions and transitions were transformed as Kimura distances using [K = − ½ ln(1 – 2p – q) – ¼ ln(1 – 2q)].

## Additional files

Additional file 1: Table S1. 24 inter-chromosomal rearrangement events between *X. couchianus* and *X. maculatus*. (XLSX 49 kb) Additional file 2: Table S2. 4 inter-chromosomal rearrangement events between *X. hellerii* and *X. maculatus*. (XLSX 40 kb) Additional file 3: Table S3. Genes with high impact variants in both *X. couchianus* and *X. hellerii* genomes compared with *X. maculatus*. (XLSX 48 kb) Additional file 4: Figure S1. Relationship of GO categories that are enriched in genes with high impact variants. (PPTX 93 kb) Additional file 5: Table S4. GO categories that are enriched in genes with high impact variants. (XLSX 48 kb) Additional file 6: Figure S2. Dot plots of location of one-to-one orthologues in the 24 chromosomes of *X. couchianus* and *X. maculatus*. (PDF 110 kb) Additional file 7: Figure S3. Dot plots of location of one-to-one orthologues in the chromosomes of *X. hellerii* and *X. maculatus*. (PDF 111 kb) Additional file 8:
**An AGP file describes the assembly of chromosomes of** ***X. couchianus*** **from contigs.** (AGP 7629 kb) Additional file 9:
**An AGP file describes the assembly of chromosomes** ***X. hellerii*** **from contigs.** (AGP 11213 kb)
