# Supplementary figures and images for: X. couchianus and X. hellerii genome models provide genomic variation insight among Xiphophorus species
Source: BMC Genomics. 2016 Jan 7;17:37. doi: 10.1186/s12864-015-2361-z (PMC4705583; doi:10.1186/s12864-015-2361-z)

## Slide 1
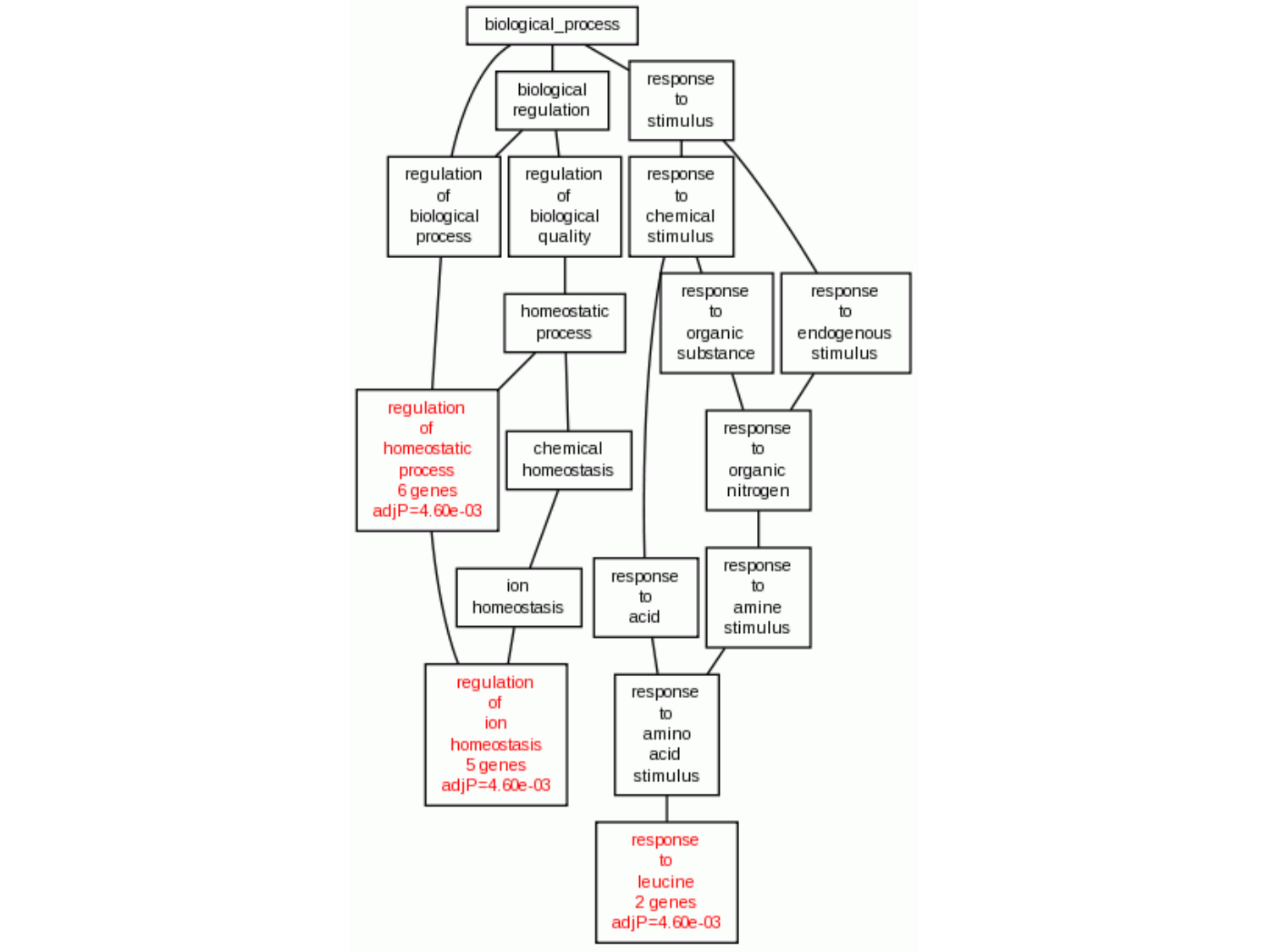

Supplement: Additional file 4: Figure S1. — Relationship of GO categories that are enriched in genes with high impact variants. (PPTX 93 kb) [file 12864_2015_2361_MOESM4_ESM.pptx]

# LG1

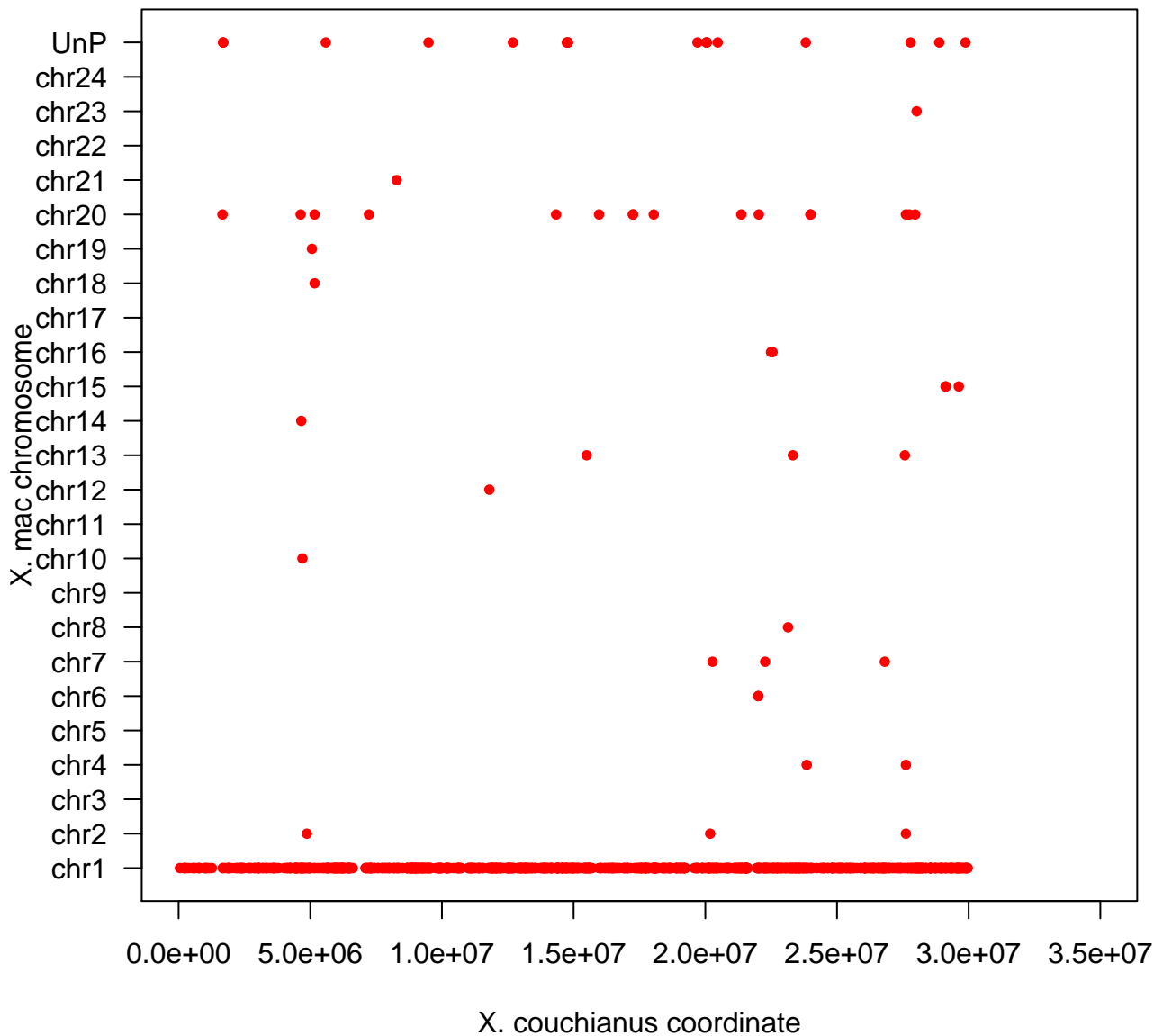

# LG10

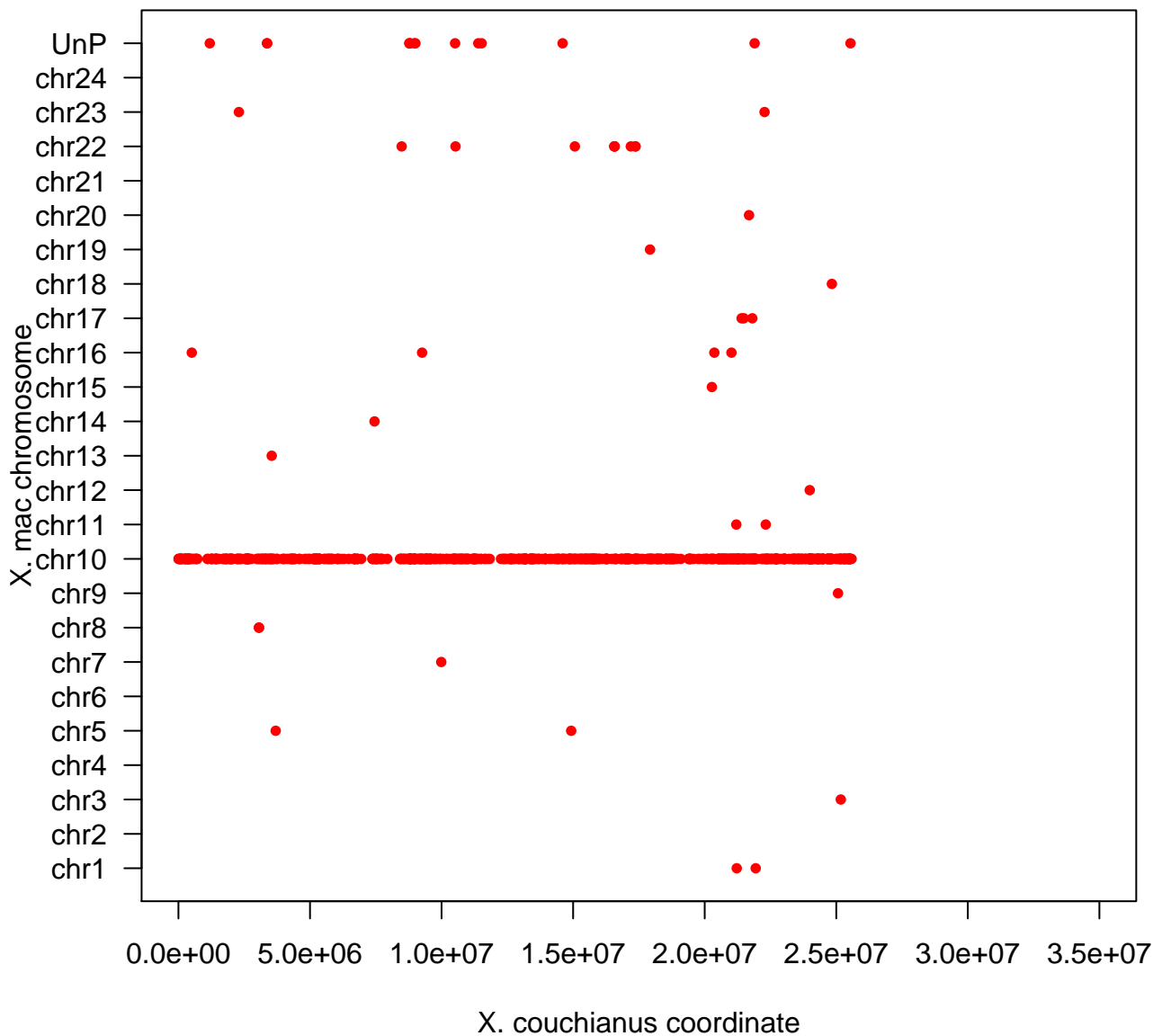

# LG11

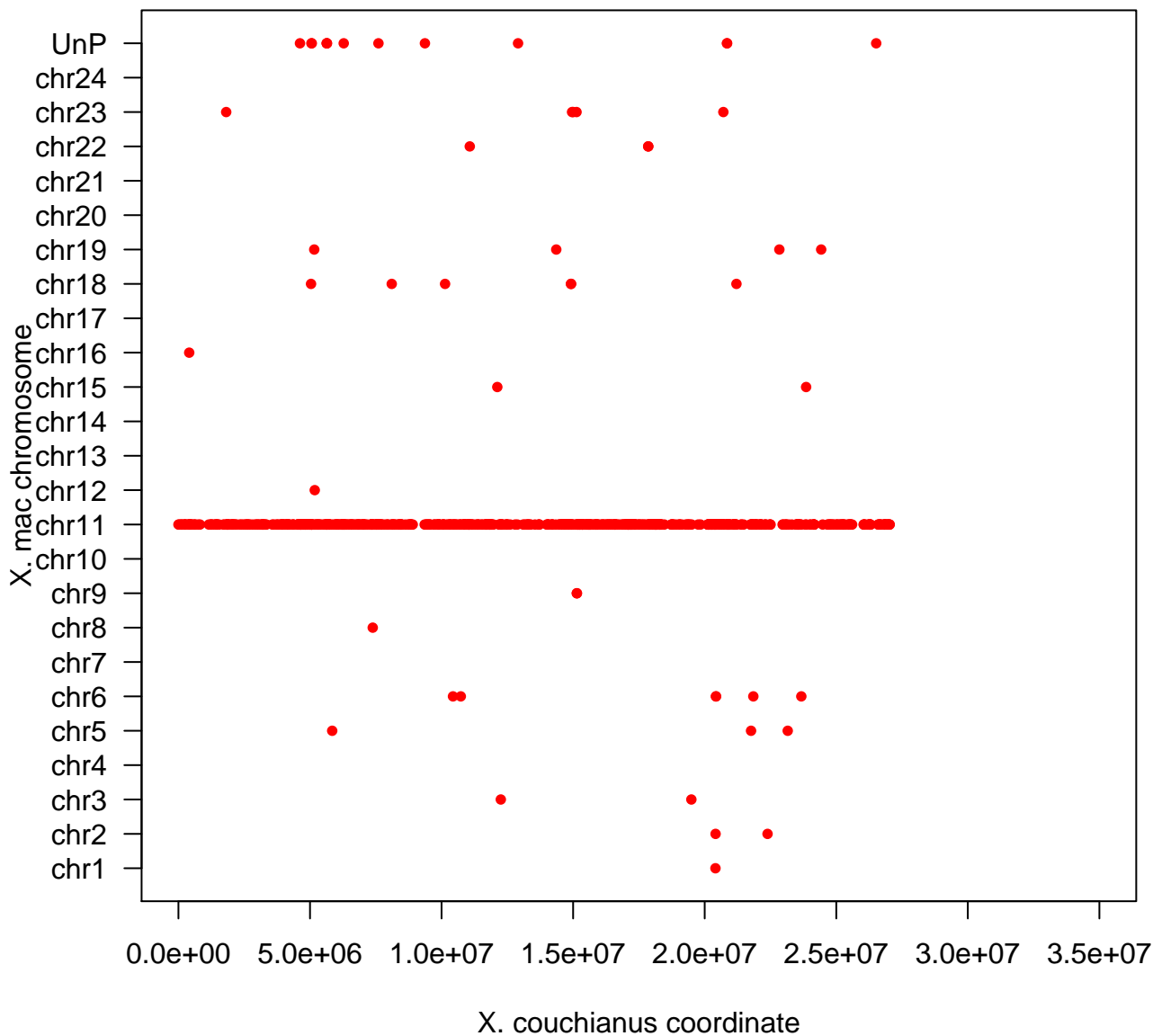

# LG12

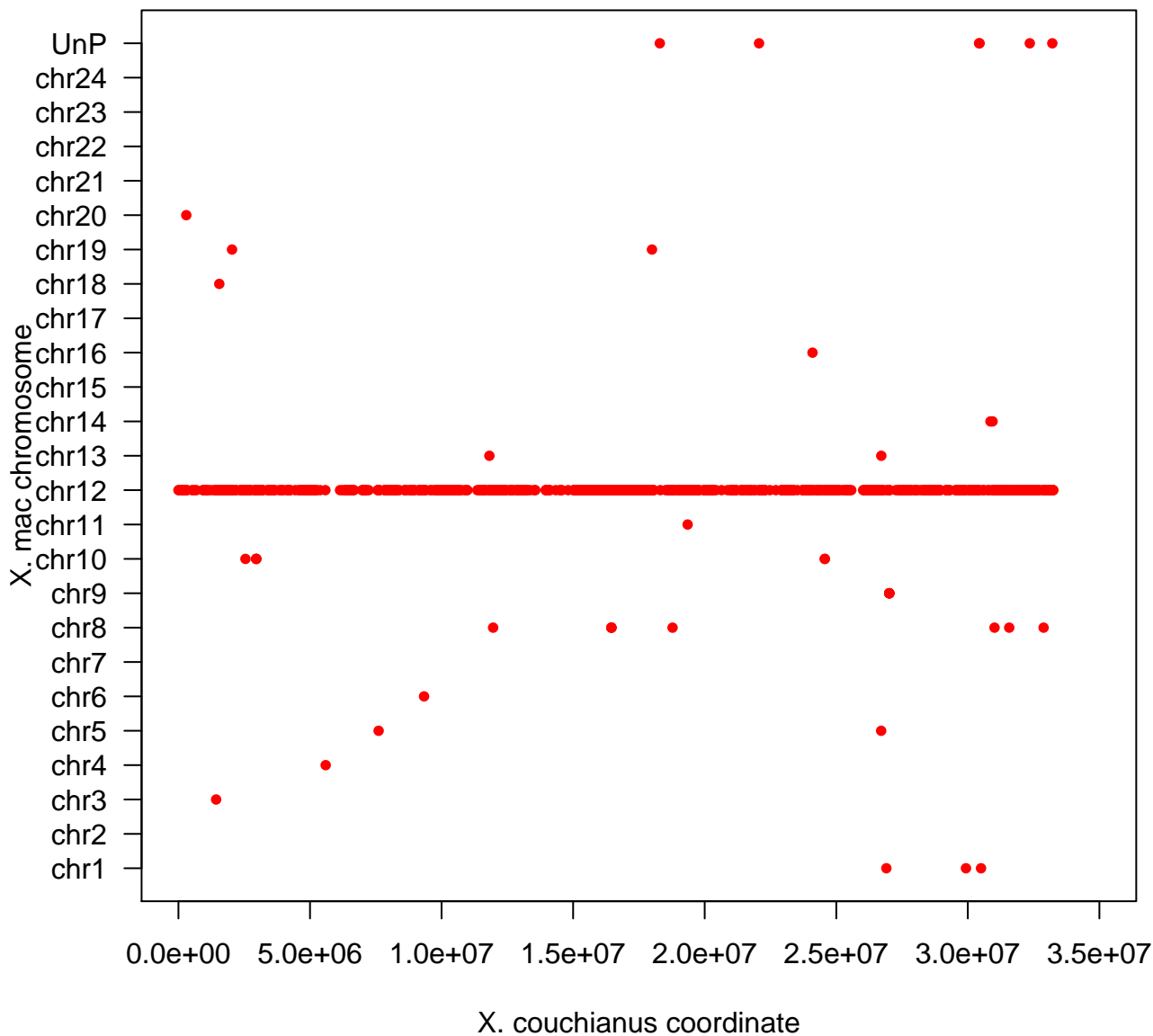

# LG13

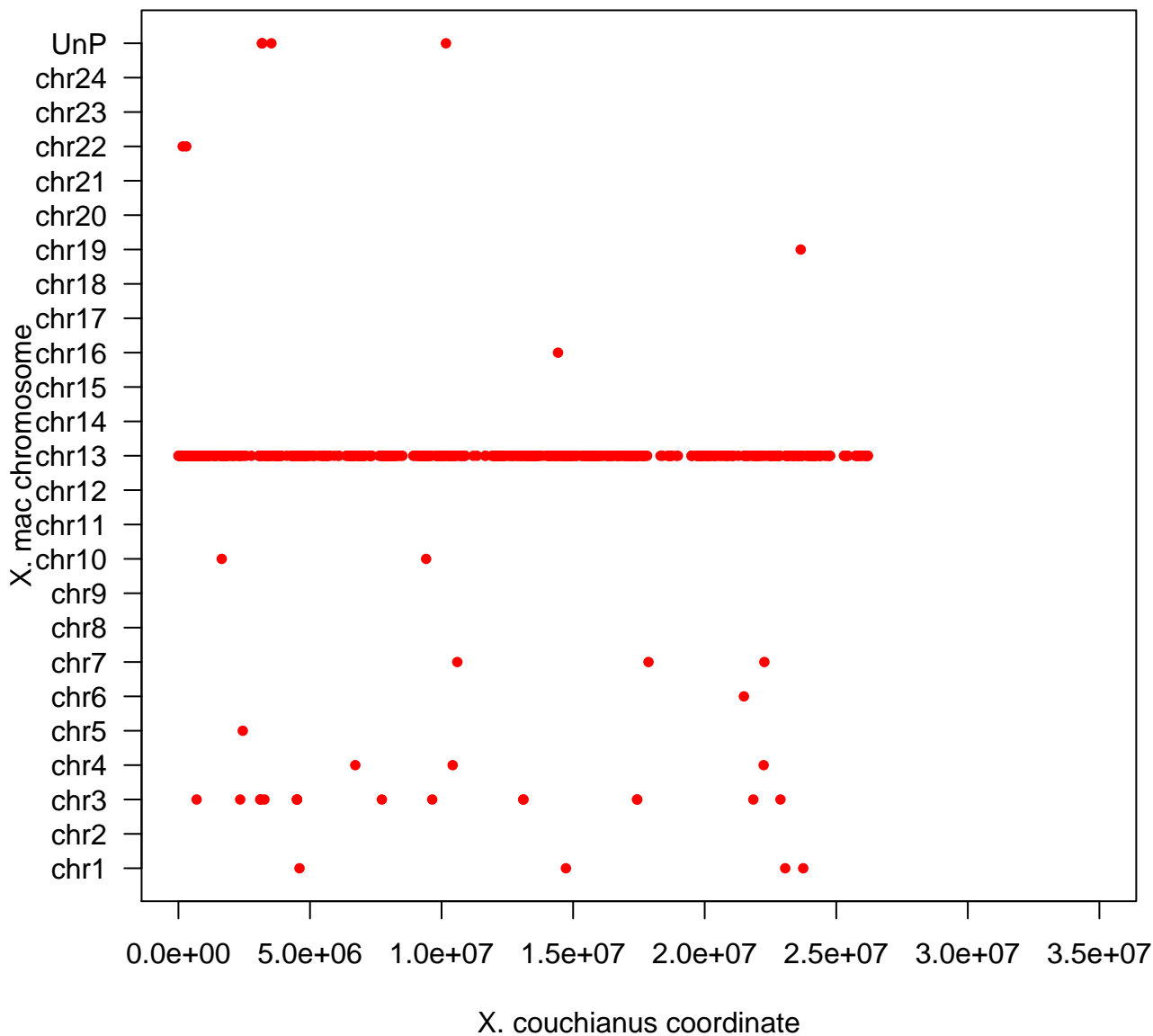

# LG14

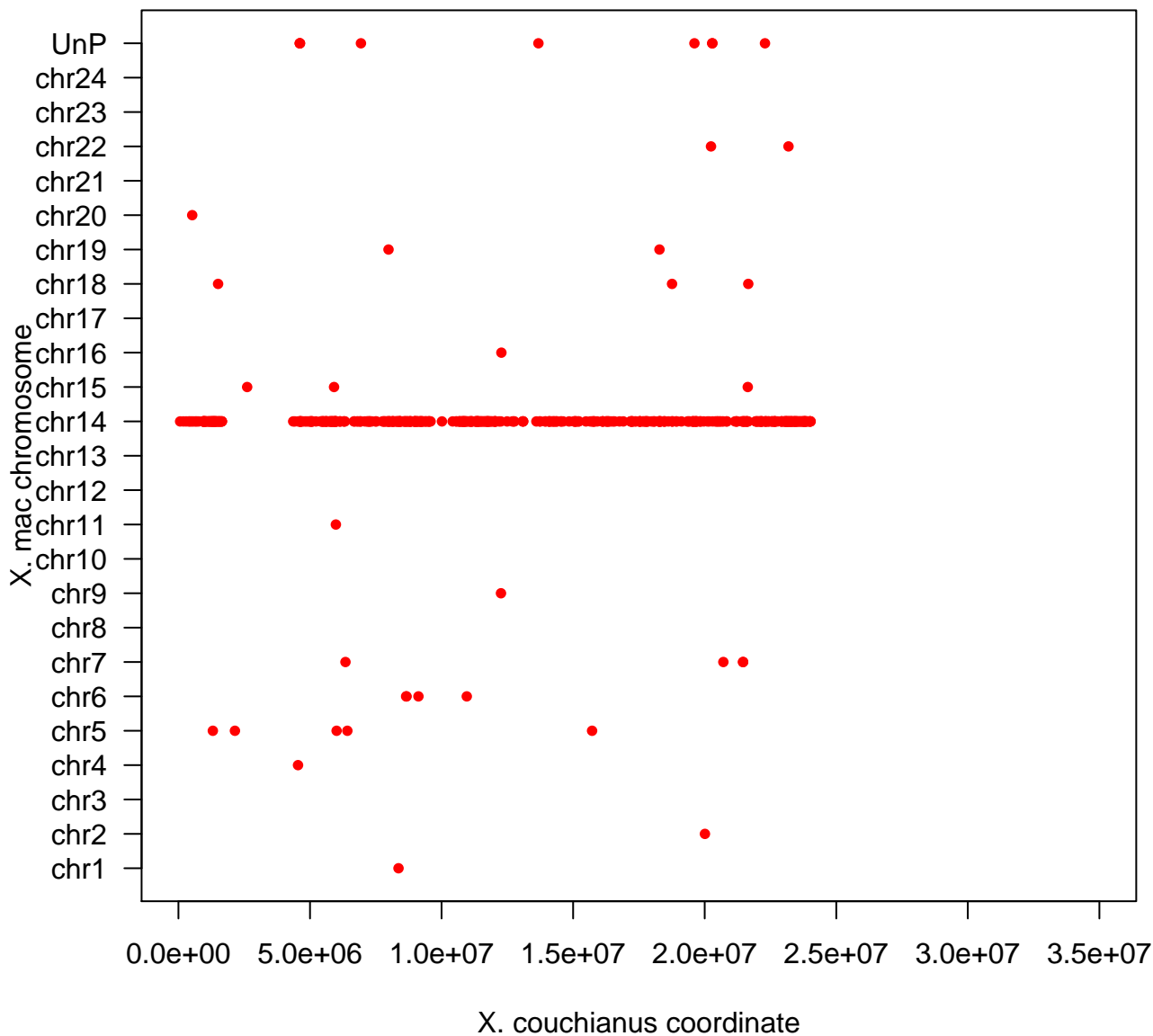

# LG15

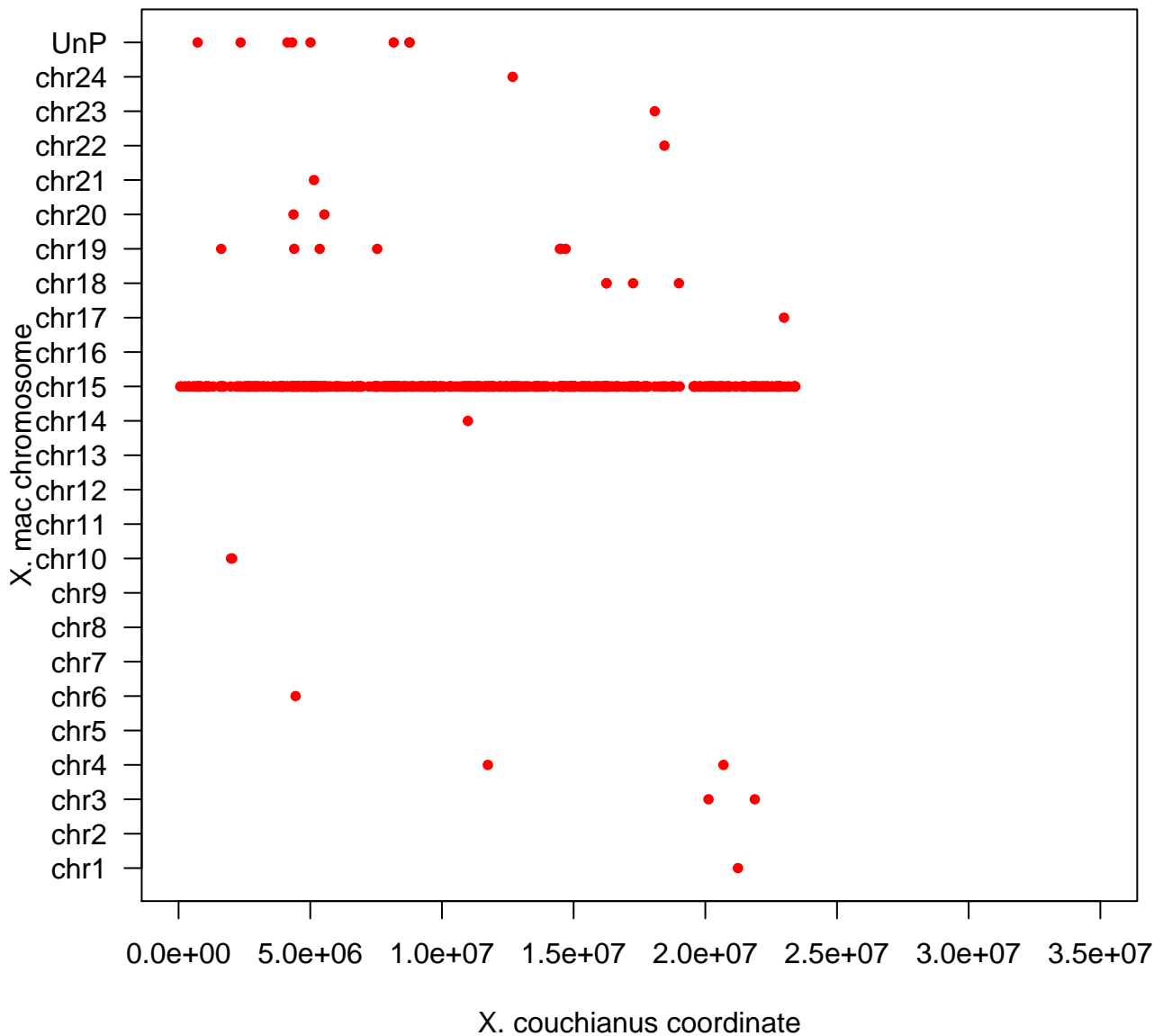

# LG16

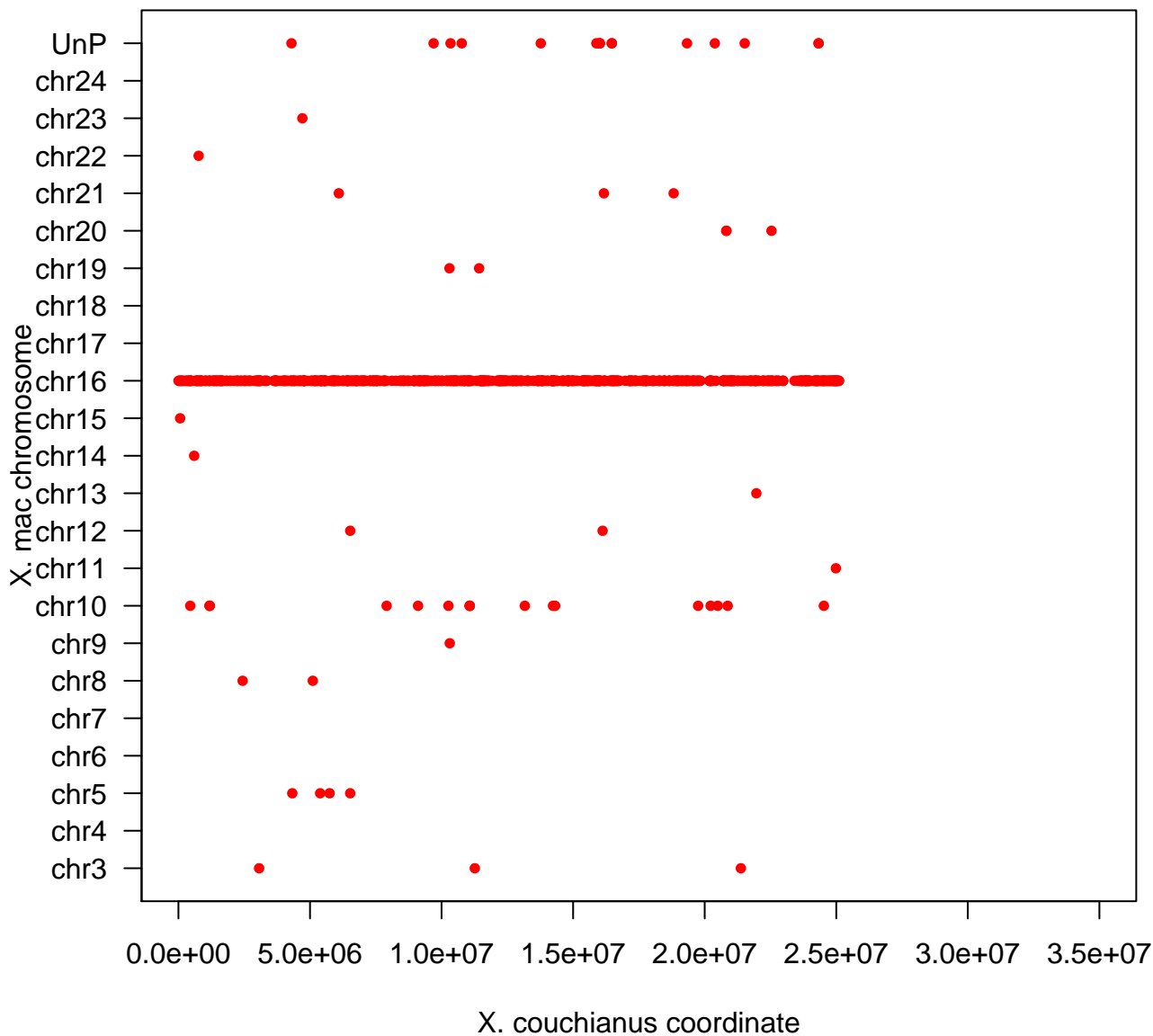

# LG17

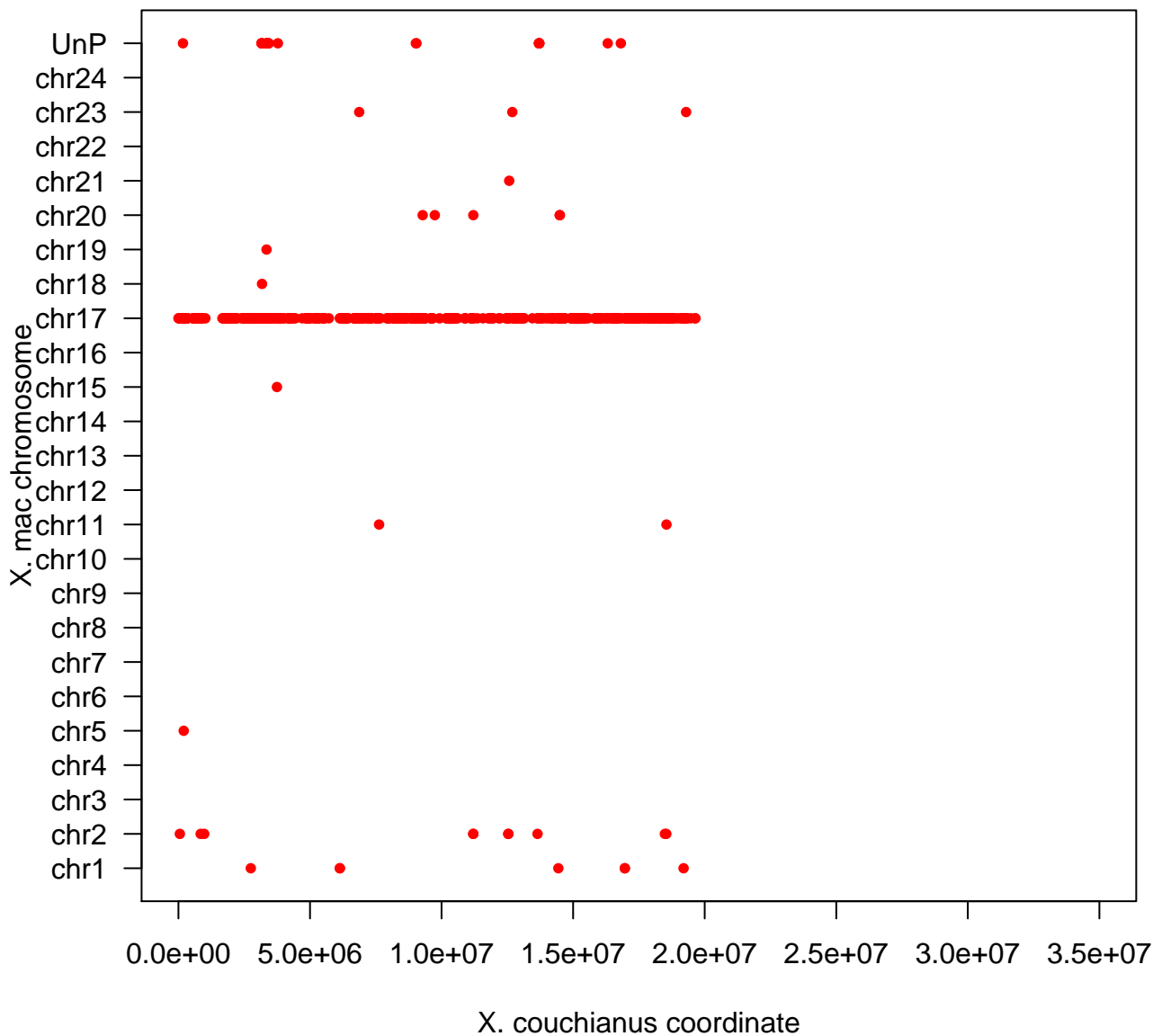

# LG18

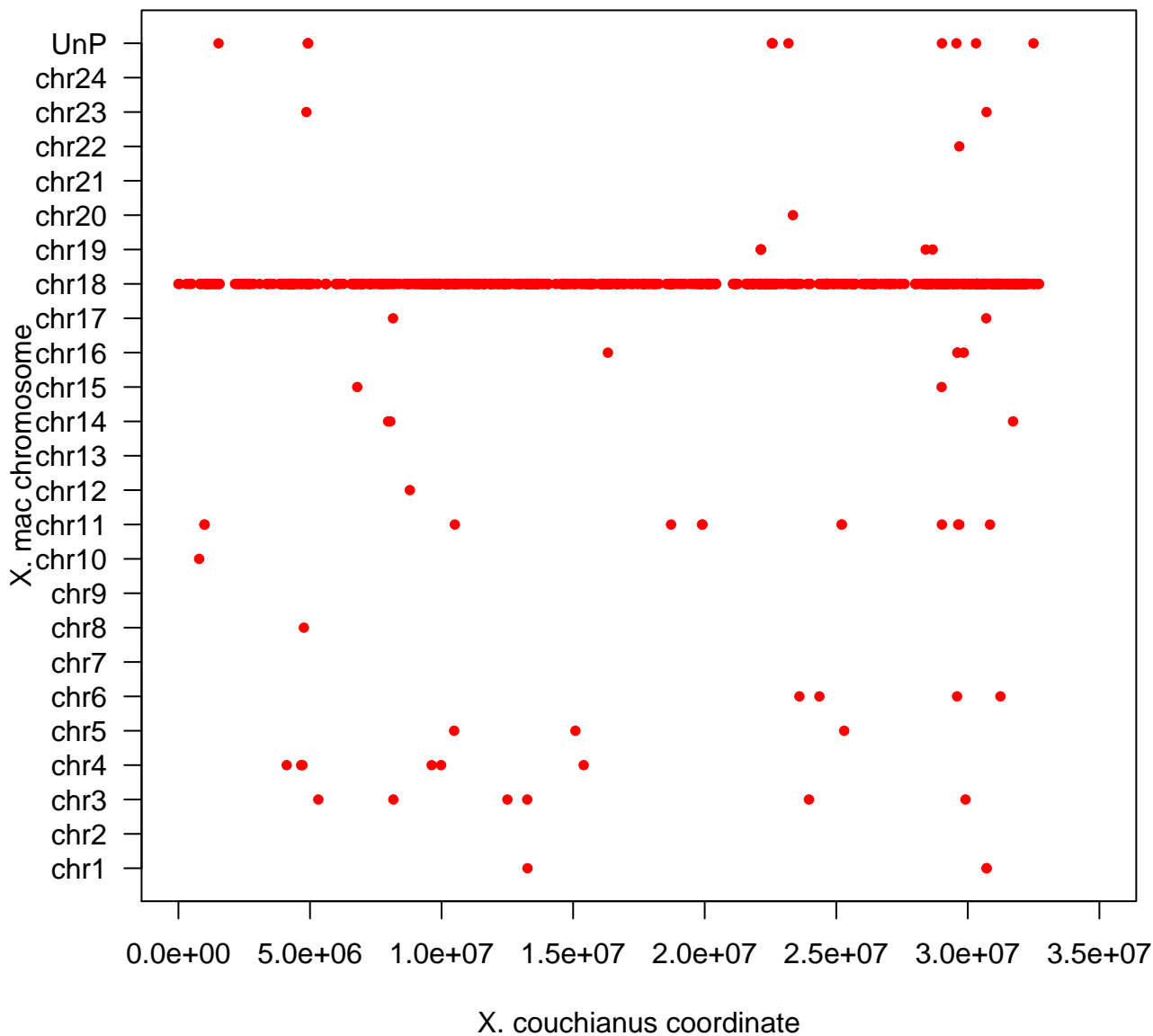

# LG19

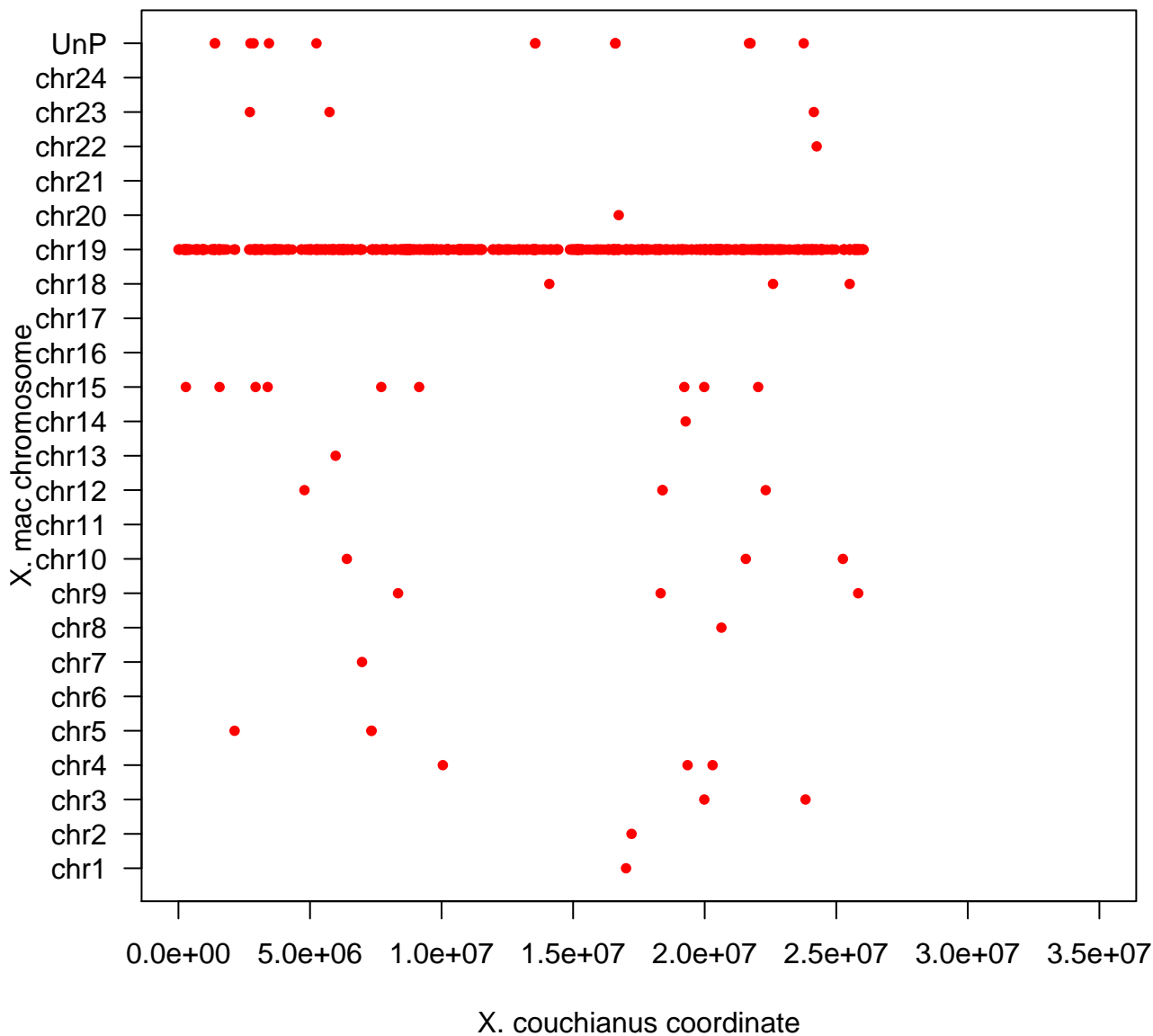

# LG2

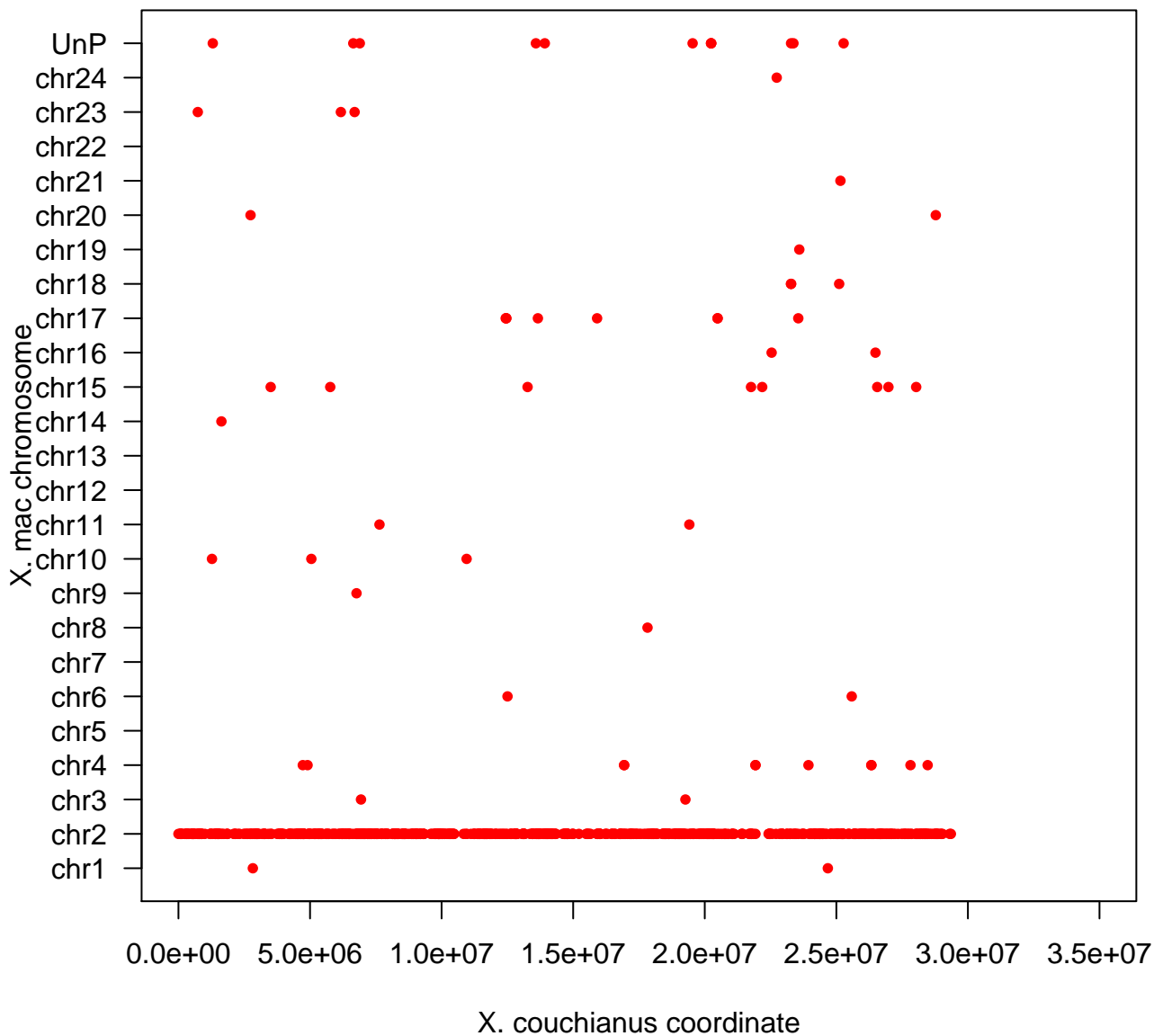

# LG20

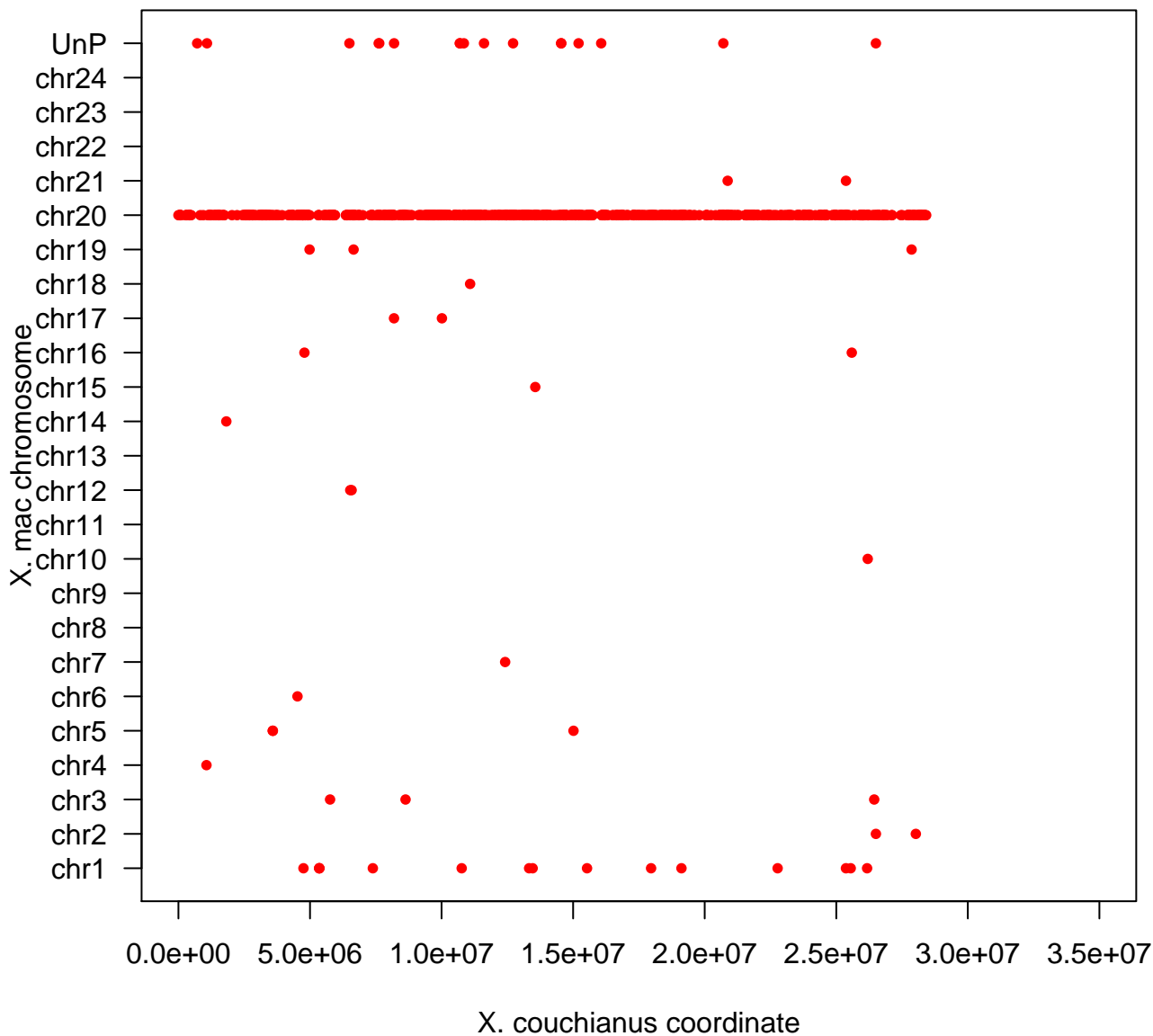

# LG21

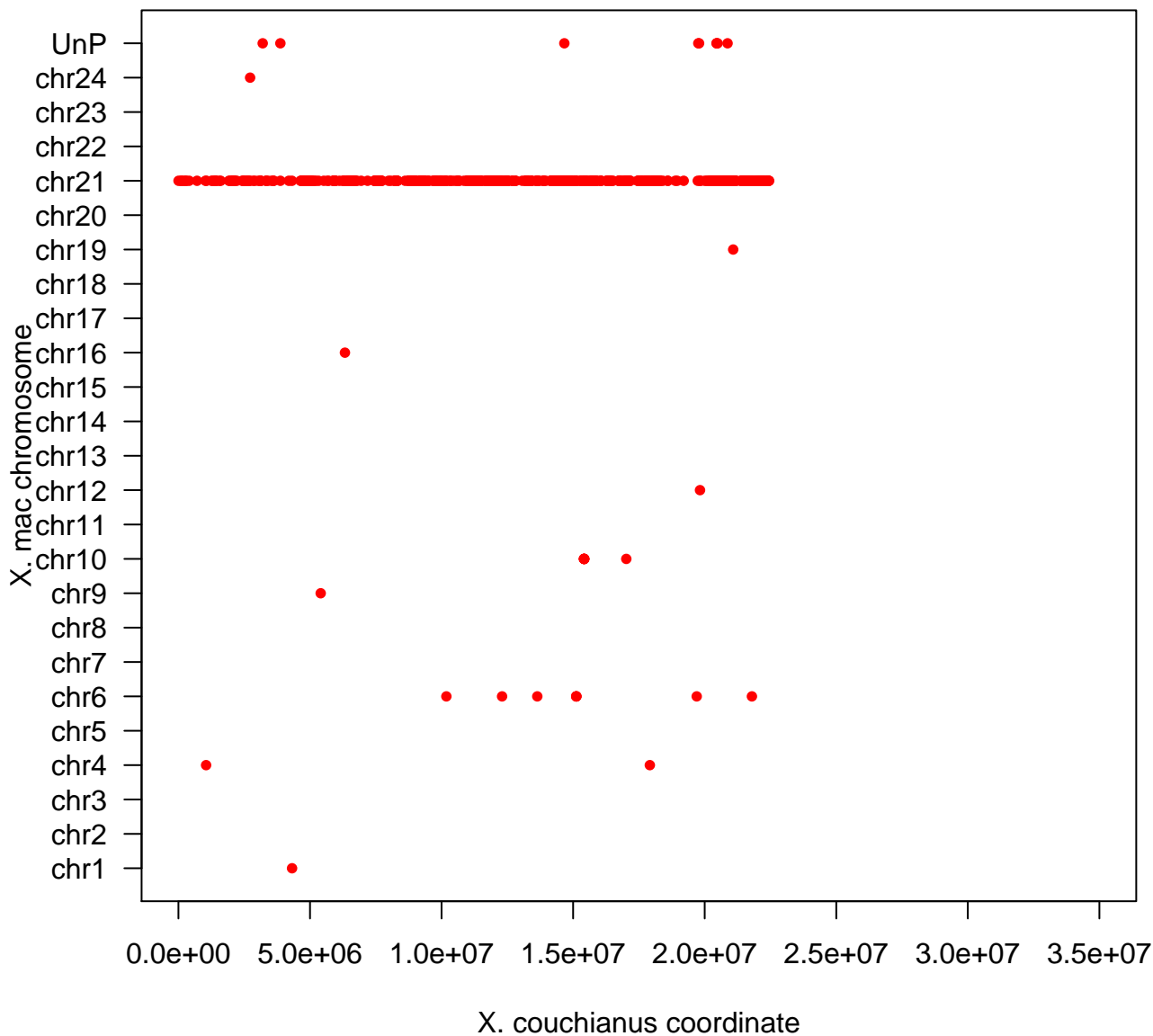

# LG22

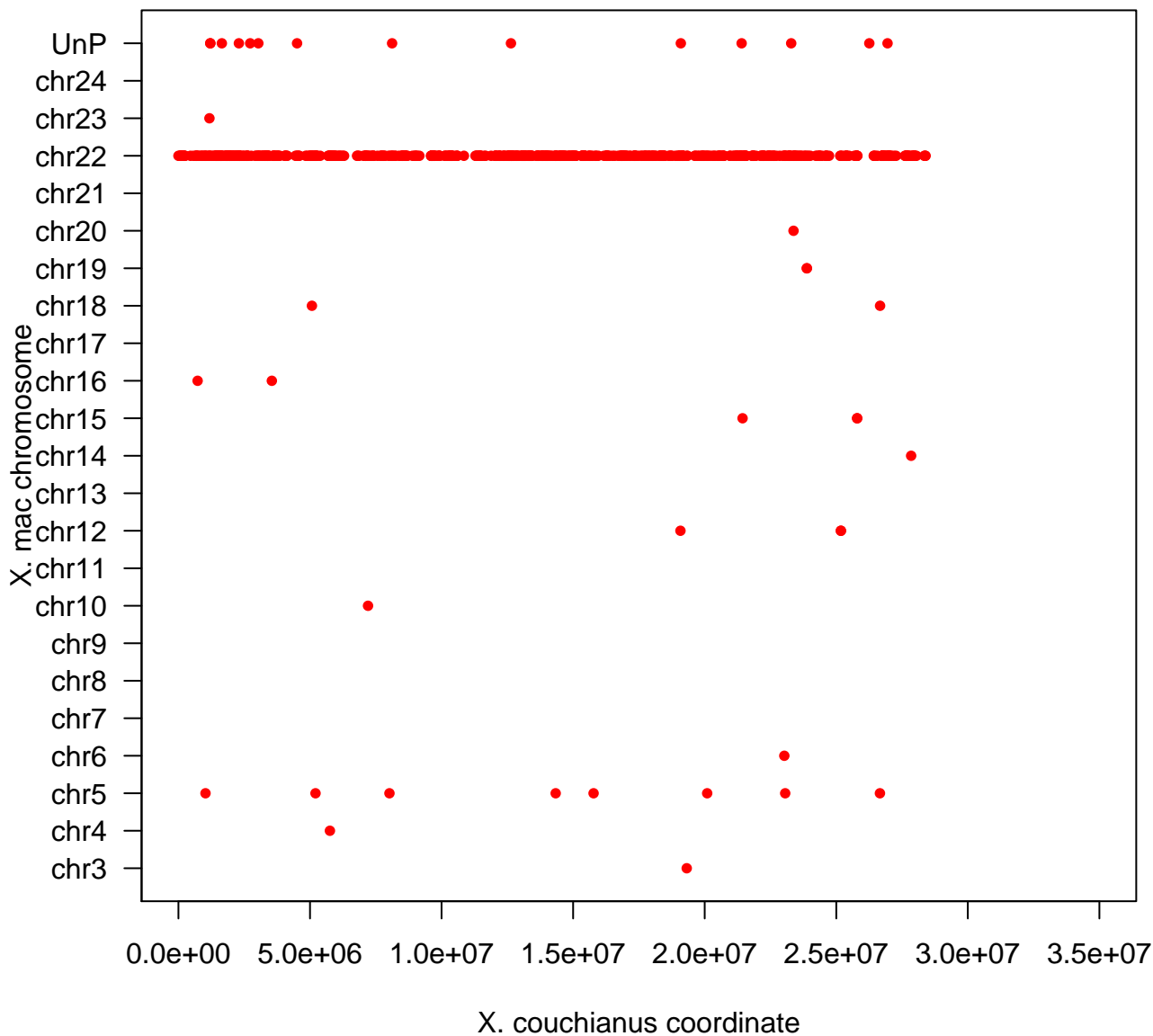

# LG23

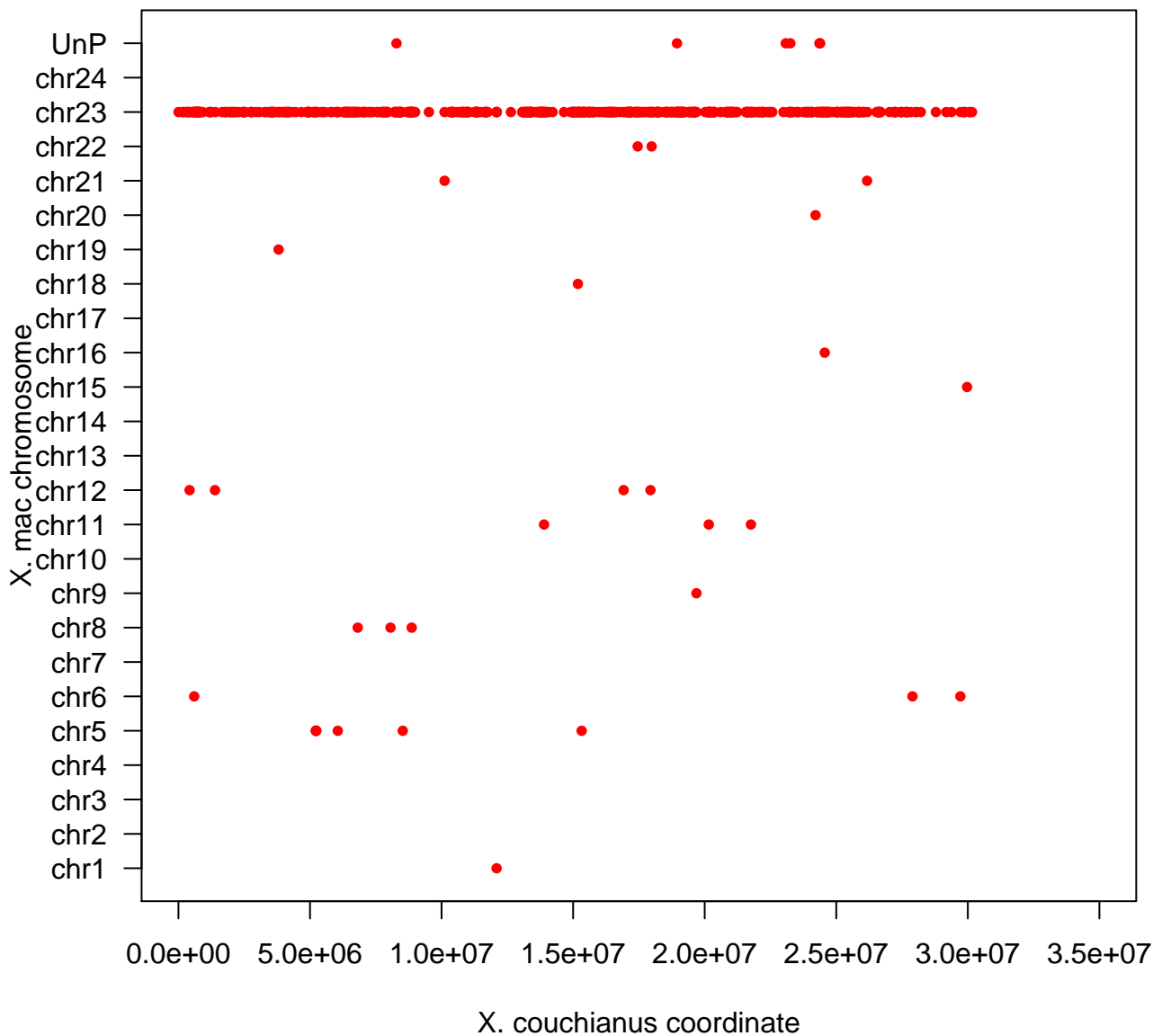

# LG24

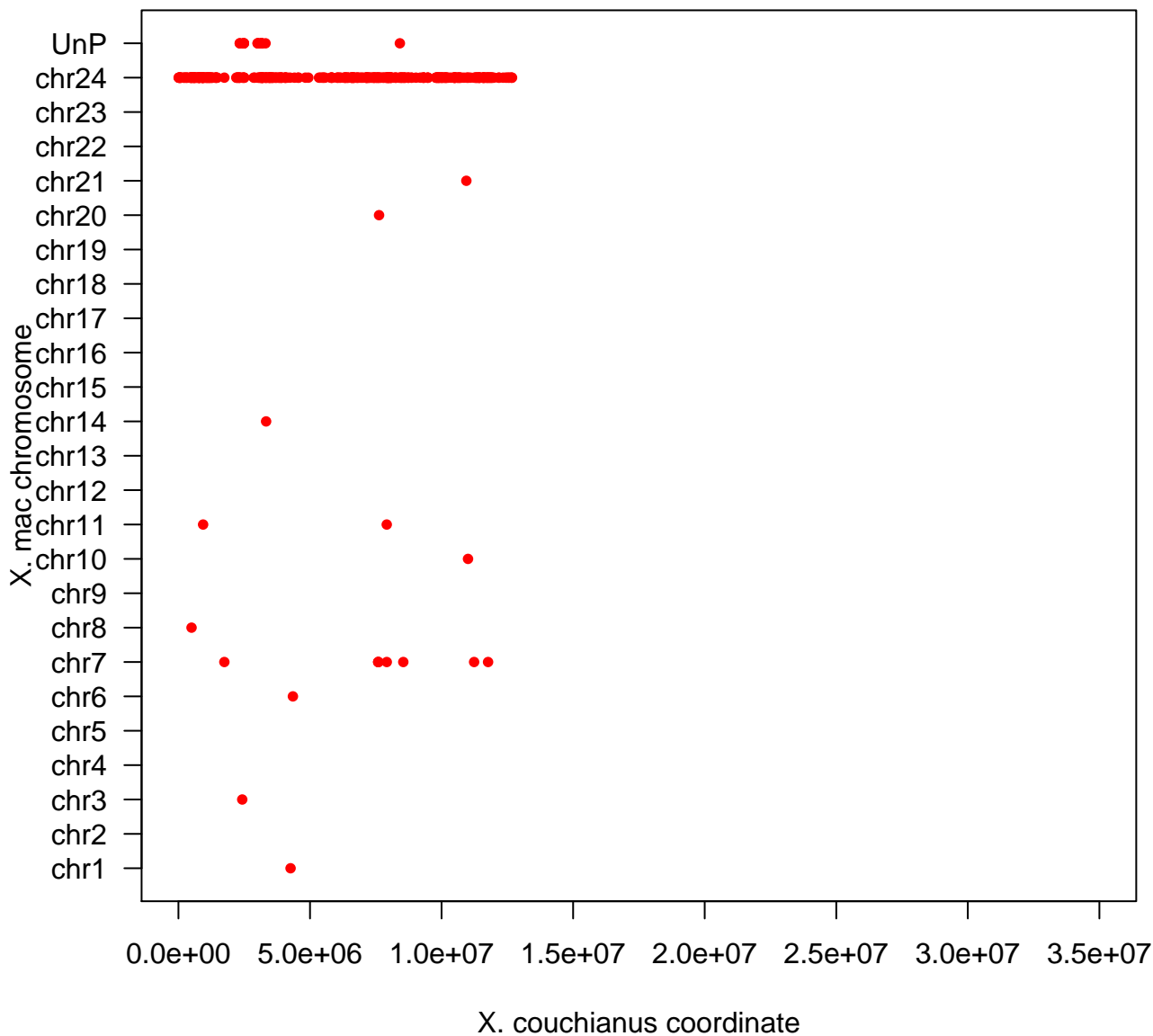

# LG3

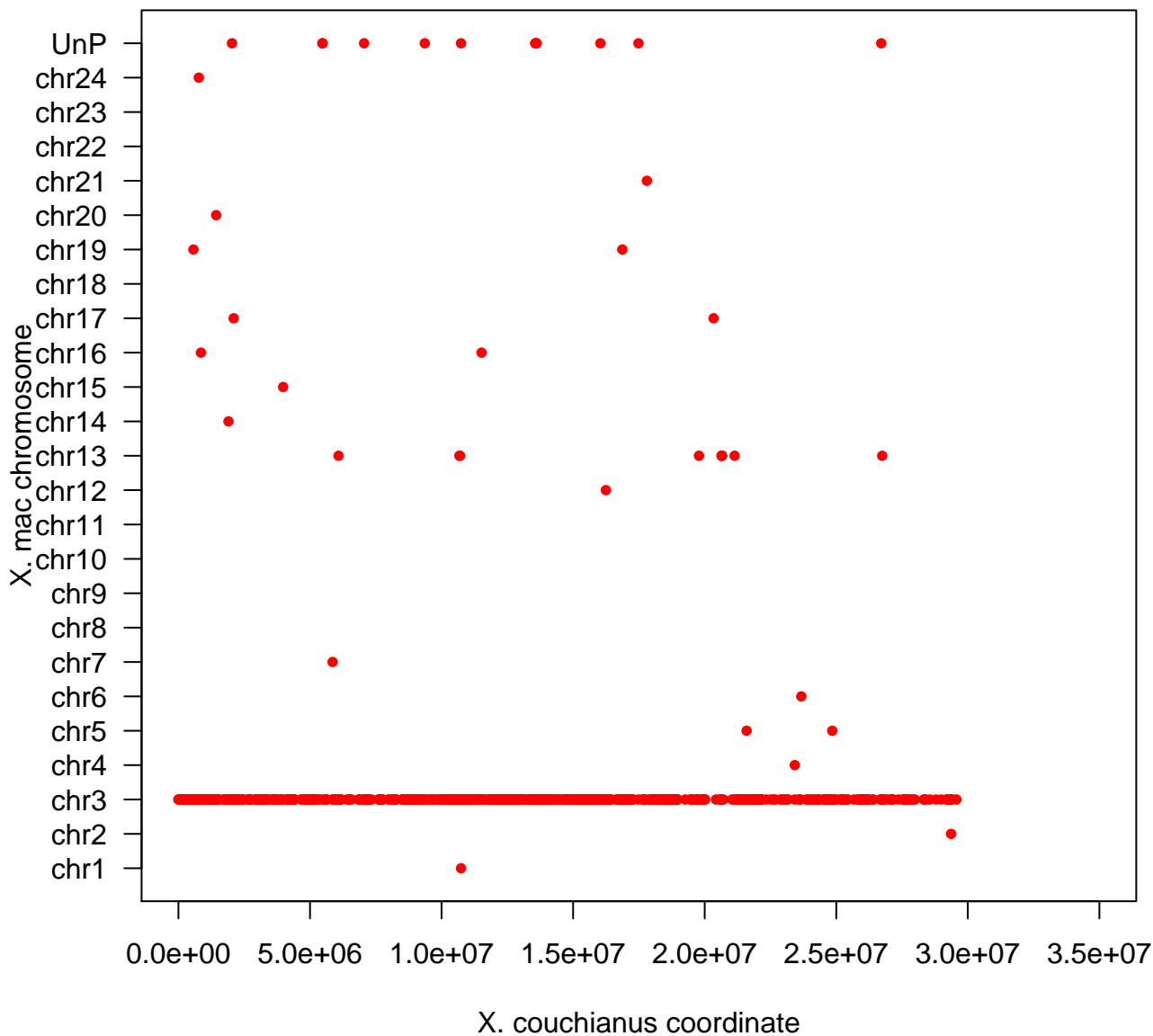

# LG4

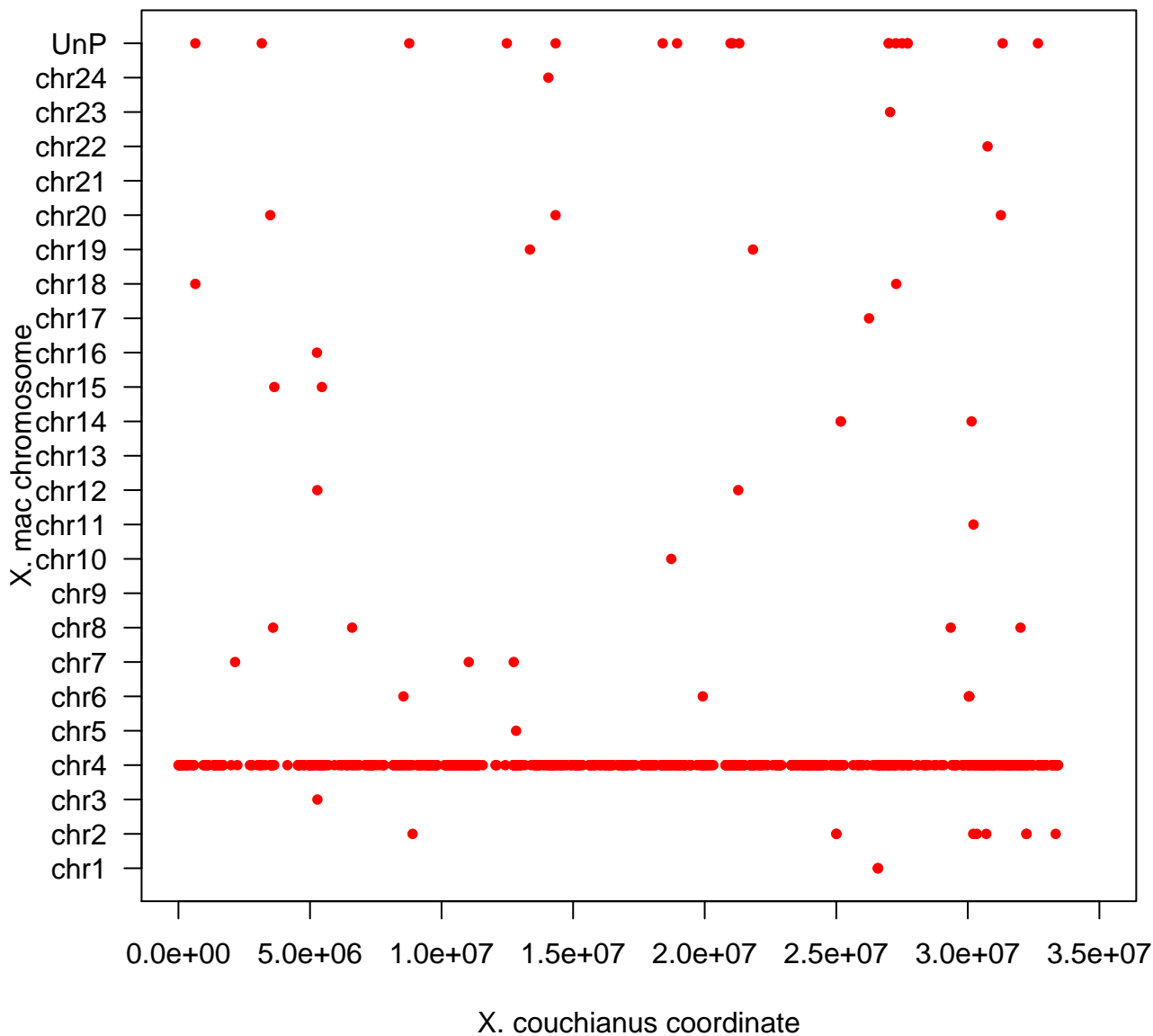

# LG5

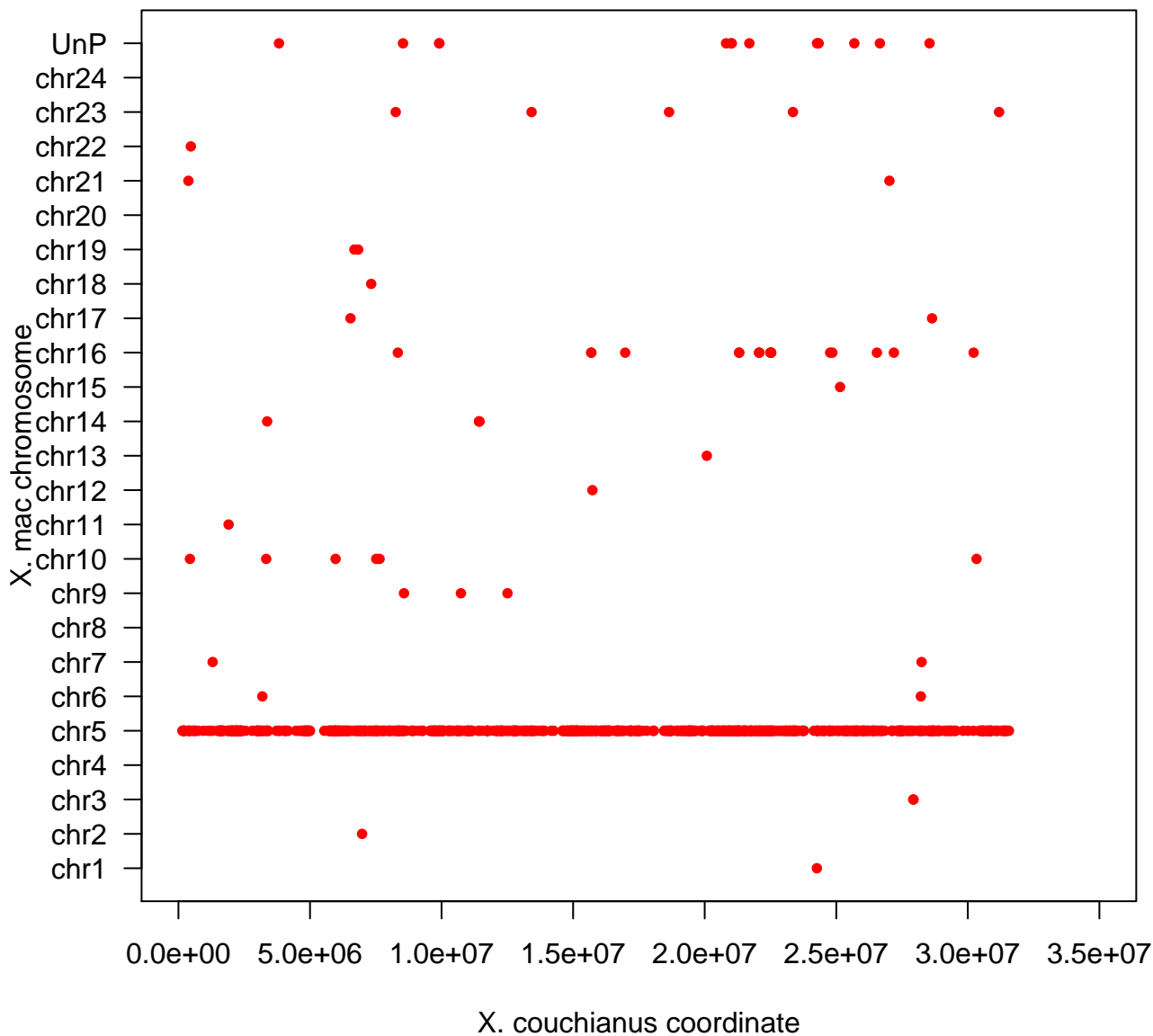

# LG6

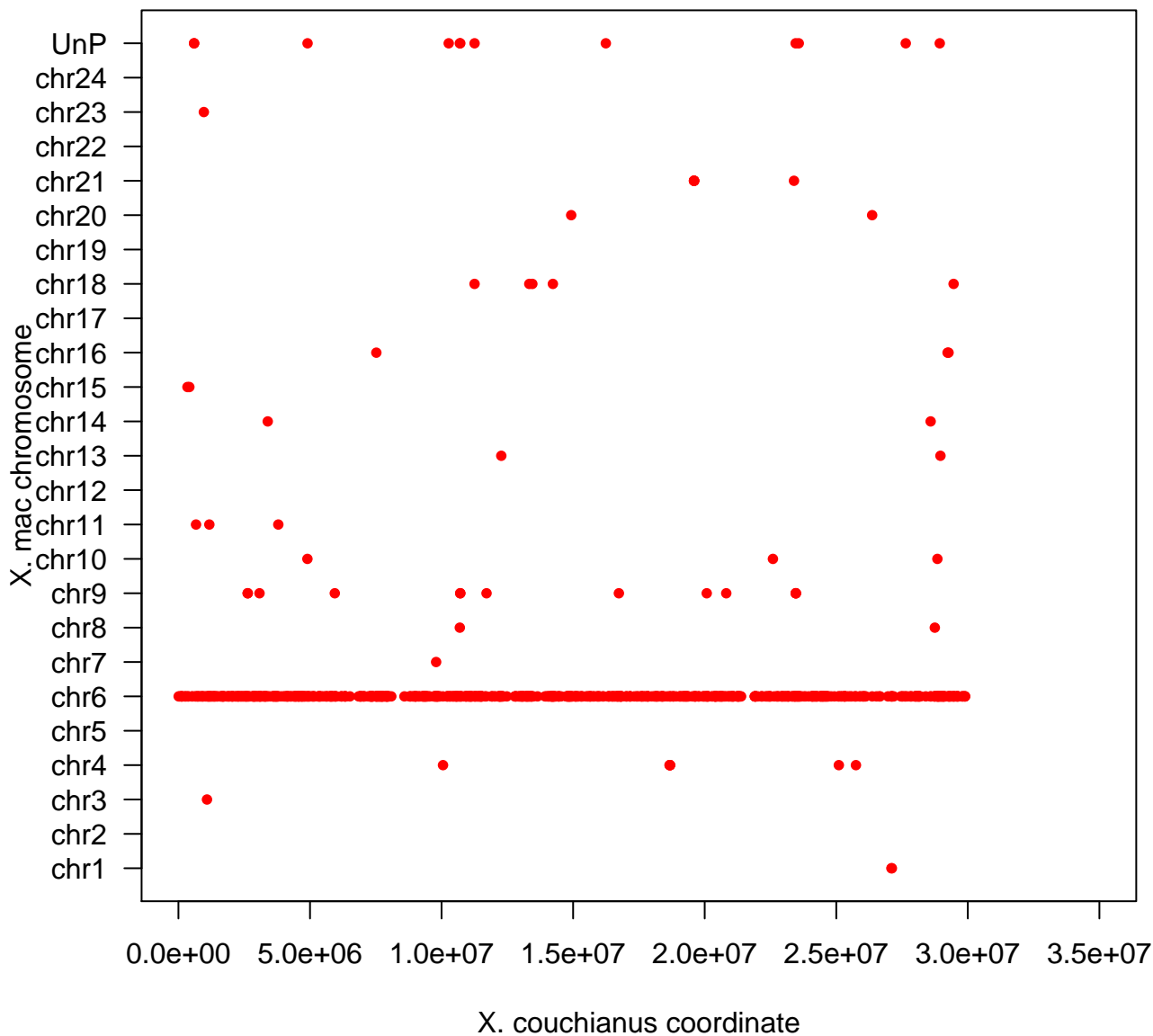

# LG7

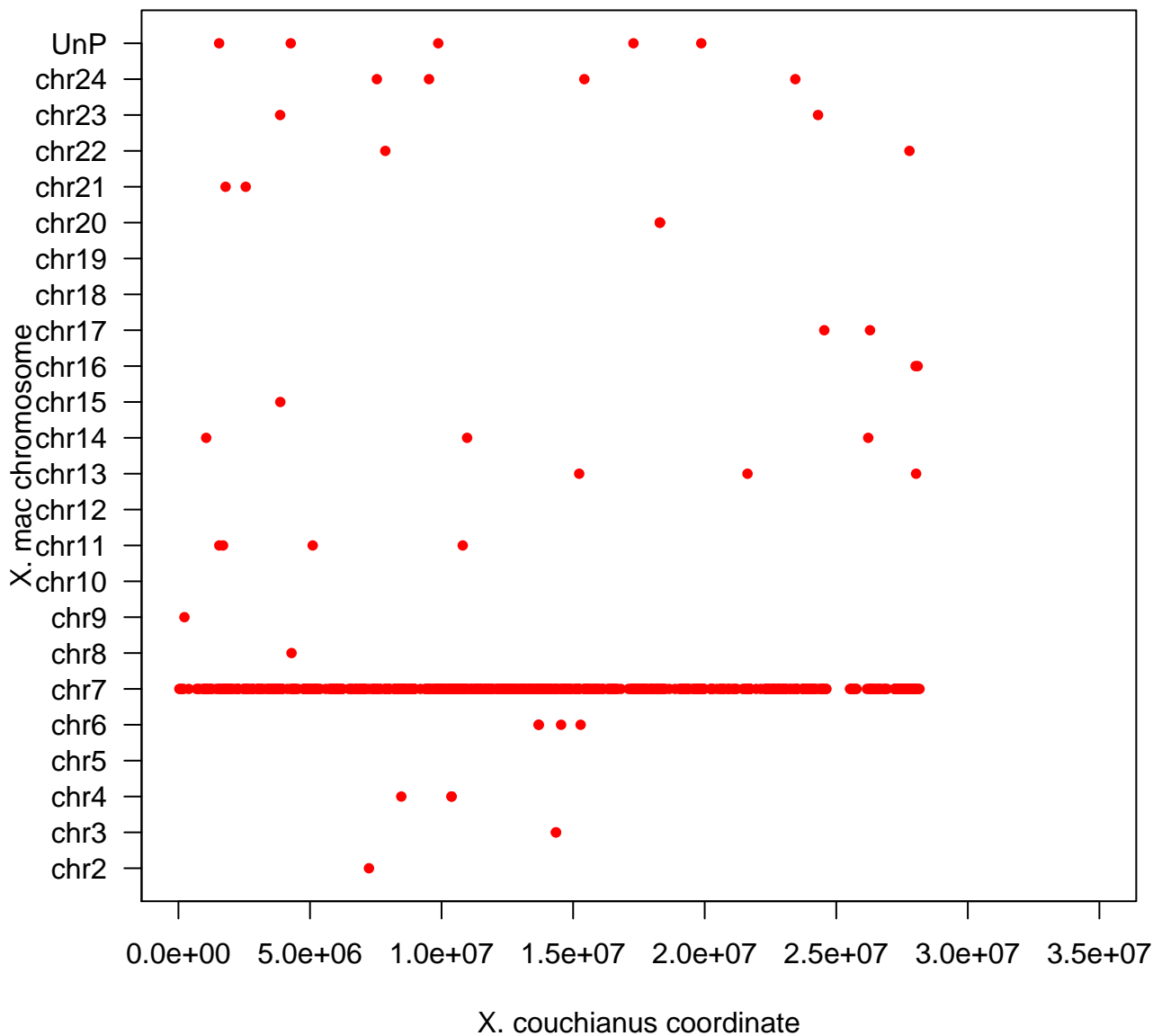

# LG8

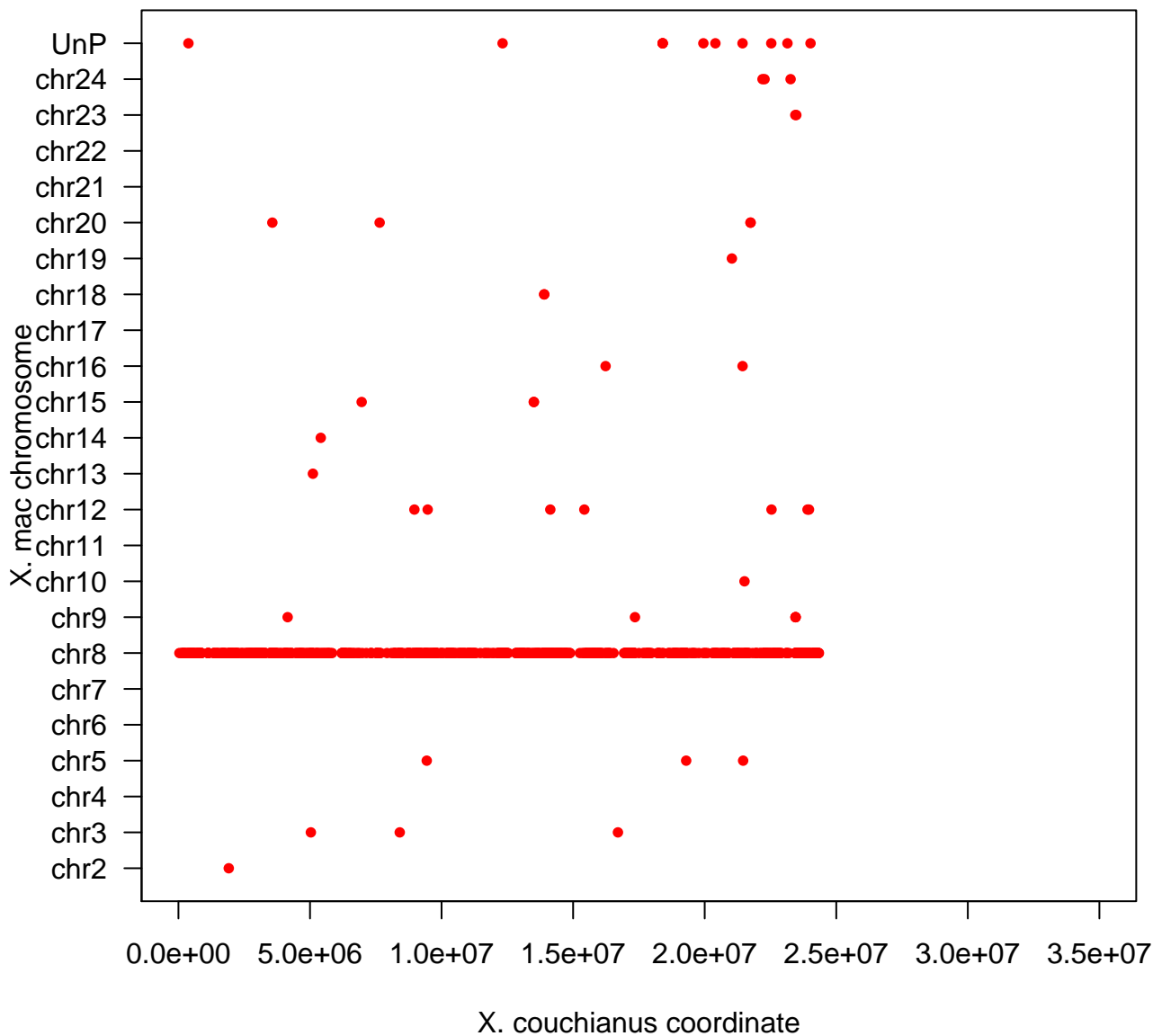

# LG9

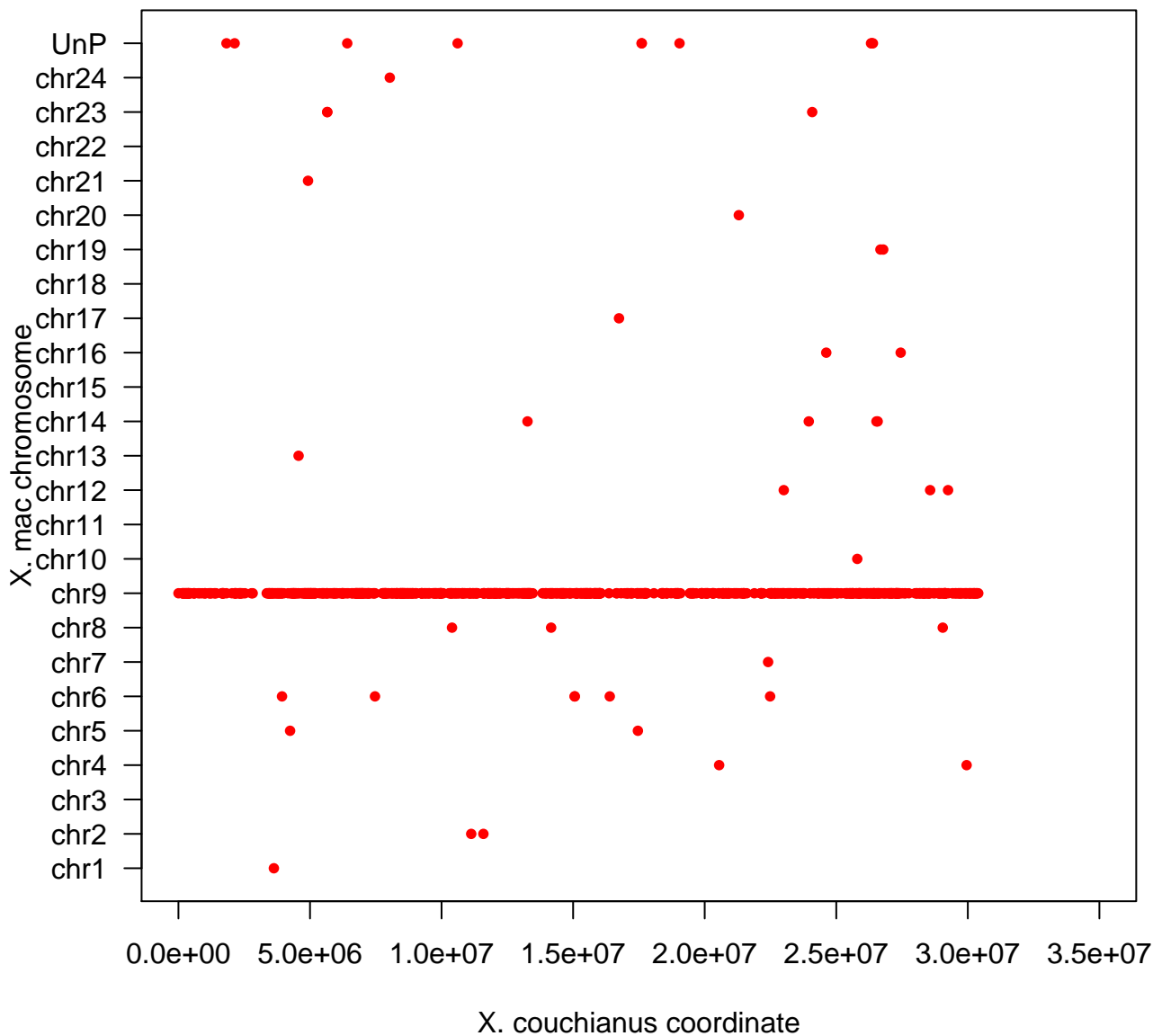

# unplaced

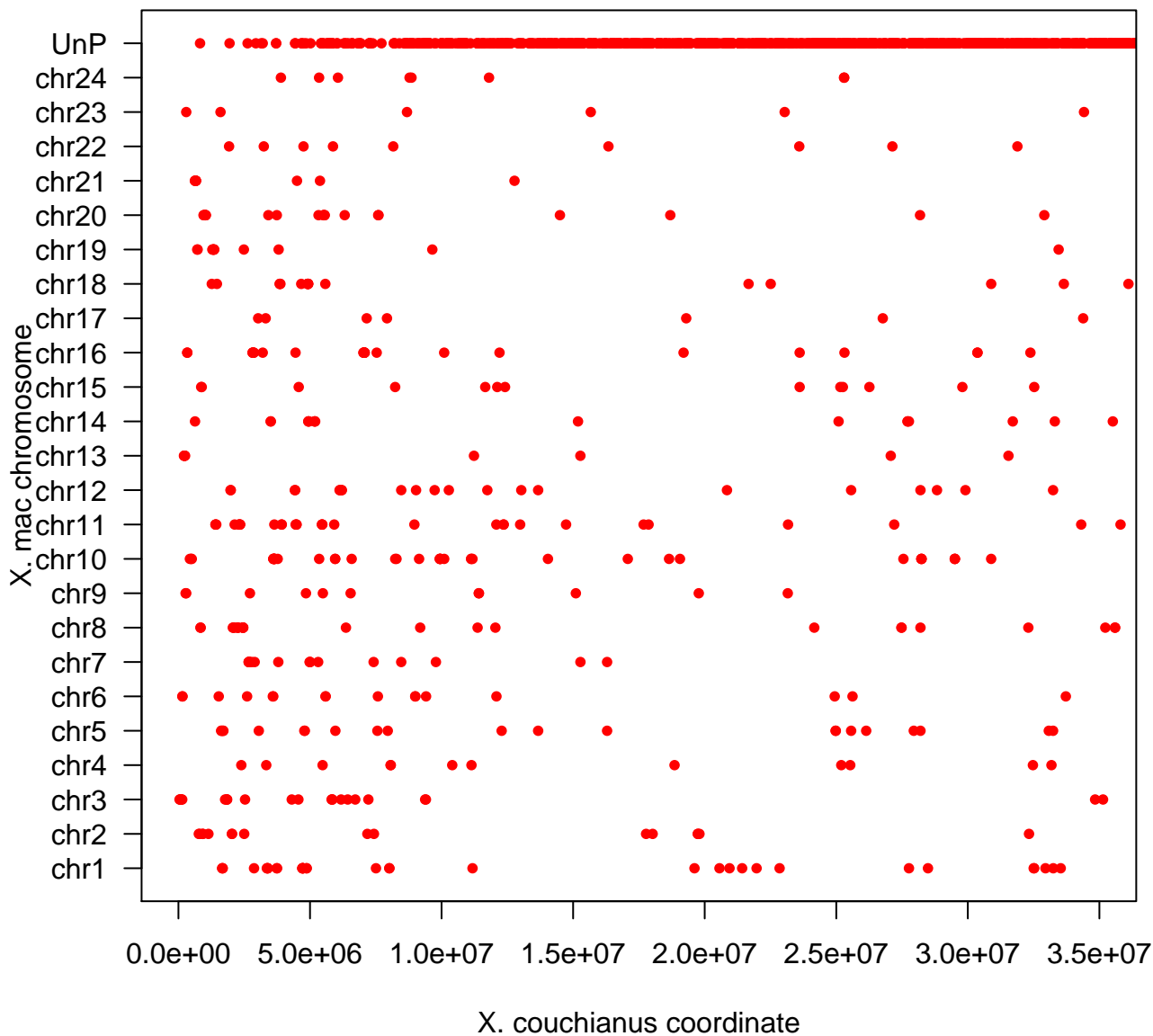

Supplement: Additional file 6: Figure S2. — Dot plots of location of one-to-one orthologues in the 24 chromosomes of X. couchianus and X. maculatus. (PDF 110 kb) [file 12864_2015_2361_MOESM6_ESM.pdf]

# LG1

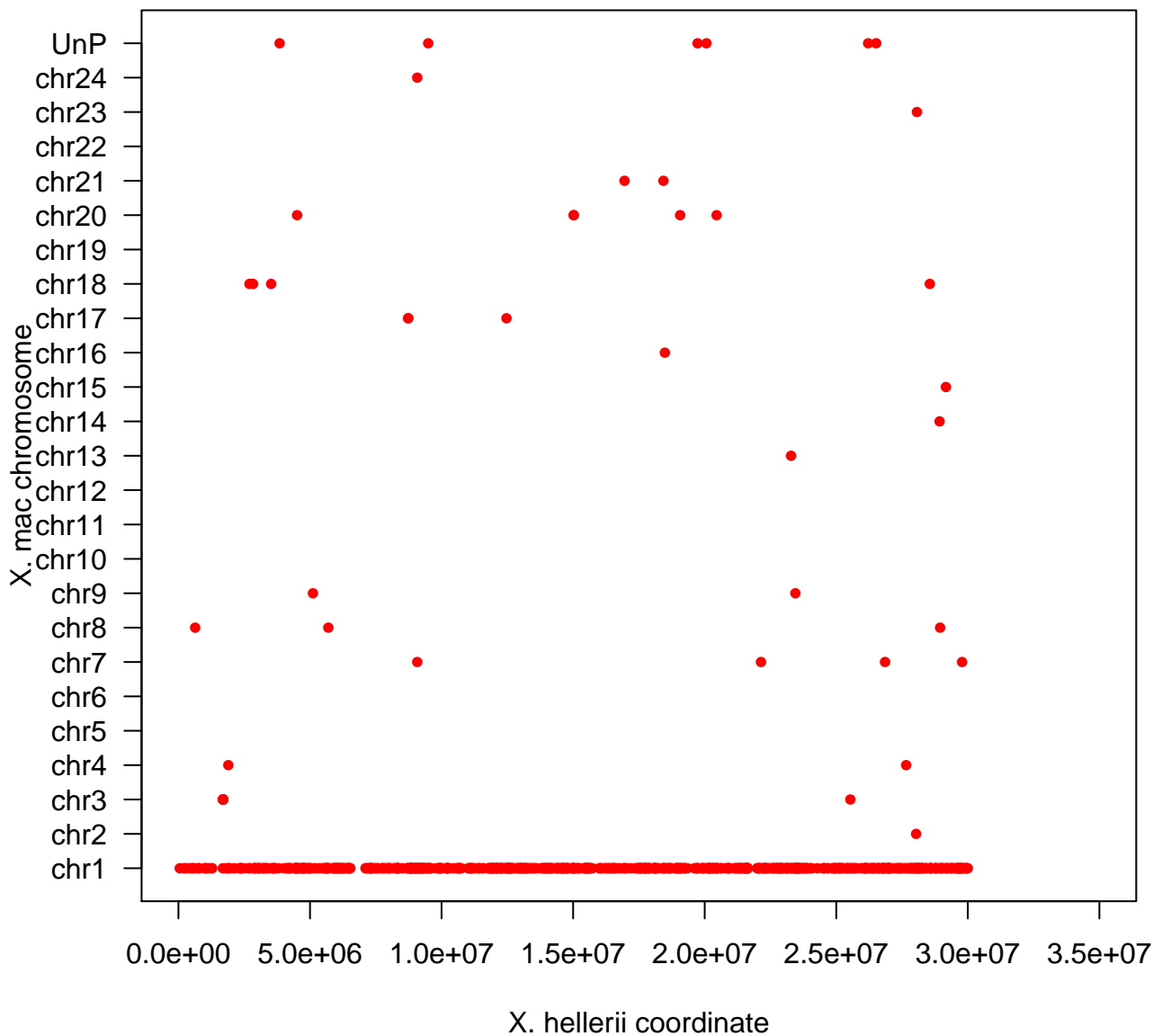

# LG10

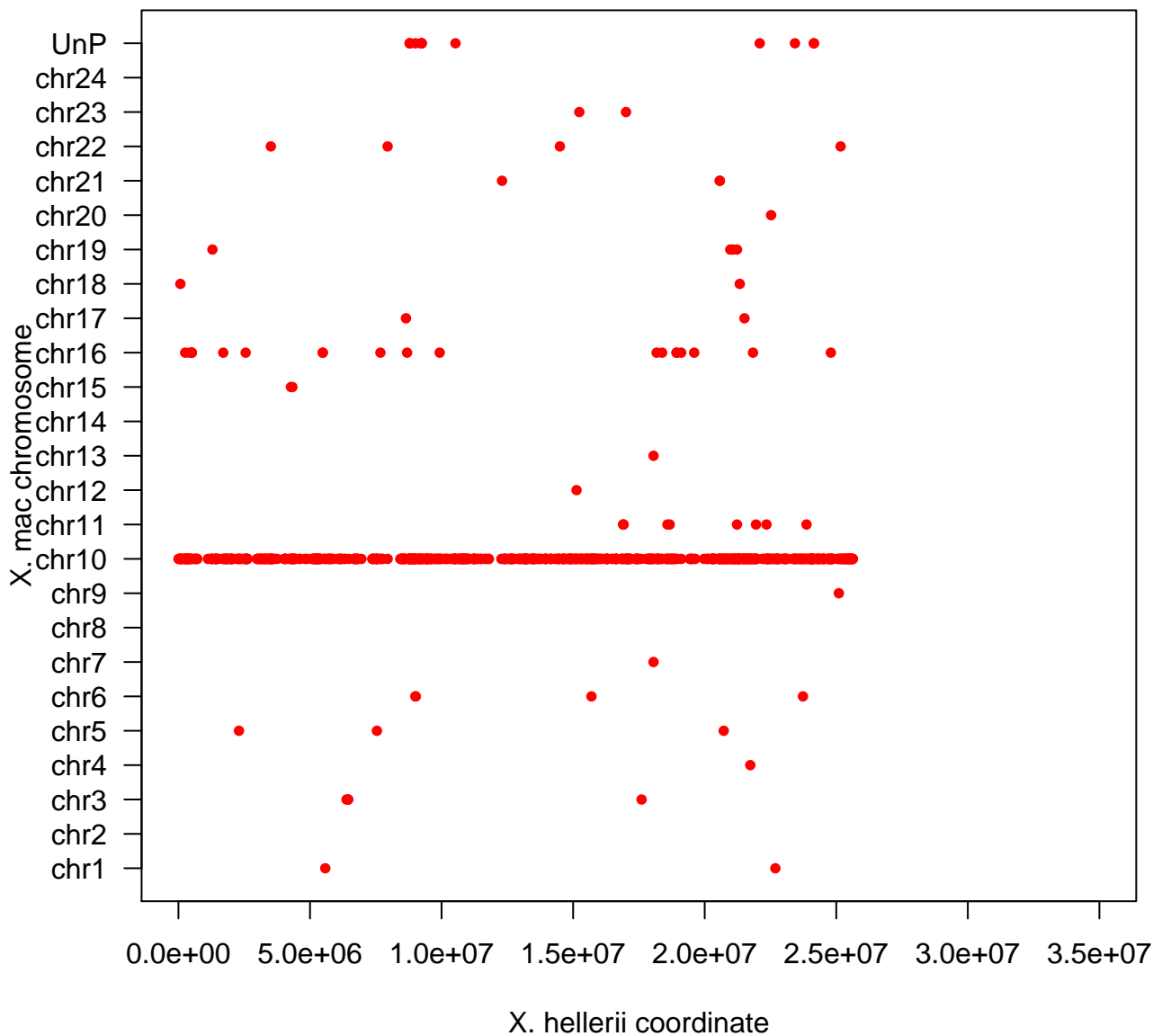

# LG11

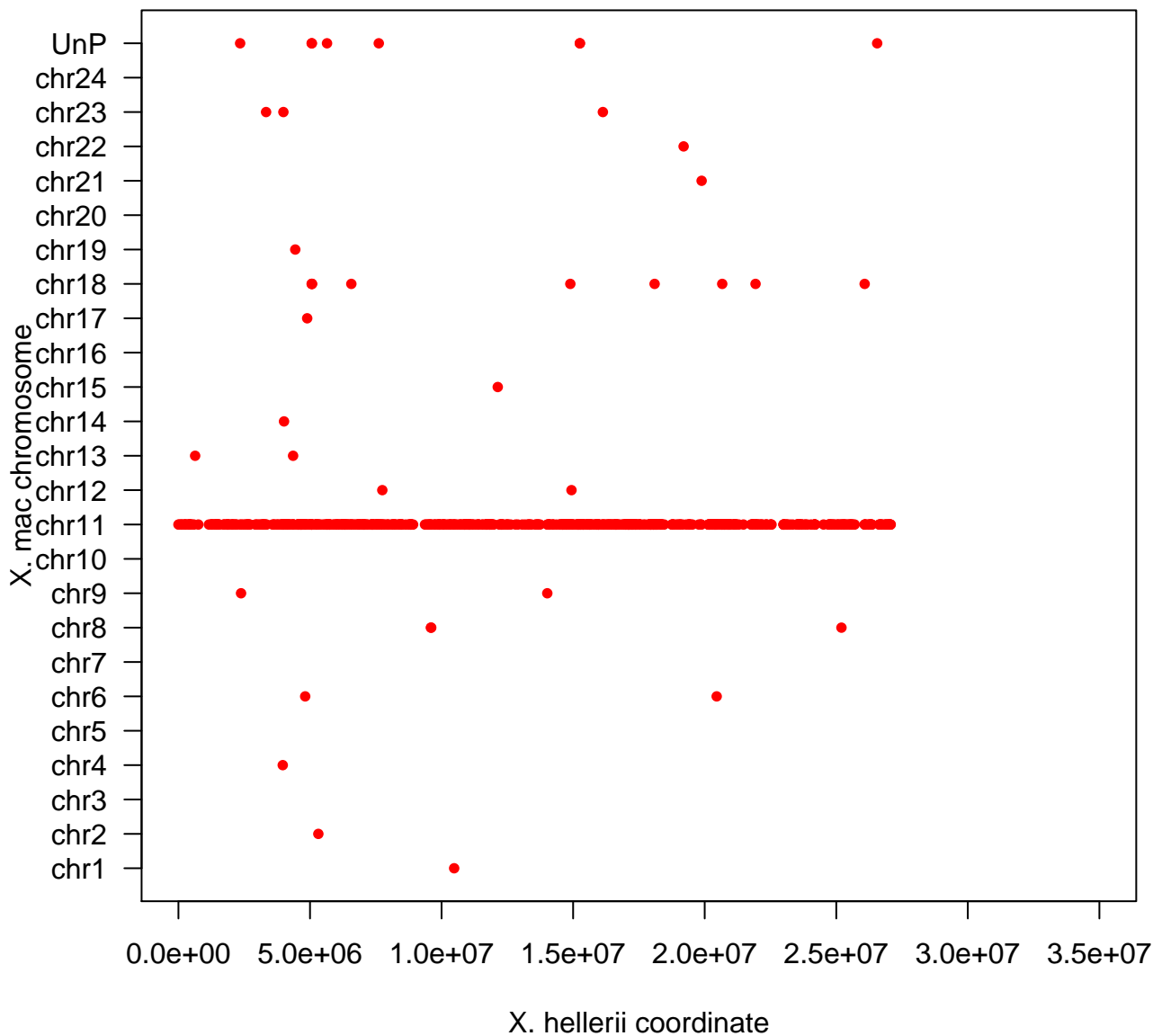

# LG12

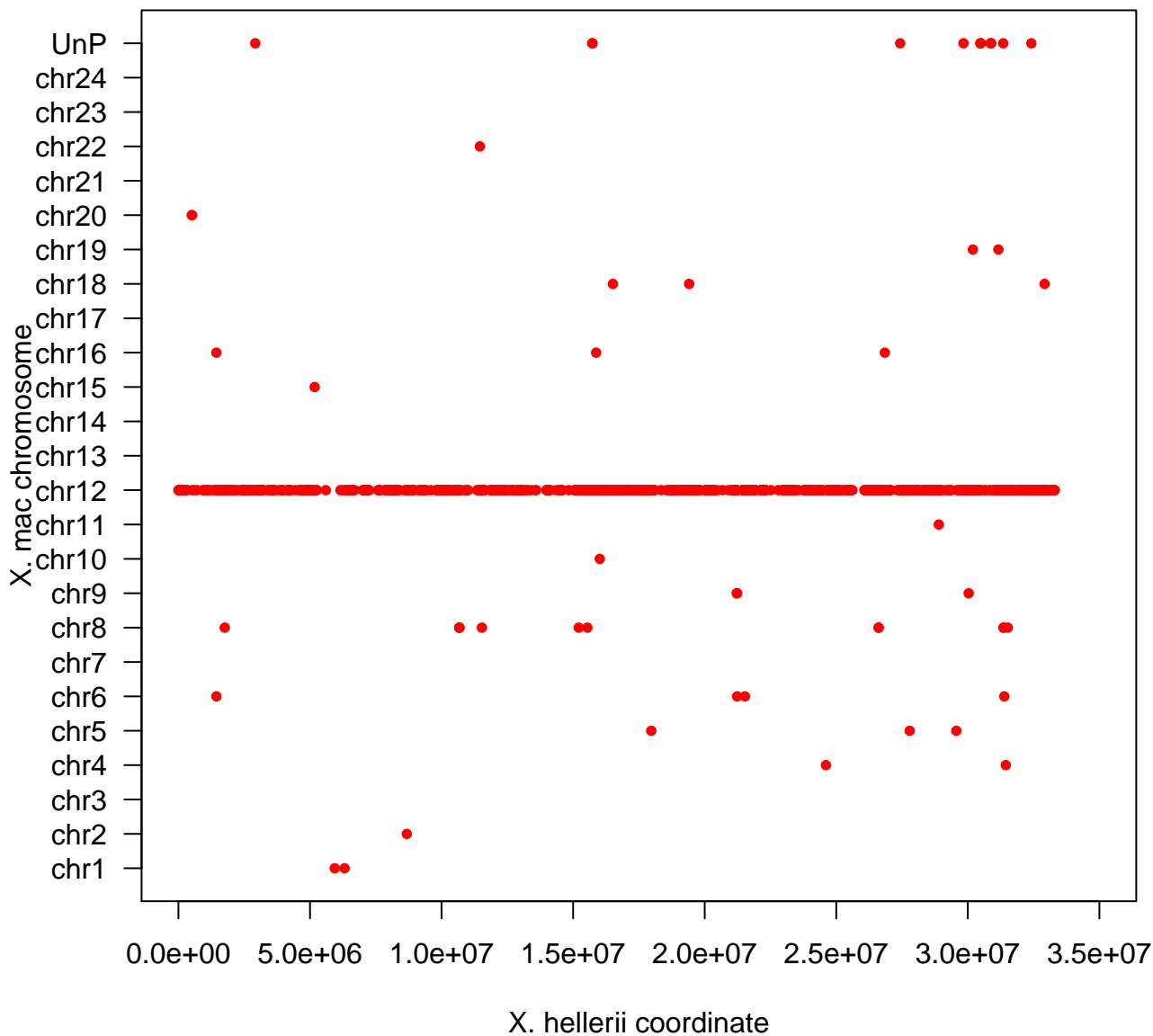

# LG13

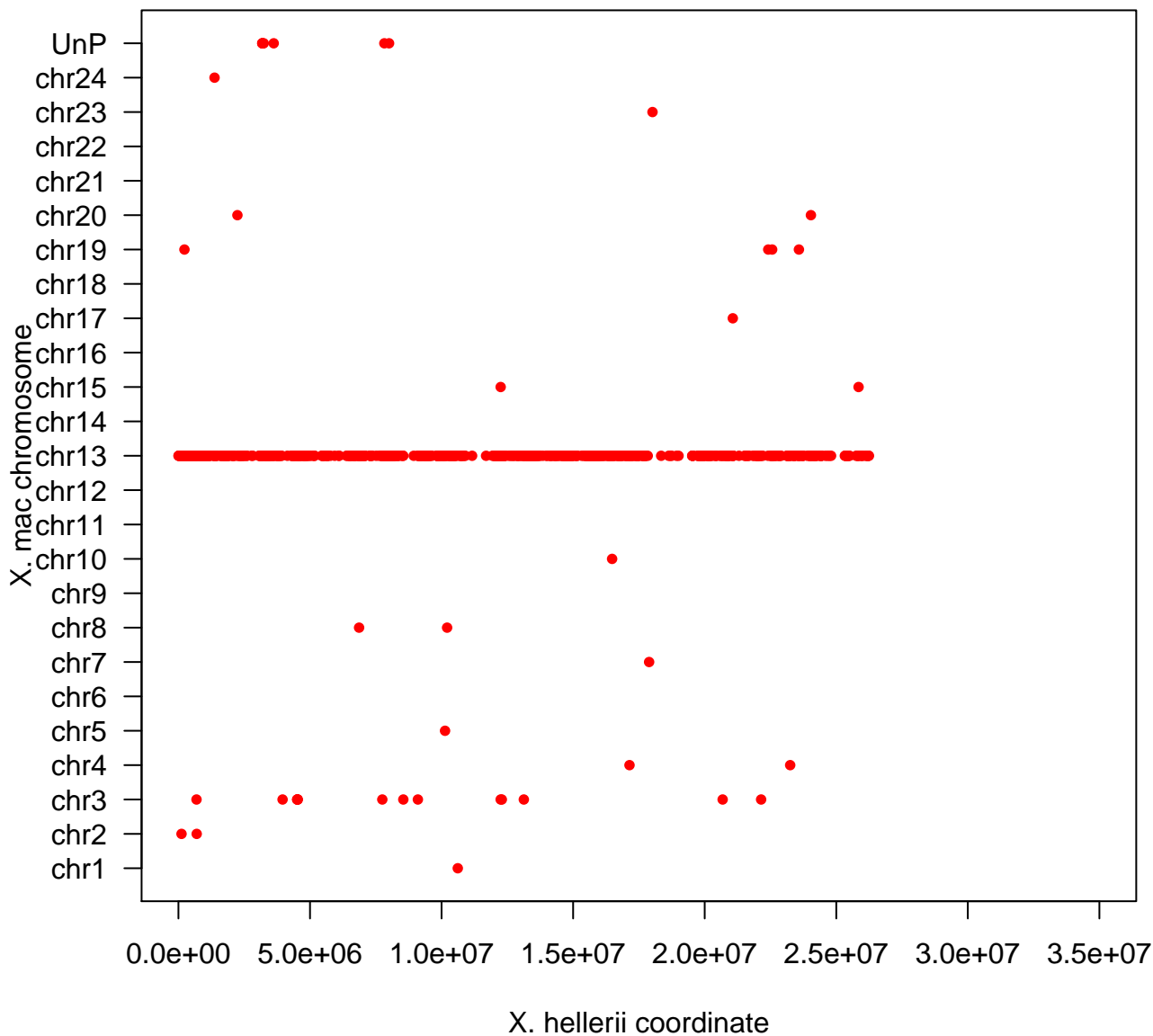

# LG14

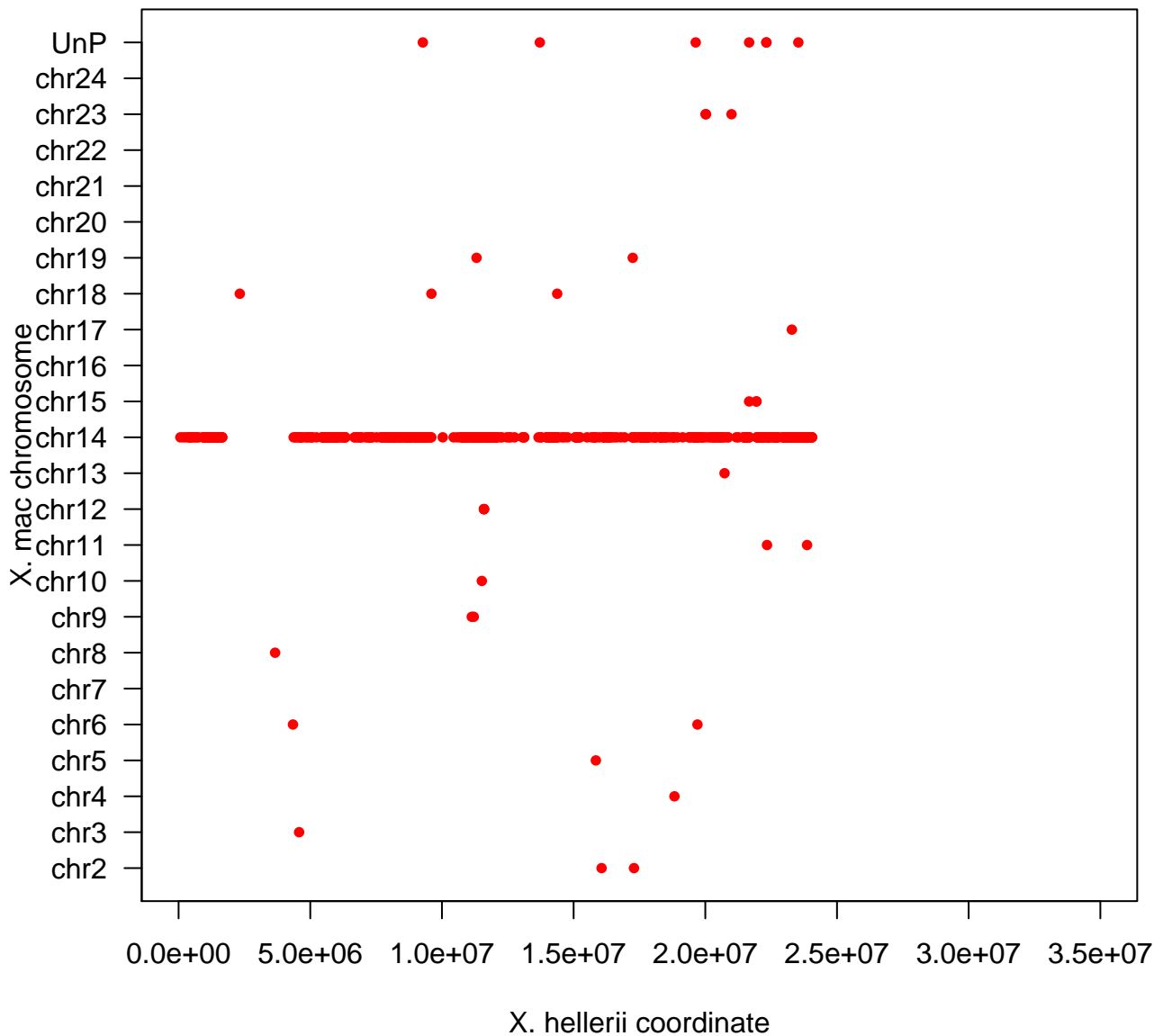

# LG15

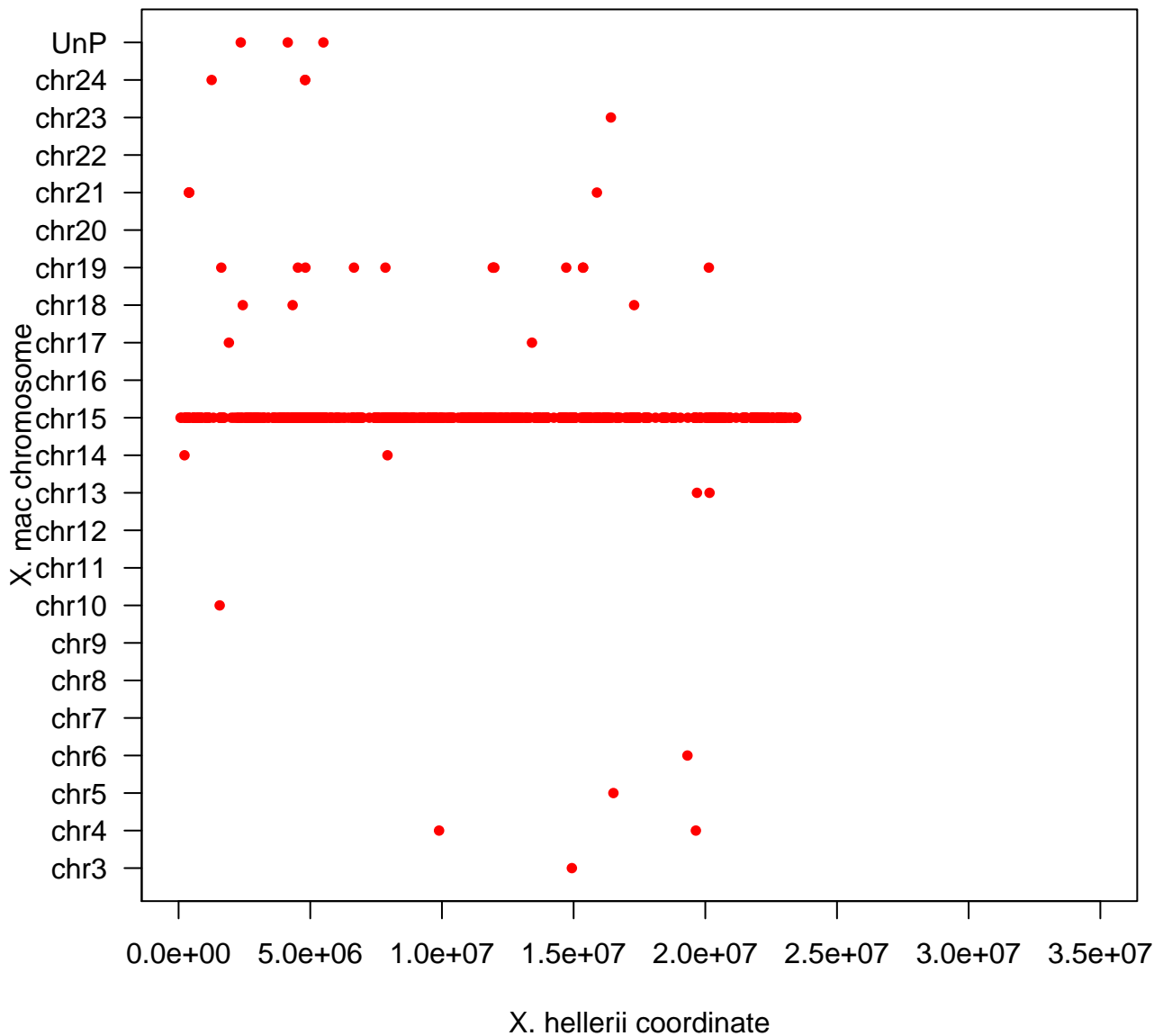

# LG16

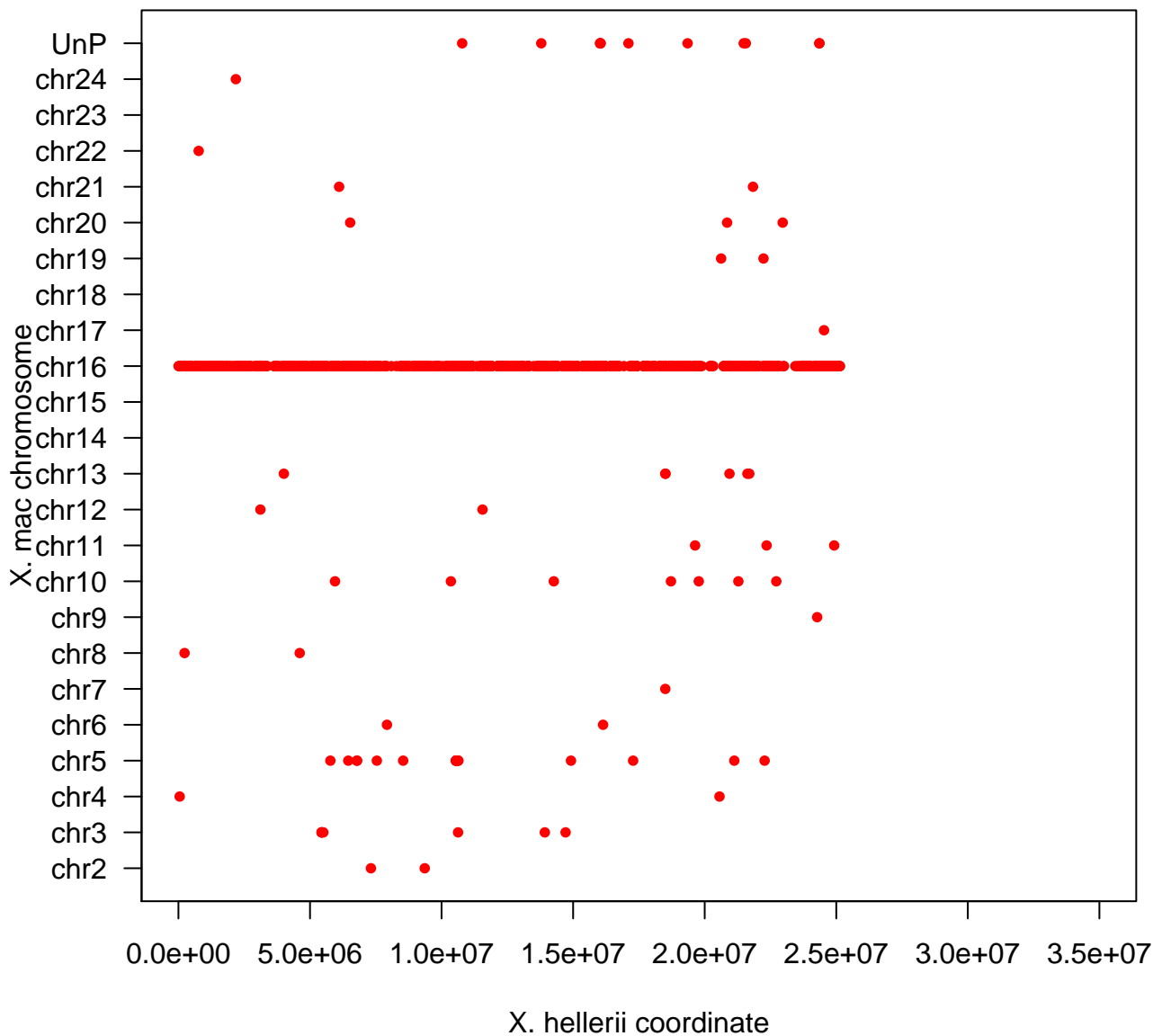

# LG17

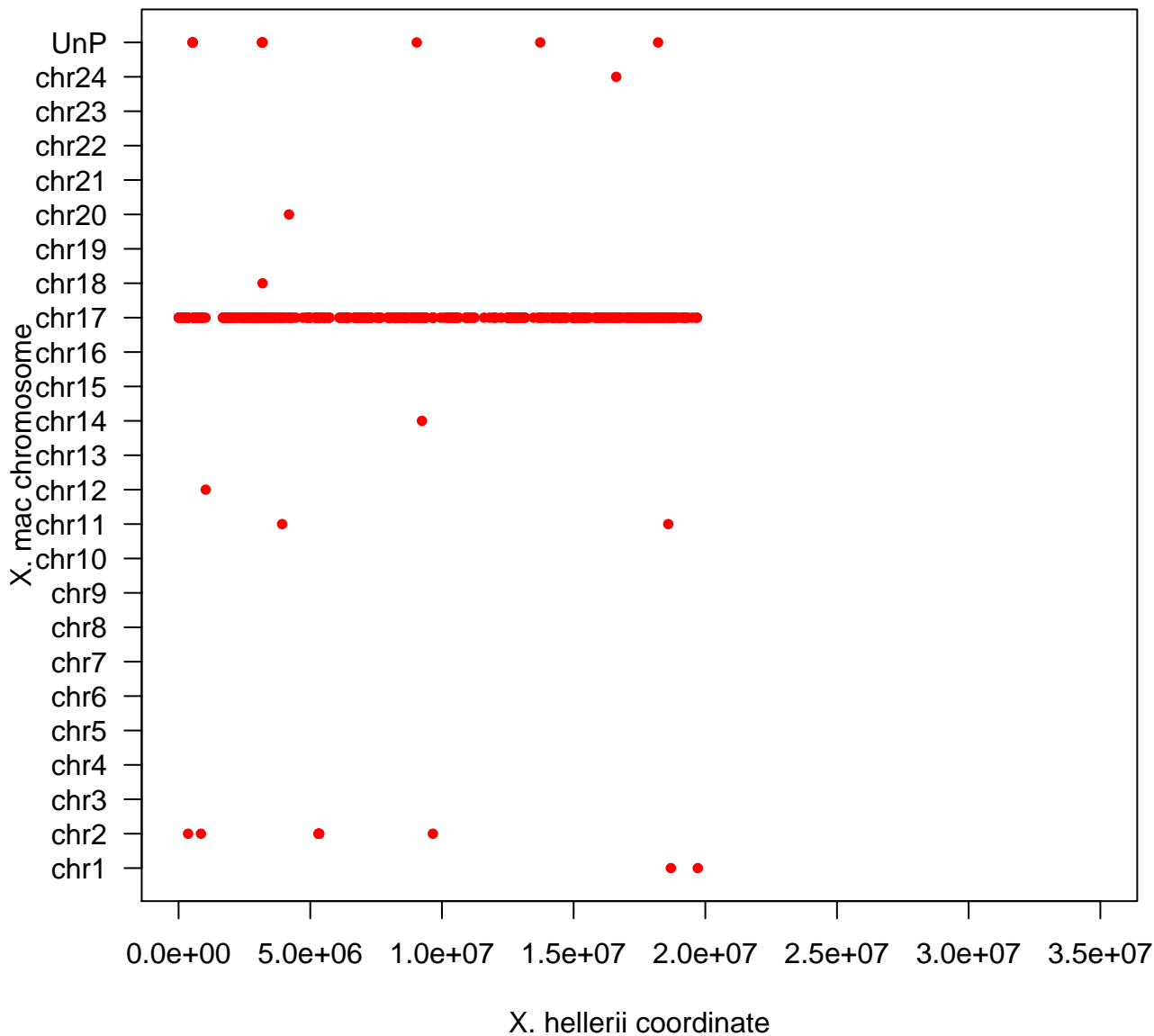

# LG18

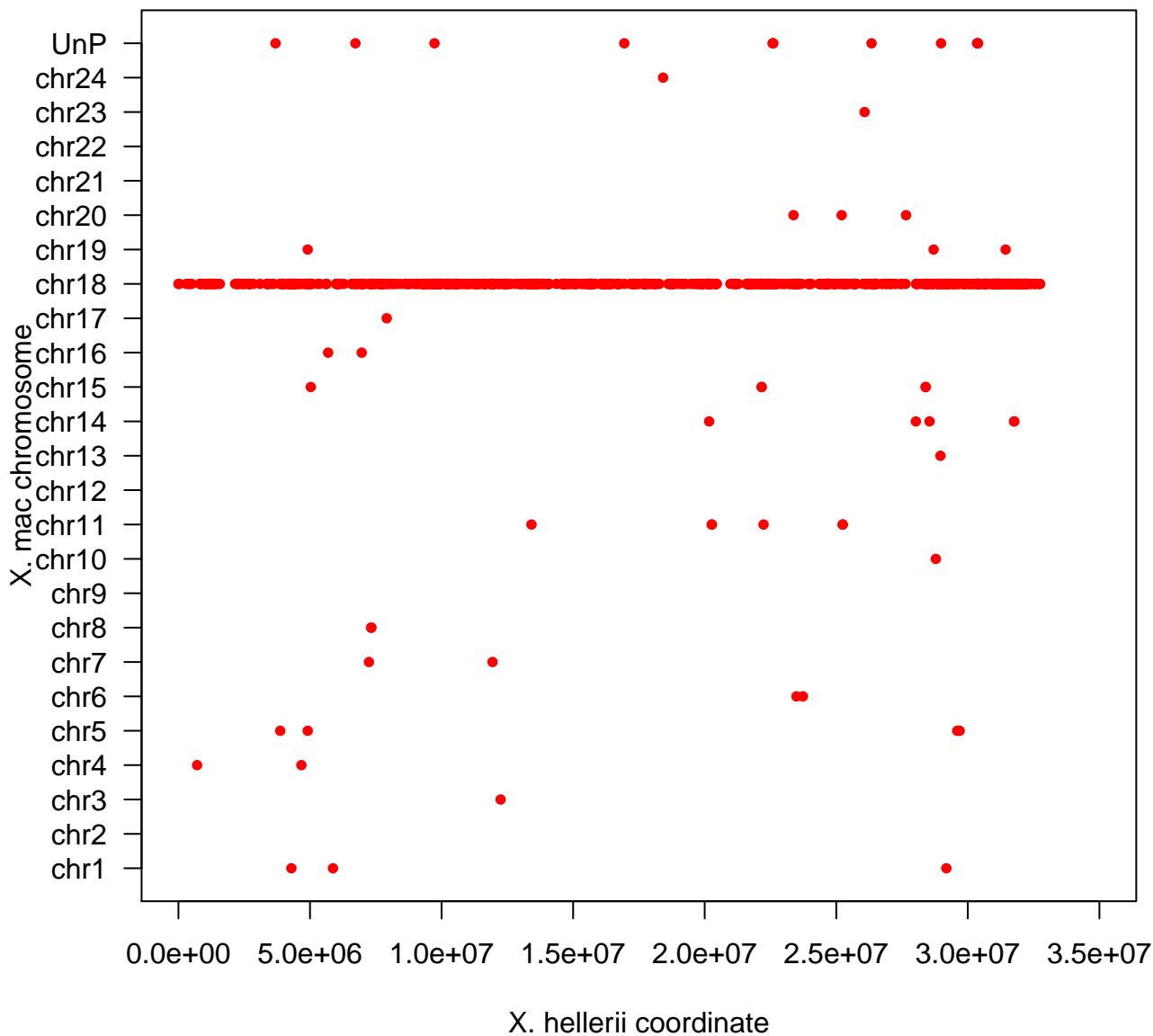

# LG19

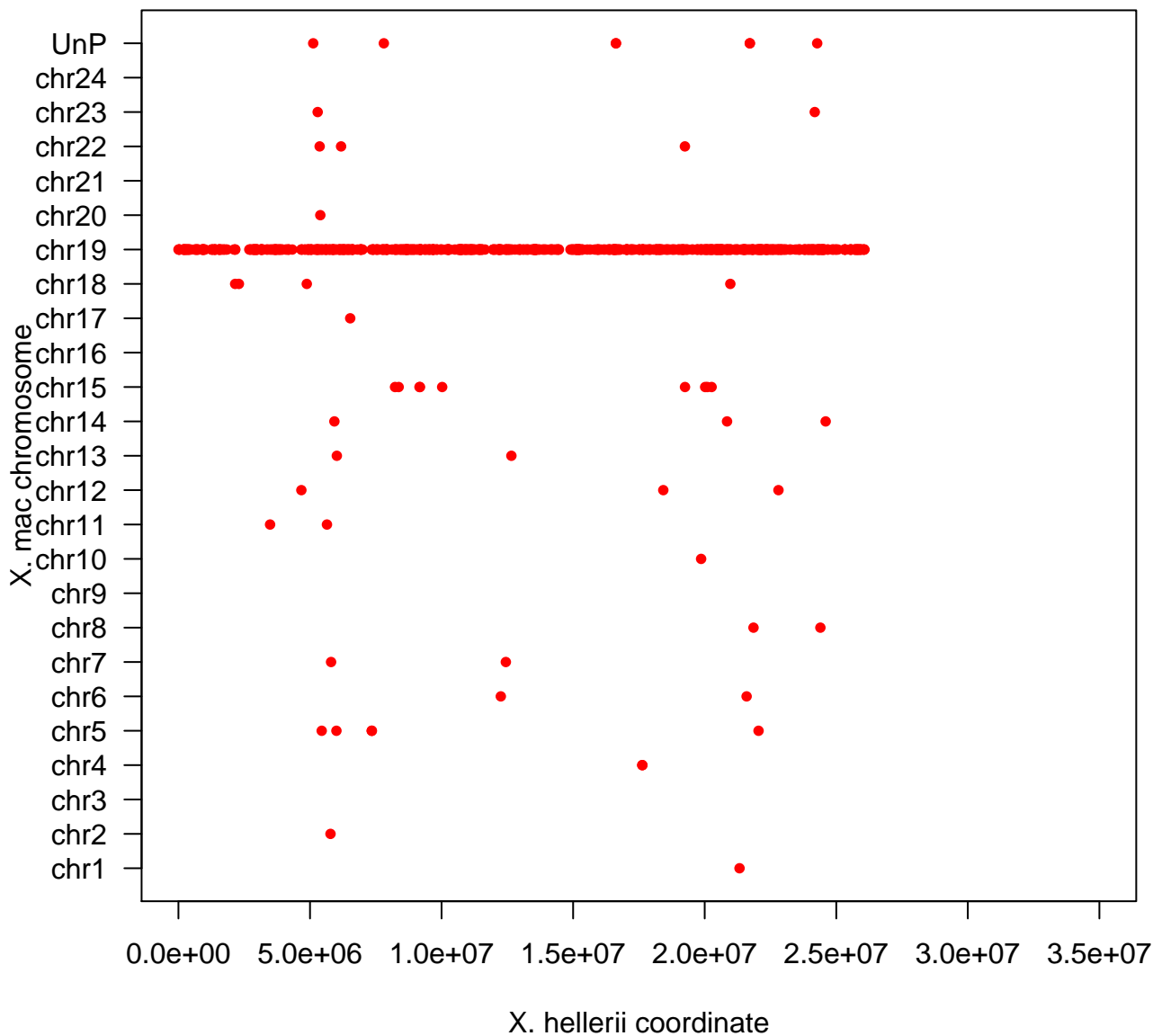

# LG2

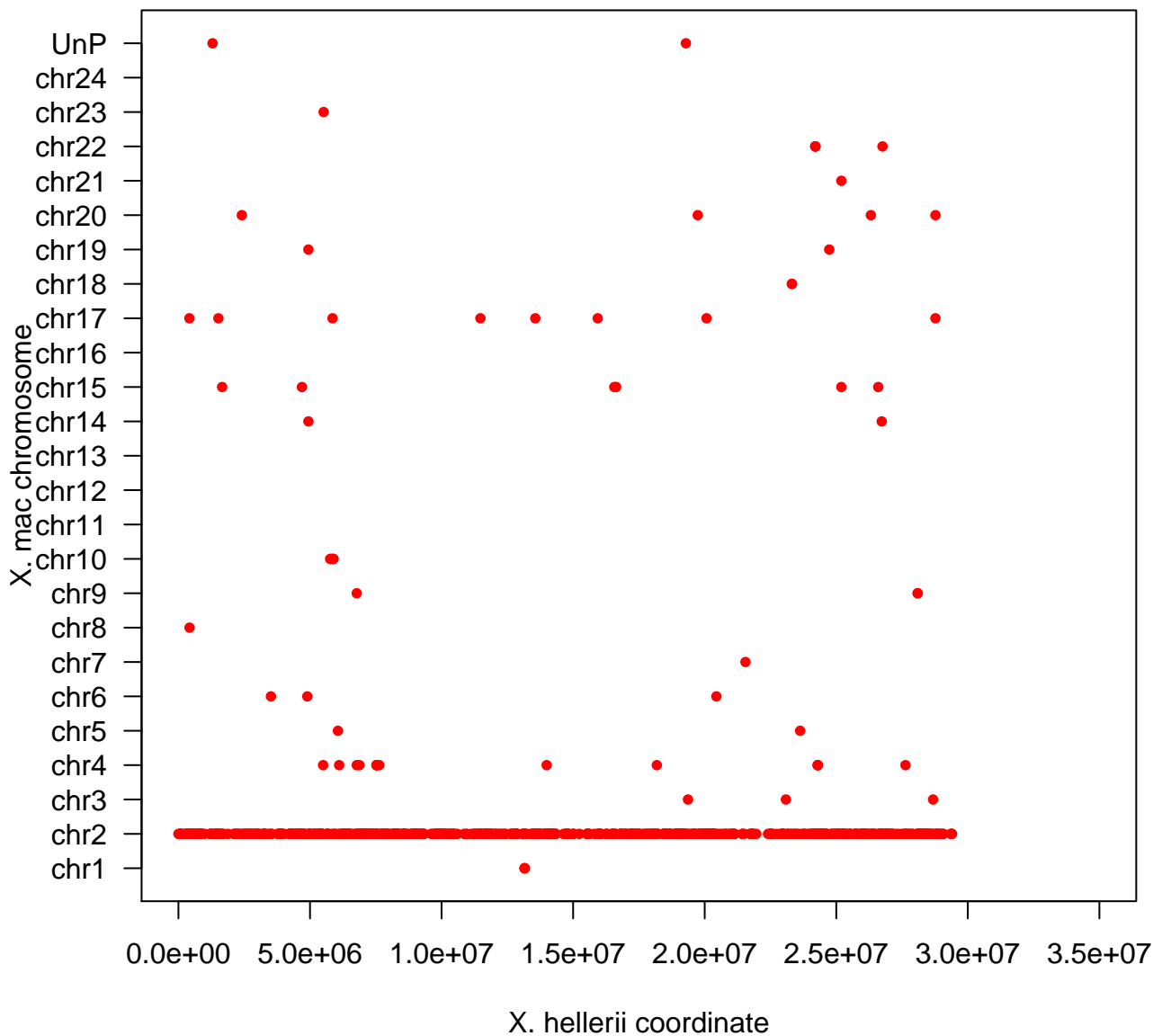

# LG20

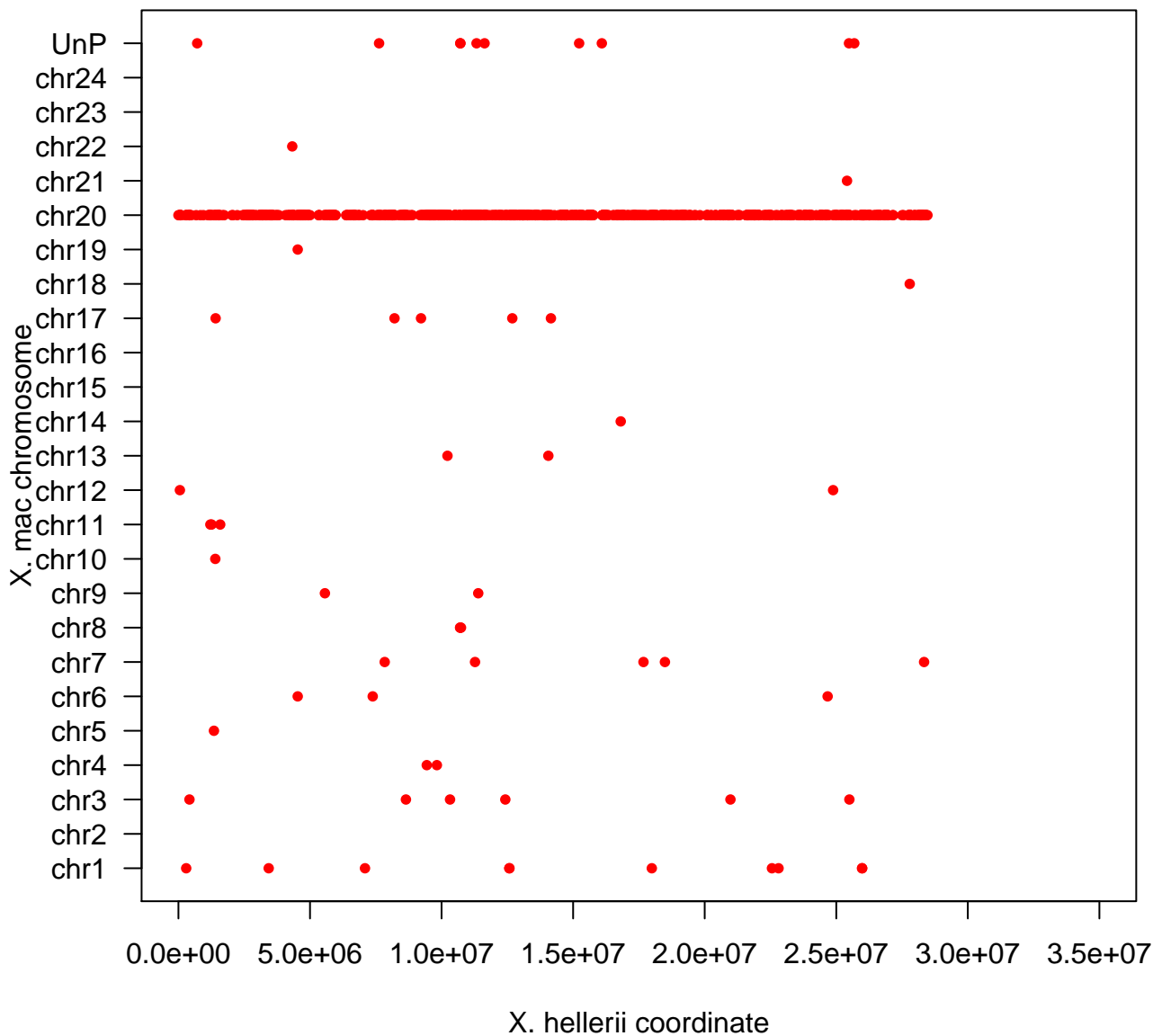

# LG21

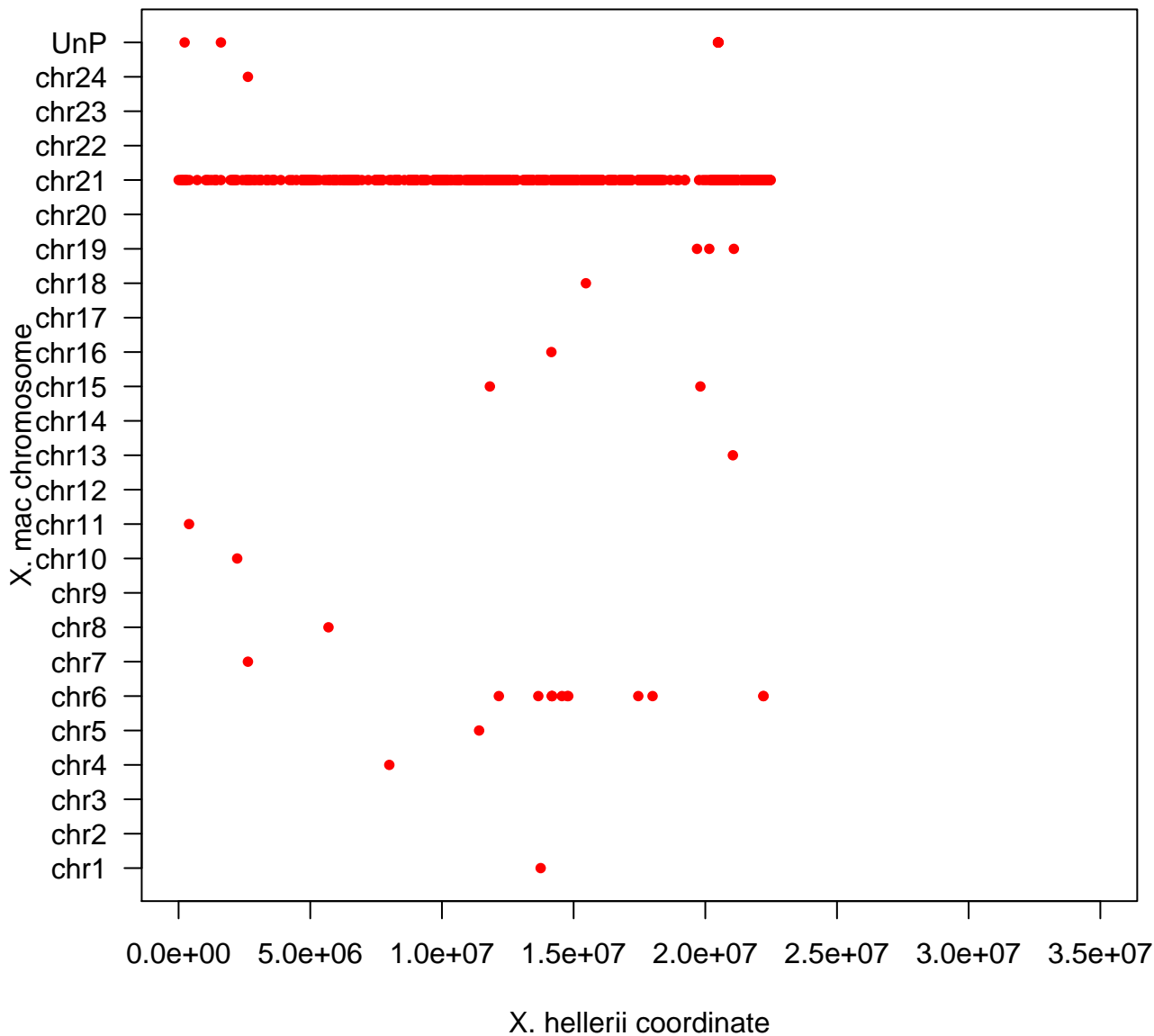

# LG22

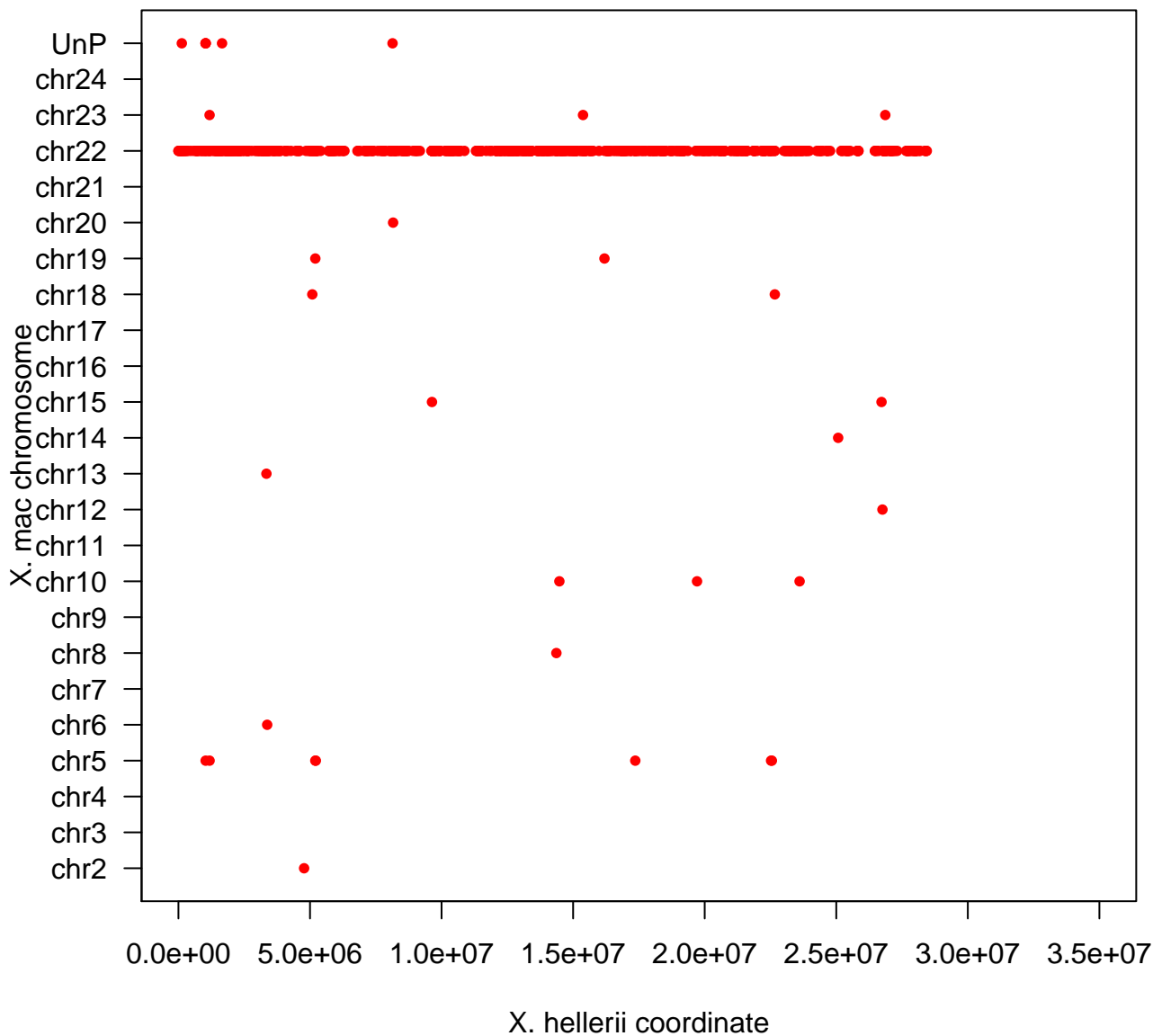

# LG23

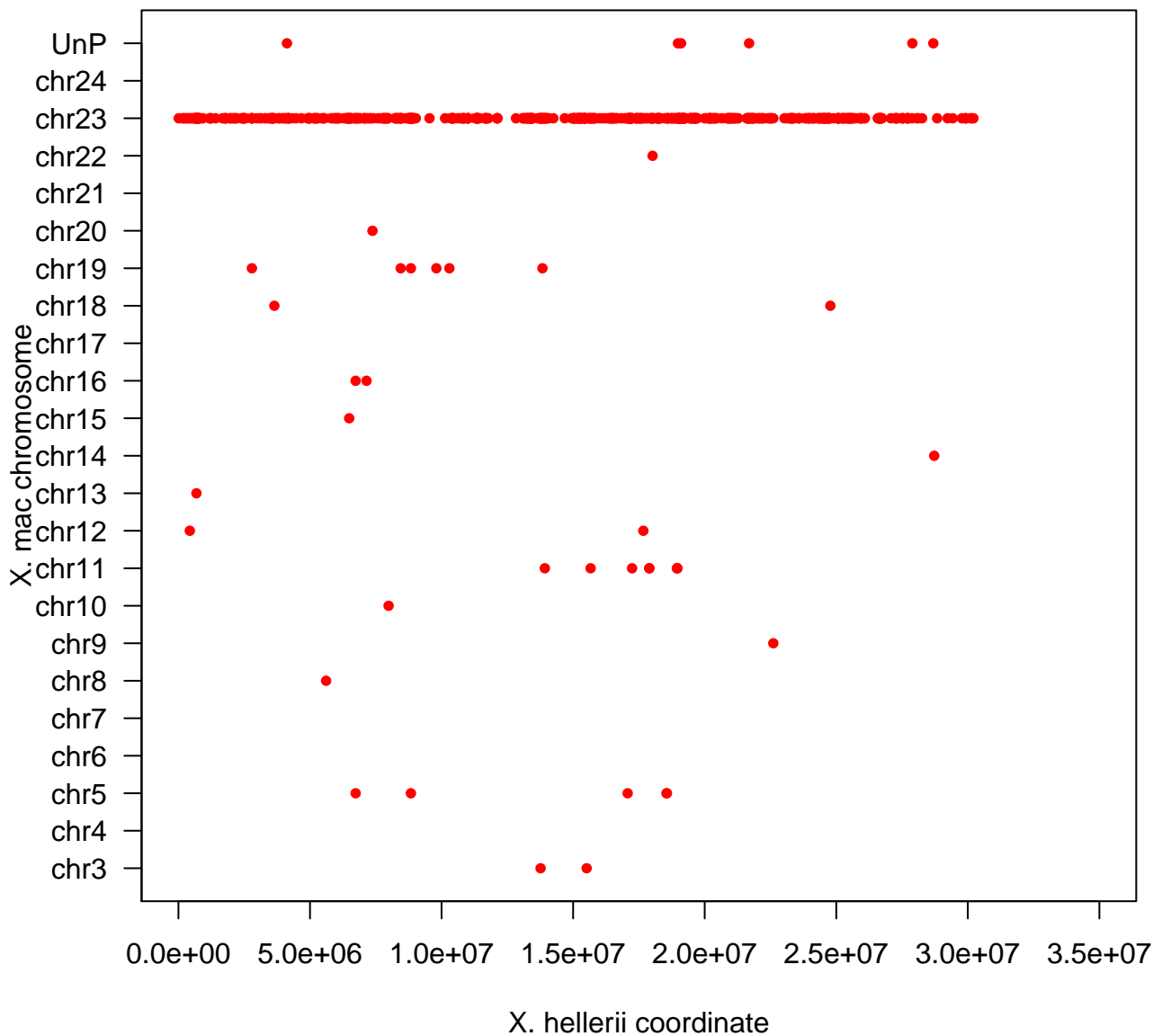

# LG24

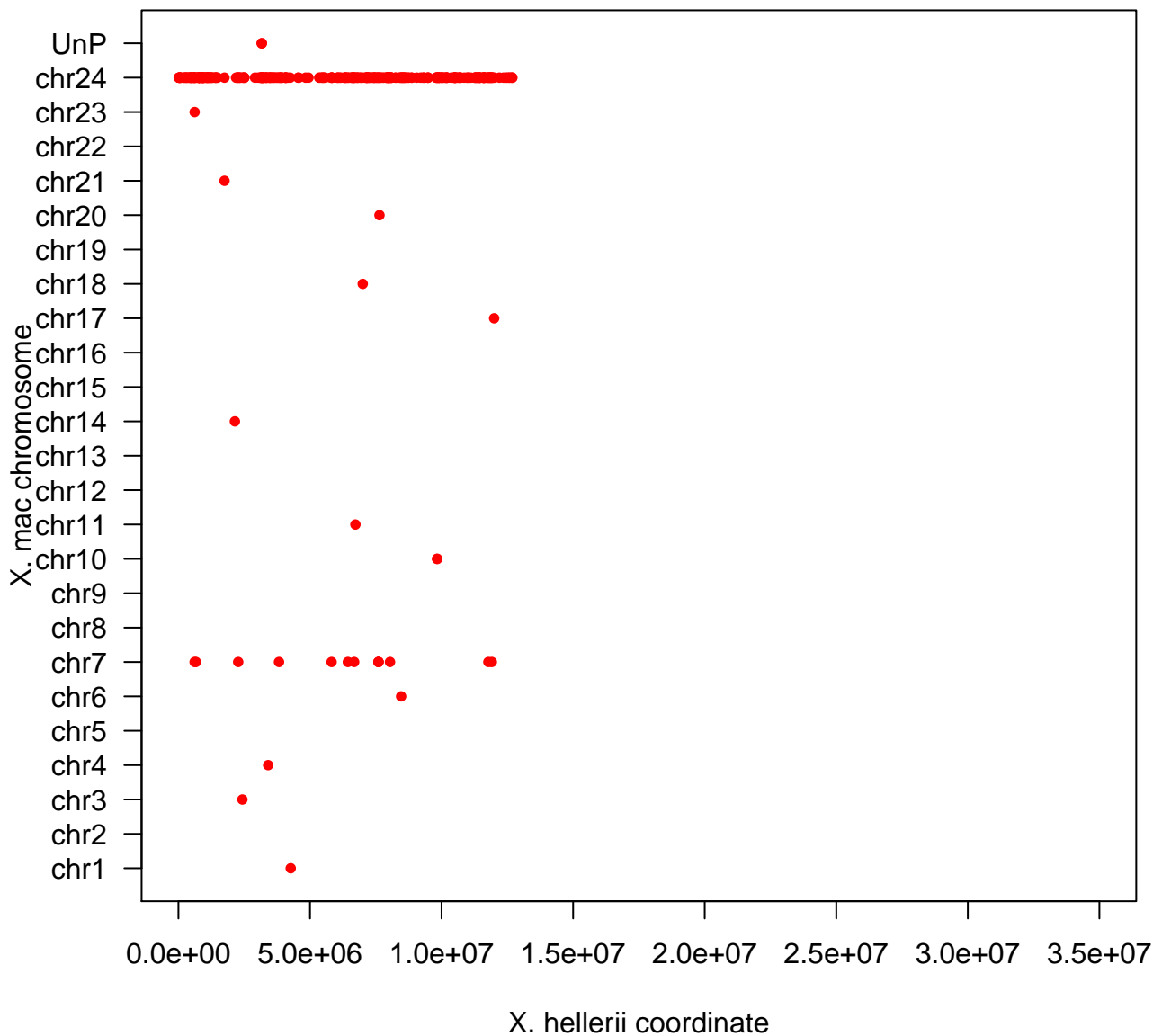

# LG3

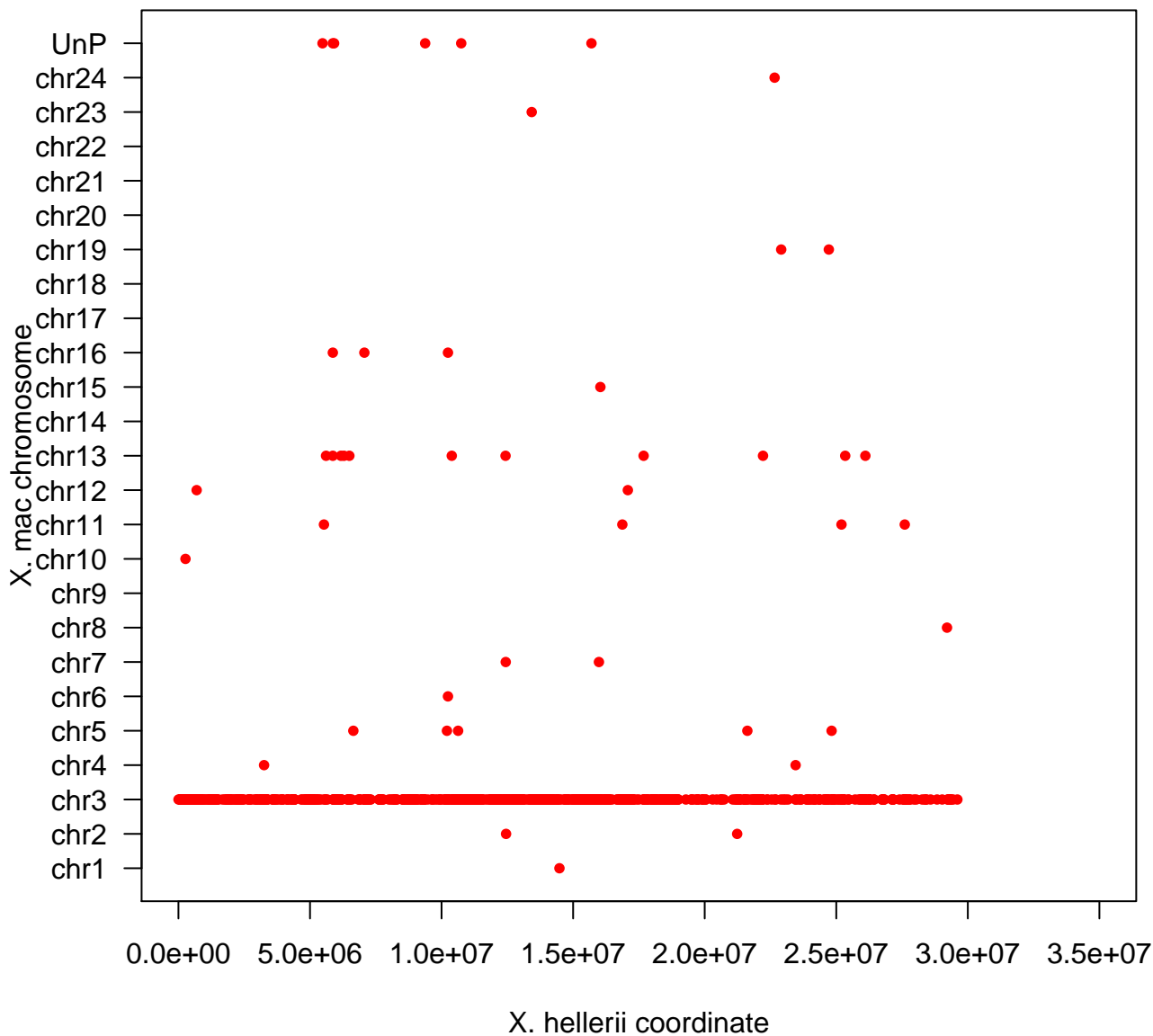

# LG4

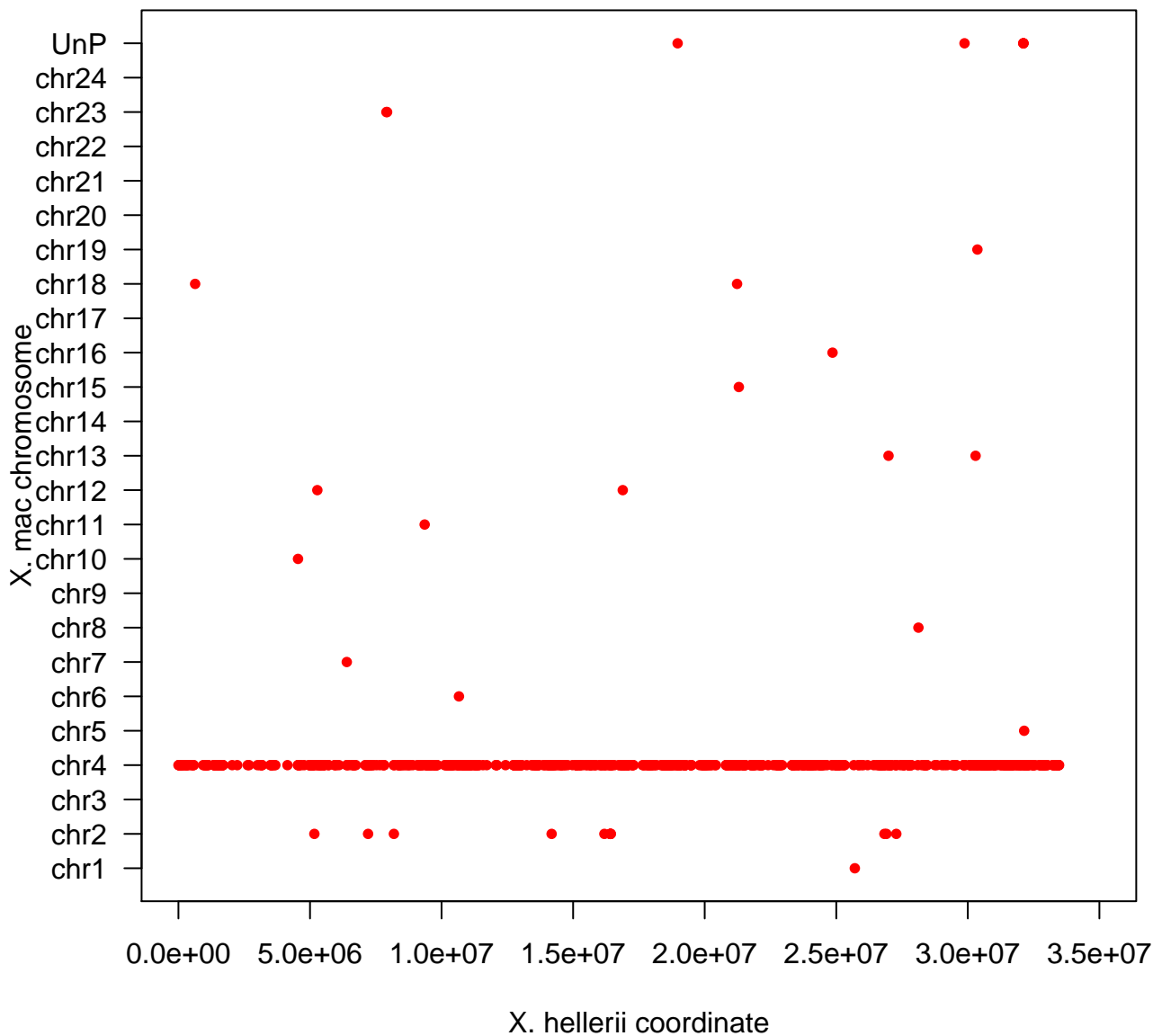

# LG5

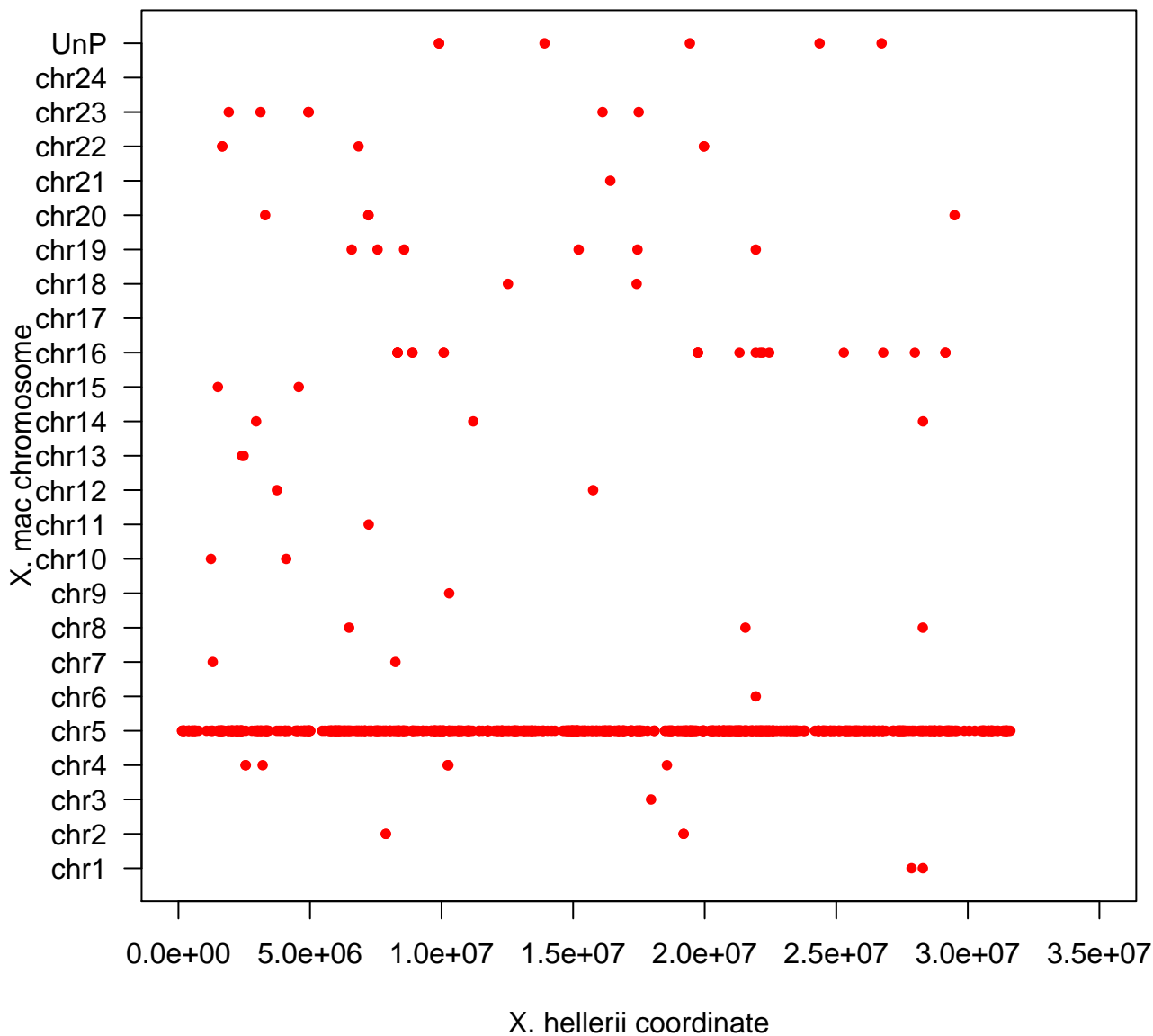

# LG6

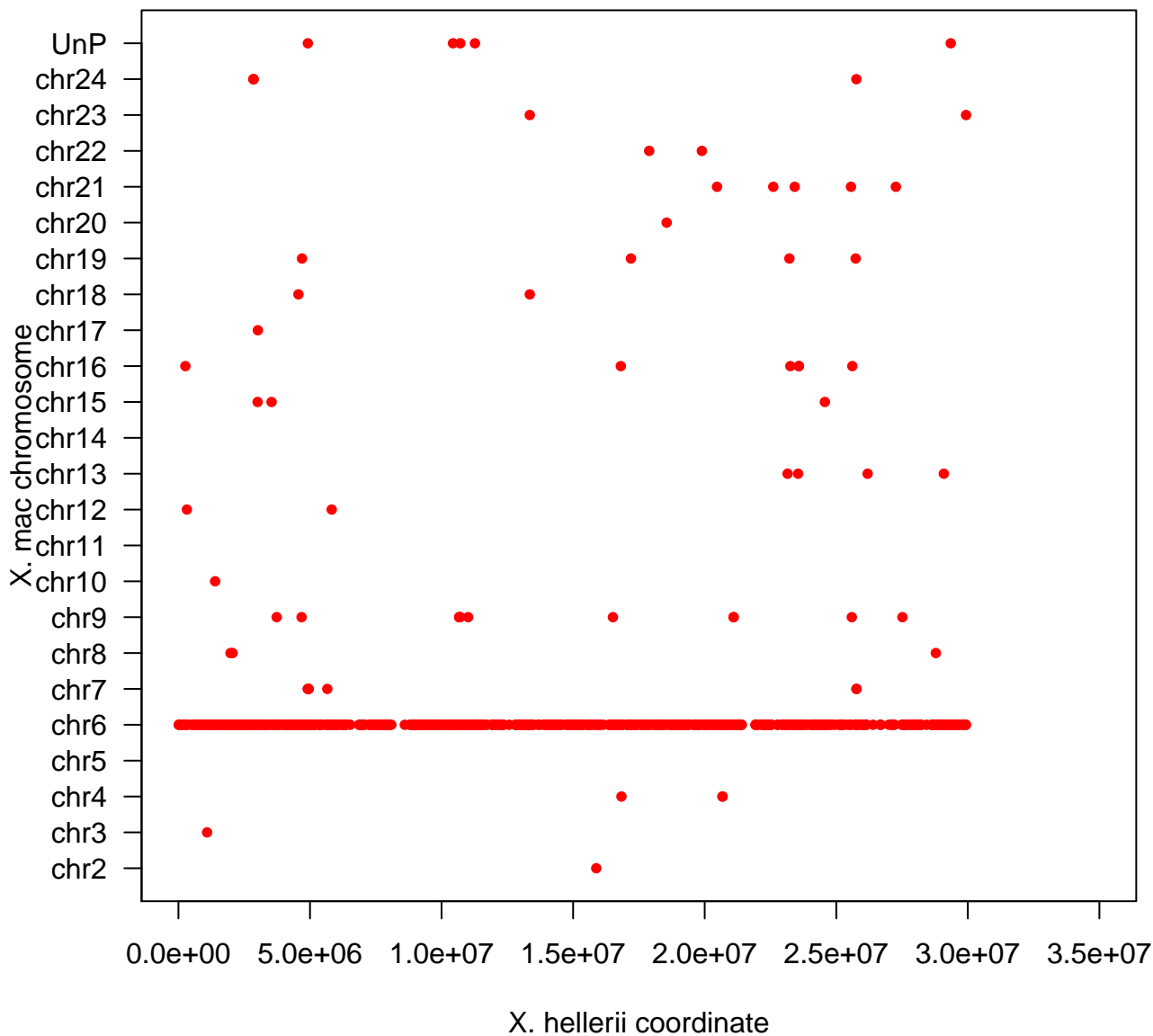

# LG7

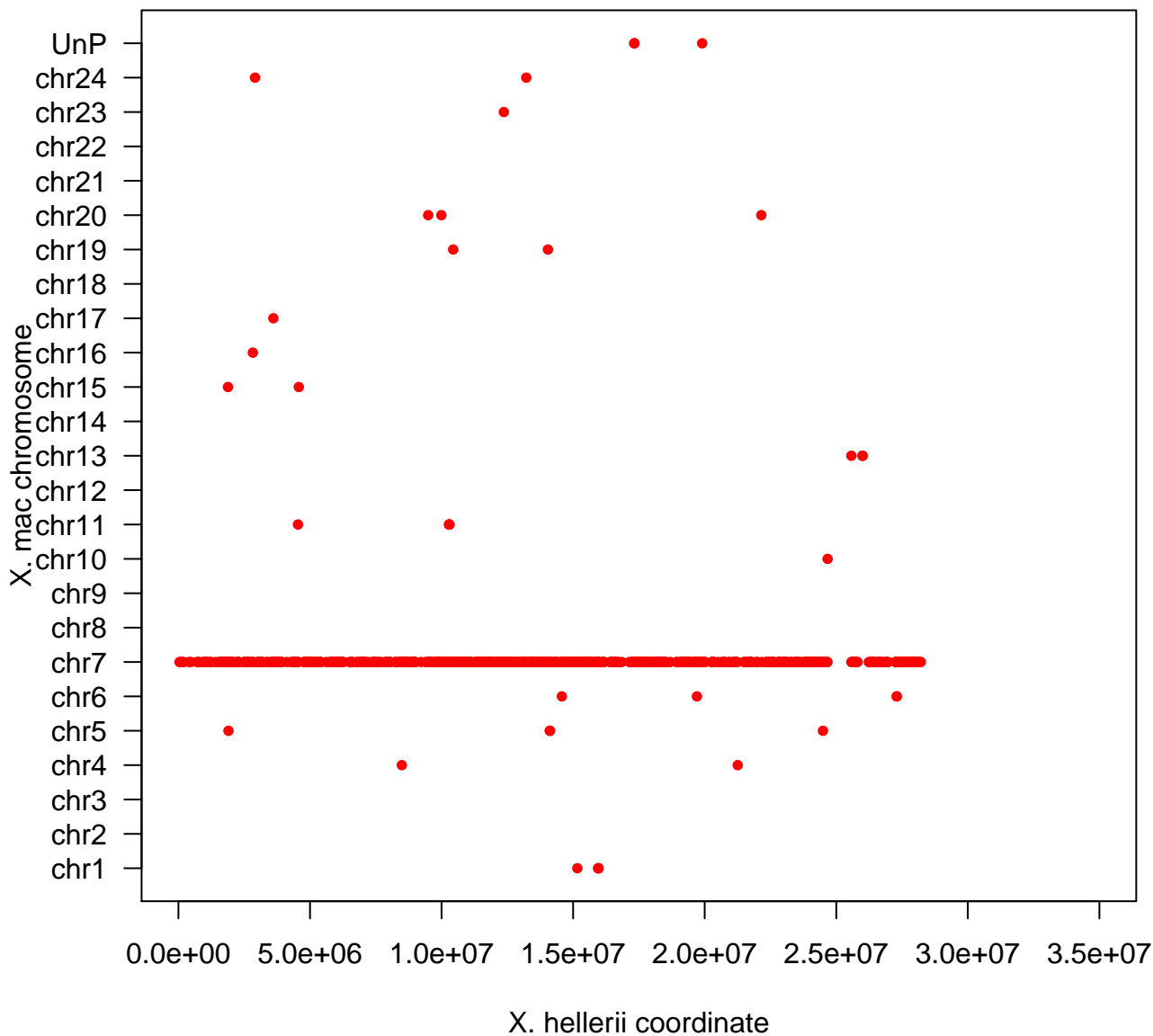

# LG8

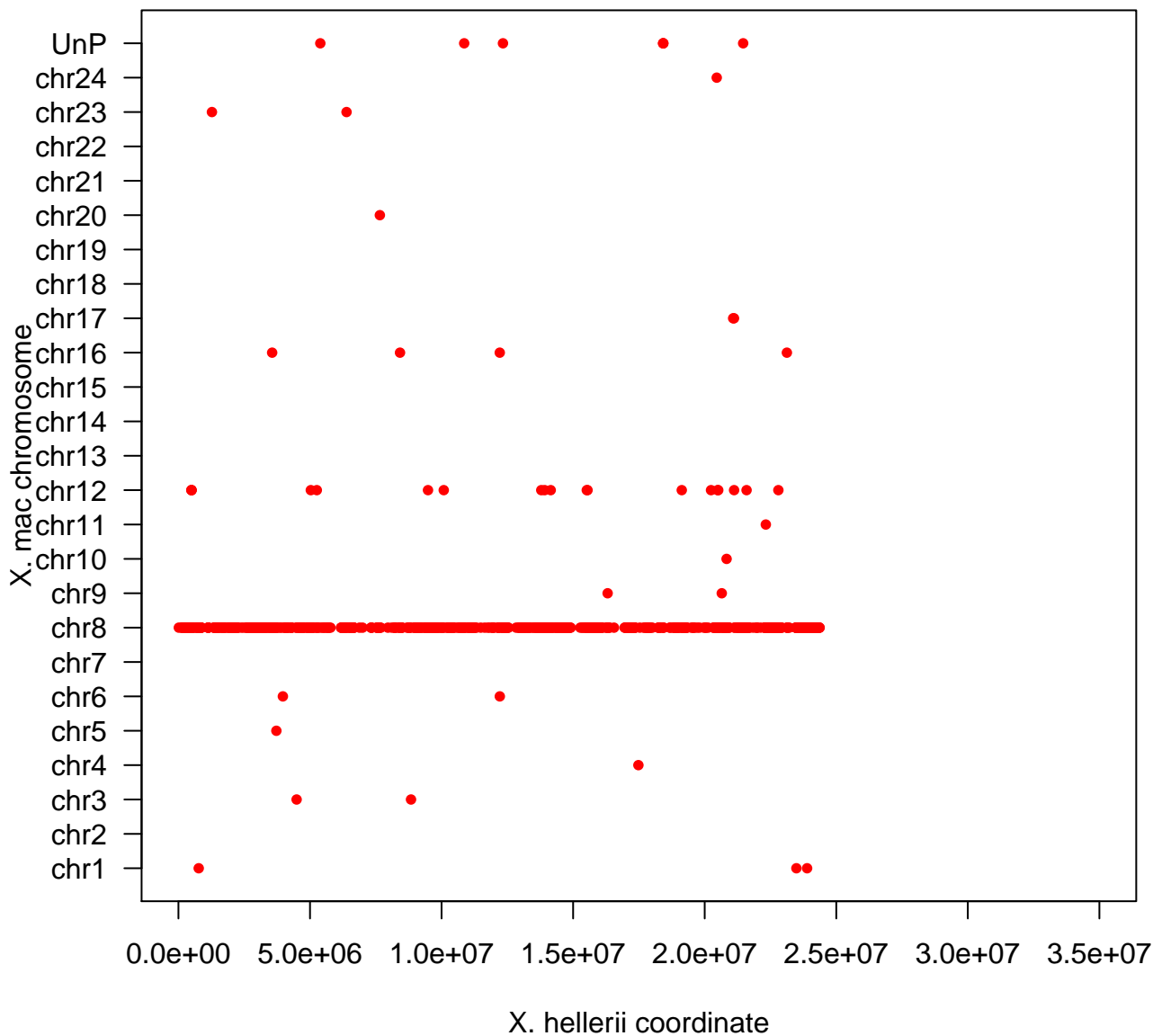

# LG9

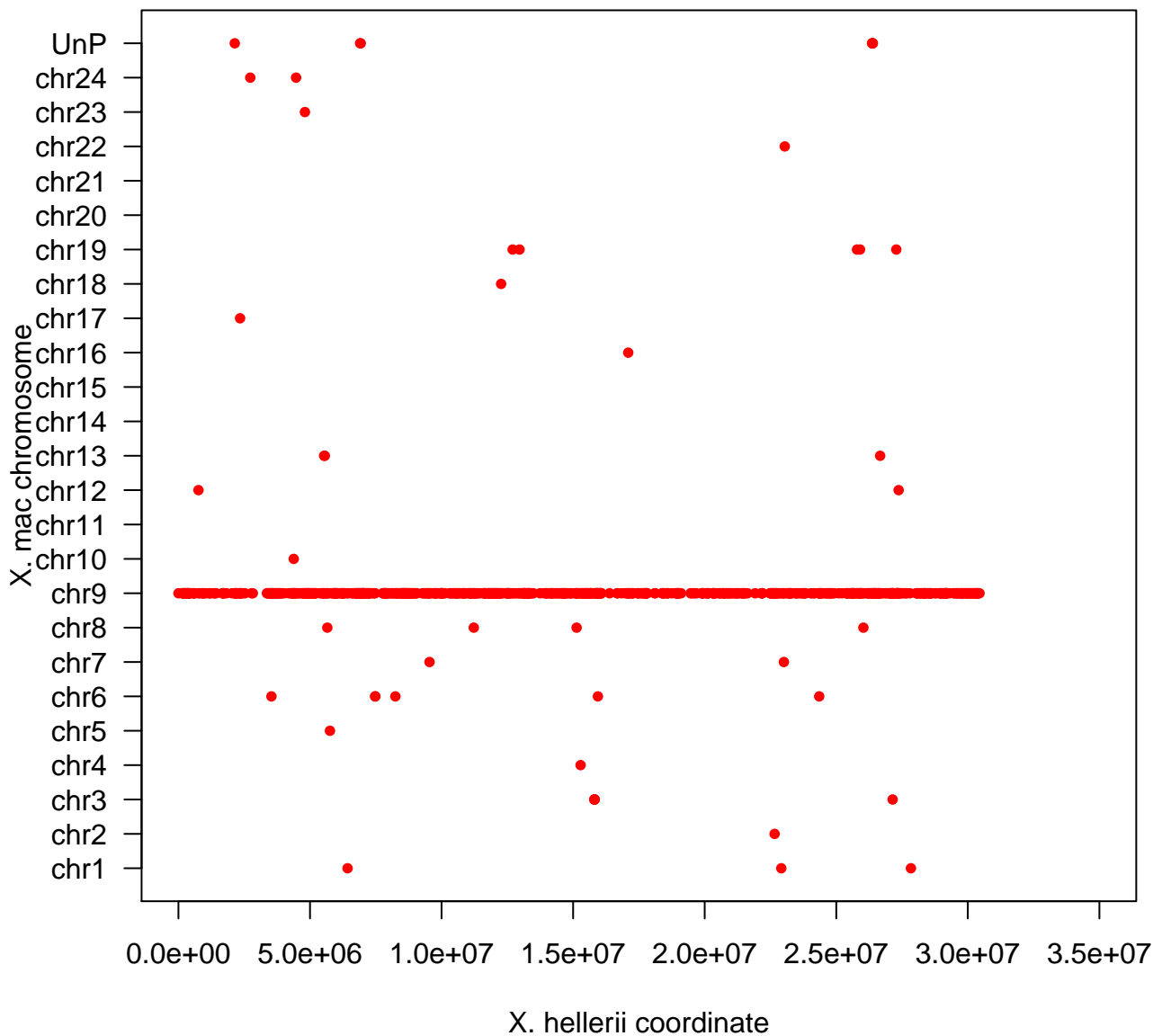

# unplaced

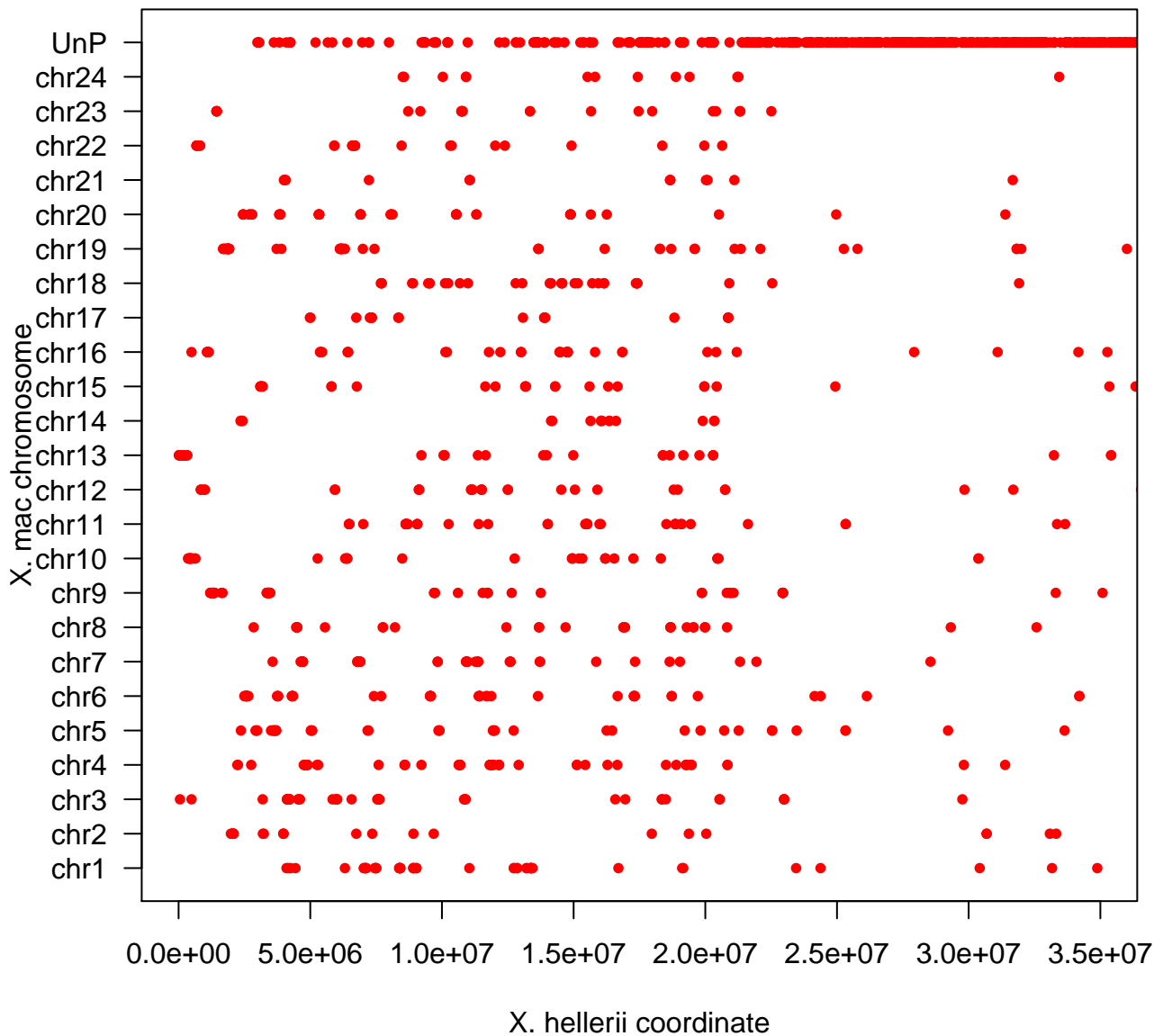

Supplement: Additional file 7: Figure S3. — Dot plots of location of one-to-one orthologues in the chromosomes of X. hellerii and X. maculatus. (PDF 111 kb) [file 12864_2015_2361_MOESM7_ESM.pdf]
